# Microwave-Assisted Automated Glycan Assembly

**DOI:** 10.1021/jacs.1c03851

**Published:** 2021-06-01

**Authors:** José Danglad-Flores, Sabrina Leichnitz, Eric T. Sletten, A. Abragam Joseph, Klaus Bienert, Kim Le Mai Hoang, Peter H. Seeberger

**Affiliations:** † Department of Biomolecular Systems, Max-Planck-Institute of Colloids and Interfaces, 14476 Potsdam, Germany; ‡ Institute of Chemistry and Biochemistry, Freie Universität Berlin, 14195 Berlin, Germany; § Max-Planck-Institute of Colloids and Interfaces, 14476 Potsdam, Germany; ⊥ GlycoUniverse GmbH & Co KGaA, Am Mühlenberg 11, 14476 Potsdam, Germany

## Abstract

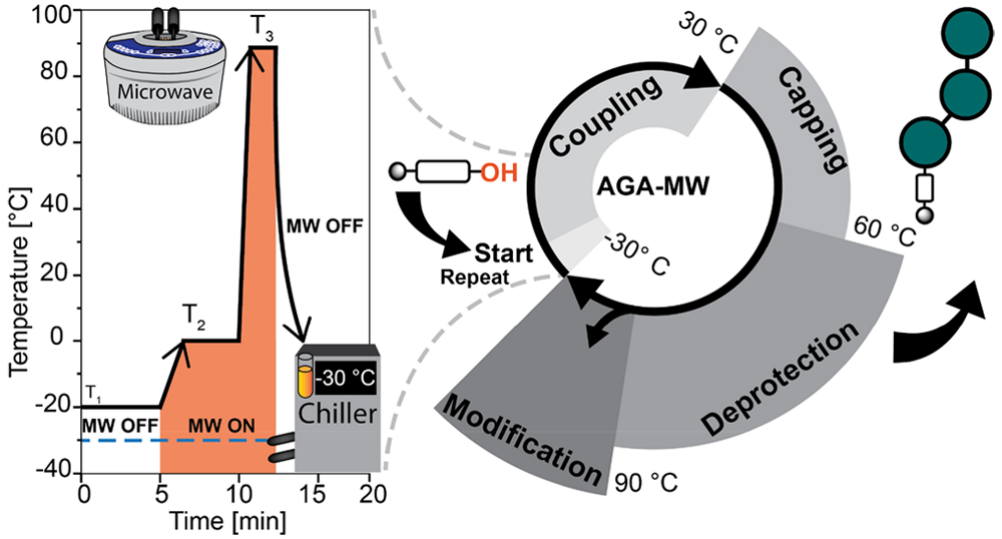

Automated synthesis of DNA, RNA, and peptides provides quickly and reliably important tools for biomedical research. Automated glycan assembly (AGA) is significantly more challenging, as highly branched carbohydrates require strict regio- and stereocontrol during synthesis. A new AGA synthesizer enables rapid temperature adjustment from −40 to +100 °C to control glycosylations at low temperature and accelerates capping, protecting group removal, and glycan modifications using elevated temperatures. Thereby, the temporary protecting group portfolio is extended from two to four orthogonal groups that give rise to oligosaccharides with up to four branches. In addition, sulfated glycans and unprotected glycans can be prepared. The new design reduces the typical coupling cycles from 100 to 60 min while expanding the range of accessible glycans. The instrument drastically shortens and generalizes the synthesis of carbohydrates for use in biomedical and material science.

## Introduction

**Figure 1:**
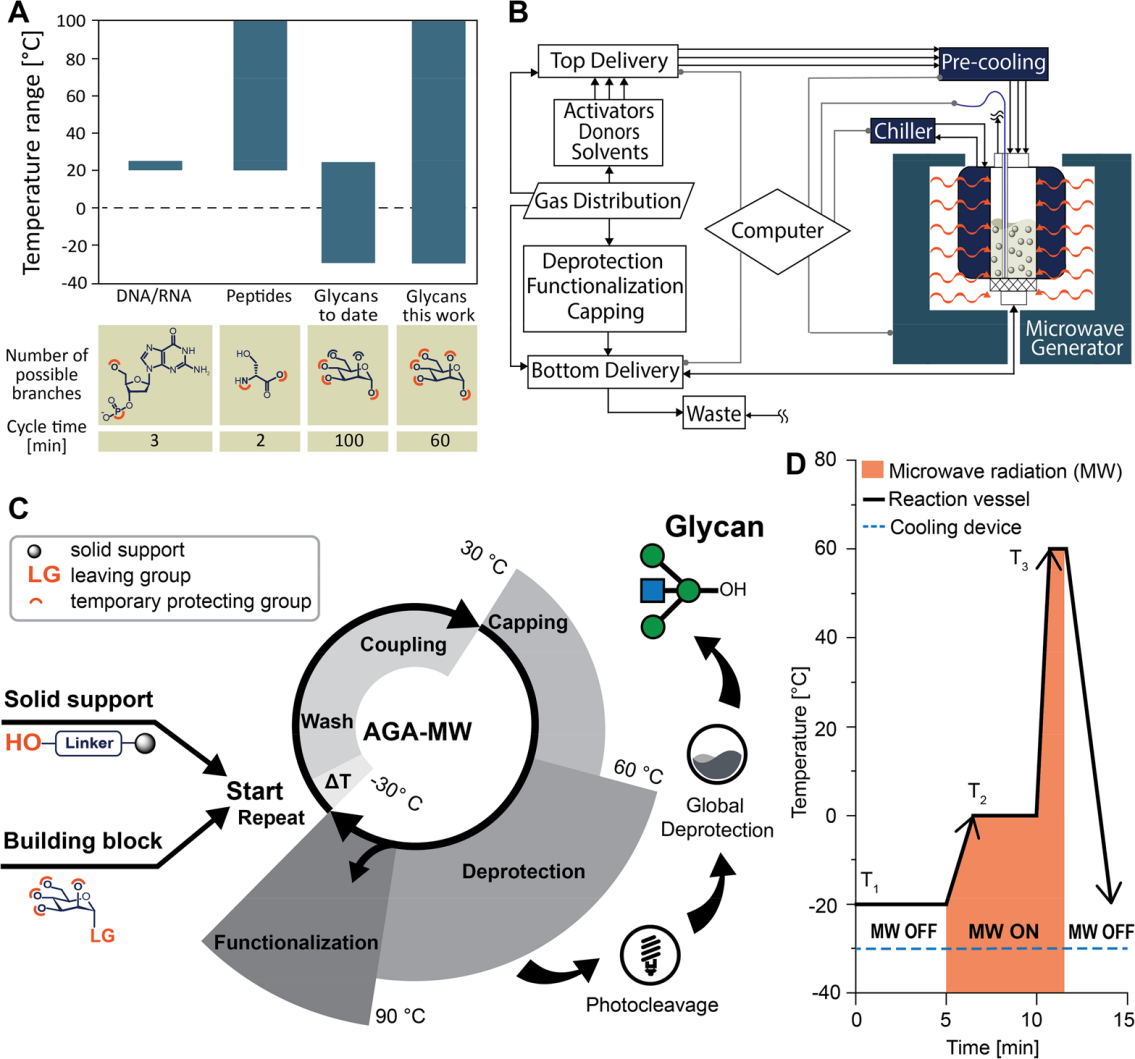
(A) Automated solid-phase synthesis of biomolecules. To date, AGA has been performed at low or ambient temperatures, and this work now expands the temperature range from −40 to +100 °C. (B) Schematic representation of microwave-assisted AGA system. (C) Flowchart of standardized AGA-MW process: start (baseline temperature adjustment), acid wash and coupling, capping, deprotection reaction, including automated postassembly modifications. The radial axis indicates the temperature range of the step. (D) Representation of the temperature profile regulated by the dual temperature regulation reactor system.

Automated chemical synthesis on a solid support allows rapid access to homogeneous oligonucleotides,^1,2^ peptides,^3^ and oligosaccharides.^4,5^ The interplay of technical advances and improved chemical methods has simplified and accelerated oligonucleotide and peptide synthesis. Automated glycan assembly (AGA) faces greater challenges considering the complexity of glycans that require stereo- and regiocontrol during the synthesis. Polysaccharides as long as 100-mers^6,7^ as well as complex glycans containing different building blocks and linkages are accessible by AGA.^4^ Technical limitations of the current AGA synthesizer restrict assembly speed, and the diversity of the glycan motifs can be prepared. Peptides and oligonucleotides are linear and require no stereocontrol during bond formation (Figure 1A). Oligonucleotides are readily synthesized at room temperature (cycle time = 3 min),^8,9^ while peptide construction^10^ is drastically accelerated at elevated temperatures (cycle time = 2 min, +70 to 100 °C). In contrast, glycosylation reactions are very temperature sensitive, thus they require low temperatures for high yields and selectivities. In a typical AGA cycle, the low-temperature glycosylations are followed by capping of deletion sequences and protecting group removal at room temperature (cycle time = 100 min). To date, the reaction temperature of AGA instruments^4^ is adjusted using a dynamic temperature control system connected to a jacketed reaction vessel. This thermoregulation system is inefficient when significant temperature differences are required during a synthesis cycle.^11^ Most importantly, the synthesis of branched glycans requires several orthogonal temporary protecting groups, though the working temperature range of −30 to +25 °C restricts AGA to just two types of routinely used temporary protecting groups: 9-fluorenylmethyl carbonate (Fmoc) and levulinoyl esters (Lev) that can be cleaved in under 1.5 h.^12^ Access to highly branched glycans has not yet been possible by AGA to date, and the synthesis of regioselectively sulfated glycans required specialized instrumentation and very long reaction times.^13,14^

Here, we describe a technological advance that drastically impacts the chemical transformations amenable to AGA (Figure 1B). Dual thermoregulation through the integration of a microwave (MW) generator and a jacketed reaction vessel permits almost instant temperature changes over a wide range (−40 to +100 °C) with minimal energy consumption. This new instrument helps to significantly shorten the time required for the incorporation of each building block and expands the portfolio of orthogonal temporary protecting groups as well as glycan modifications.

## Results and Discussion

### Instrument Design and Dual Temperature Regulation Reaction System

The key requirement for the new AGA instrument was quick and accurate adjustment of the reaction temperature over a wide range. The combination of the constant cooling action of a jacketed reaction with the heating power of microwave irradiation allows for rapid and accurate temperature adjustments over a broad range of temperatures during the entire AGA synthesis cycle (Figure 1C).^15^ With the dual temperature regulation reaction (DTRR) system, the fritted reaction vessel containing the solid support is cooled to the lowest temperature required during the synthesis using a circulating coolant set at the baseline subzero temperature (Figure 1D). The temperature in the reaction vessel then is adjusted by microwave radiation. Throughout the heating phase, the temperature of the microwave-inert coolant in the jacket is unchanged and allows for rapid return to subzero temperatures. This design stands in stark contrast to coolable microwave reactors that operate at constant temperatures and are not able to perform iterative/sequential multistep syntheses.^16,17^

The reaction vessel of the DTRR system is supplied with reagents by an automated delivery system capable of handling gas and liquids with a wide range of viscosities, vapor pressures, and pH (Figure 1B). Building blocks, activators, and washing solutions enter through the top of the reaction vessel. The temperature of these reagents is adjusted to the reaction temperature prior to addition using a precooling heat exchanger to avoid temperature spikes due to warm reagents during glycosylations that tend to be very temperature sensitive. Capping and deprotection reagents are added from the bottom of the reaction chamber. In addition, gas to mix the reagents is added from the bottom and houses the effluent outlet. The coupling, deprotection, and capping reagents are incompatible and were separated into two systems (top delivery and bottom delivery) to avoid cross-contamination. The addition of building blocks and activators is controlled via a syringe pump to ensure maximum accuracy (1 ± 0.05 mL), while all other reagents are added to the reaction chamber by argon overpressure. The gas distribution system provides a separate inert atmosphere to each reagent group. The gas blanket prevents reagent degradation and cross-contamination by invasive reagents and drives several of the reagents through the system.

**Figure 2:**
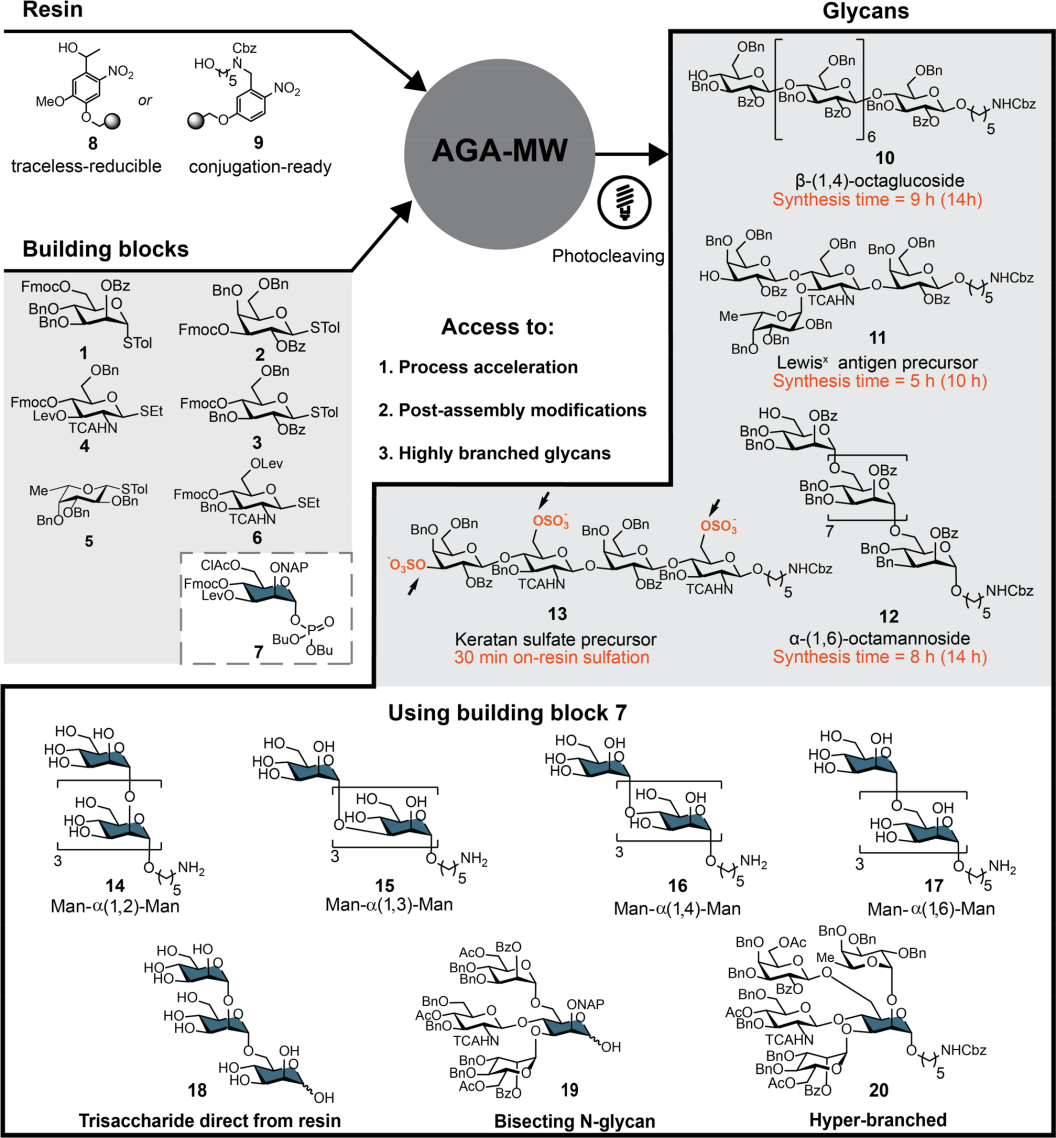
Oligosaccharides **10**–**20** prepared to highlight the capabilities of an AGA-MW synthesizer. Time in parentheses notes the synthesis time using a standard AGA instrument.

The delivery system and temperature regulation components are connected to a computer that controls the device operation (Figure 1B). The synthesis steps are programmed using a software developed for this purpose. Operative modules are selected, and the process parameters are modified including building block position, temperature, incubation time, step iterations, reagent volume, and microwave power to prepare the desired glycan sequence. Importantly, the system contains a built-in fiber-optic thermoprobe for the real-time temperature monitoring inside of the reaction vessel to aid in optimizing the AGA cycle. Figure 2 presents several example of improved and newly developed oligosaccharide synthesis.

**Figure 3:**
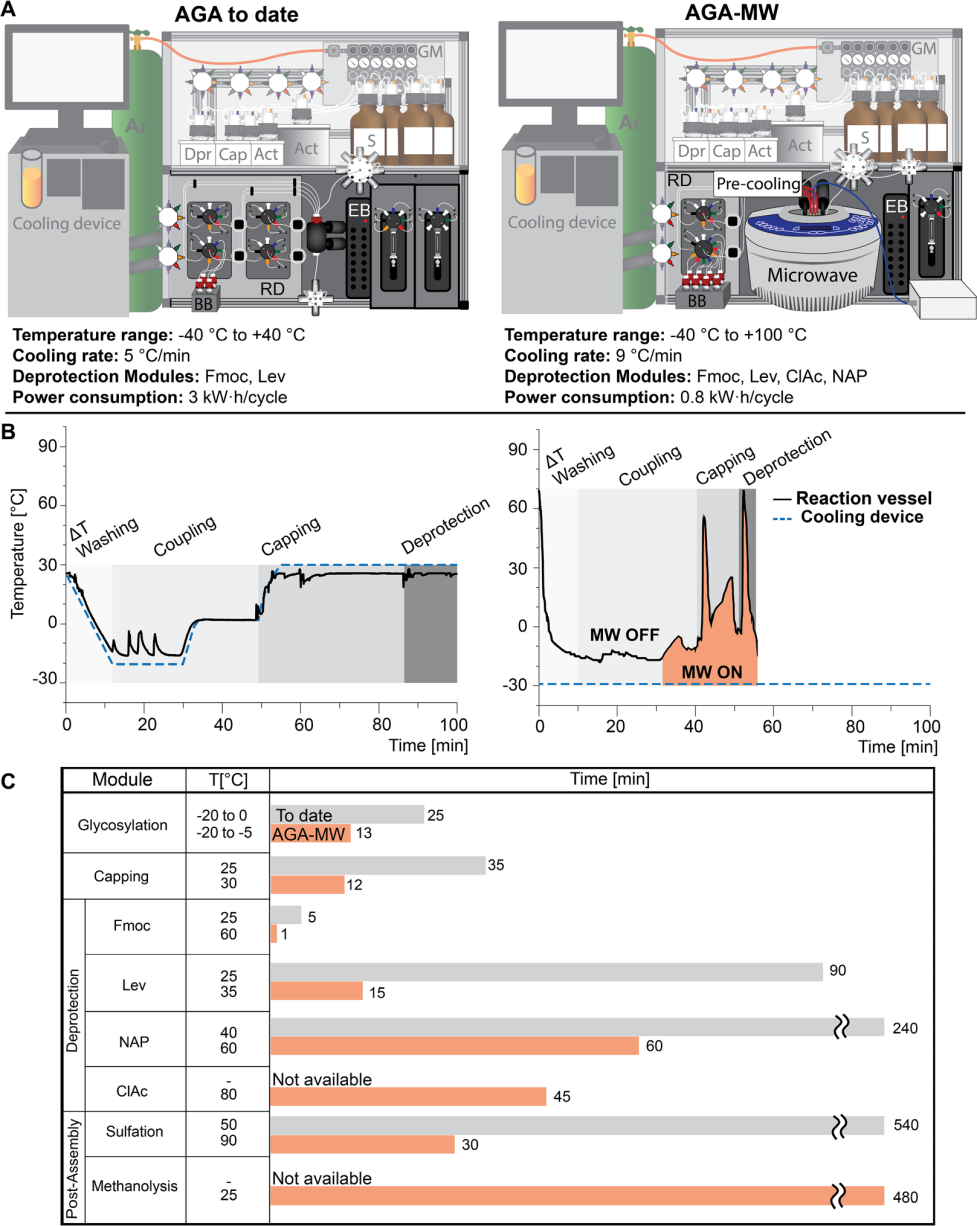
Comparison of the previous state of the art and DTRR synthesizer based on AGA-MW. (A) Pictorial representation and technical data (EB = electronic board, GM = gas manifold, and RD = reagents distribution). (B) Temperature profile inside the reaction vessel during one coupling/capping/deprotection cycle of 12 (right) compared to that of one cycle in a standard AGA instrument. (C) Comparison of modules in the standard system (without microwave) and the DTRR system (with microwave).

The dual temperature regulation system consumes less than half the energy when compared to the previous single device thermoregulation system (0.8 vs 3 kW·h/cycle). Moreover, to accelerate the regulation to subzero temperatures and suppress thermal spikes, a “precooling” heat exchanger unit (Figure 1B and Figure 3A,B) cools the solvents (S), building block (BB), and activator solutions (Act) prior to reaching the reaction vessel (Figure 3A).

### Process Acceleration

The syntheses of linear β-(1,4)-octaglucoside **10**, branched Lewis^X^ antigen^18^ tetrasaccharide precursor **11**, and α-(1,6)-octamannoside **12** serve to illustrate the utility of the DTRR synthesizer (Figure 2). Solid support resin **9** is placed in the reaction vessel, and the reaction temperature is adjusted to the baseline subzero temperature before washing with a series of solvents and a neutralizing acidic (Figure 1C). This acid wash is particularly important during subsequent coupling cycles to ensure the removal of any remaining base. Glycan elongation begins with mixing the resin, the glycosyl building block (BB), and activator at the baseline temperature (*T*_1_) for a desired time (*t*_1_), before the temperature of the reaction solution is linearly increased (ramp = 4 °C/min) to the next thermal stage (*T*_2_) by microwave radiation and held for a desired period of time (*t*_2_) (Figure 1D). Incubation times and temperatures were characteristic of the donor and acceptor, optimized from reported conditions. Microwave radiation permitted the rapid temperature adjustment in a series of reactions (right side Figure 3B). Upon draining and washing, unreacted support-bound hydroxy nucleophiles are capped by acetylation at 25 °C.

Removal of the temporary protecting group can then rapidly proceed at elevated temperatures. The DTRR system is effective at reaching temperatures above 25 °C and to promptly return to subzero temperatures for the next glycosylation (Figure 3B). During the synthesis of the linear α-(1,6)-octamannoside **12** and β-(1,4)-octaglucoside **10** (Figure 2), Fmoc cleavage was achieved in 1 min at 60 °C using 20% piperidine, with the temperature returning to −20 °C within 10 min (Figure 3B,C). The next coupling cycle then starts upon return to the baseline temperature, and the process is repeated until the desired oligosaccharide sequence has been prepared. These octasaccharides were prepared in less than 9 h from the corresponding thioglycoside building blocks **1** and **3** (Figure 2, **12** = 45% and **10** = 41% yield), thereby drastically lowering the overall cycle time from the previous required 100 min for each monosaccharide incorporation with no reduction in yield (Figure 3B).^11^ Further process acceleration resulted in reduced yields, as most of the remaining time is required for thorough resin washes between switching from acidic to basic conditions throughout the cycle.

The synthesis of branched Lewis^X^ antigen^18^ tetrasaccharide **11** required two temporary protecting groups, Fmoc and Lev ester (Figure 2). During the synthesis, the C-3 Lev ester group on the glucosamine **4** residue was first selectively removed with hydrazine acetate in 15 min at 35 °C (Figure 3C) in anticipation of fucosylation. Following the addition of **5**, the C-4 Fmoc was removed to prepare for the subsequent galactosylation by **2** to afford **11** in 6 h and 53% yield. Levulinoyl ester cleavage was accelerated by 75 min (90 min → 15 min), while, unfortunately, the unreactive glucosamine building block still required a double coupling to achieve high efficiencies. There was no significant difference in yield compared to reported syntheses.^11,18^

### Expansion of Orthogonal Temporary Protecting Group Portfolio

Carbohydrates, other than peptides and oligonucleotides, are branched. Therefore, the chemical synthesis of branched oligo- and polysaccharides requires more than one temporary protecting group. Orthogonal protecting groups allow for unmasking of one hydroxy group in anticipation of elongation without affecting protecting groups at other positions. The ability to adjust the temperature in the DTRR system quickly to higher temperatures creates new opportunities for the use of orthogonal protecting groups that were previously not amenable to AGA. In order to create heavily branched glycans, ideally four orthogonal protecting groups are required. For this purpose, differentially protected mannose building block **7** was designed (Figure 2). Few other completely orthogonal building blocks have been reported,^19−22^ yet none are amendable to AGA as many considerations have to be taken into account, such as tolerance of protecting groups to AGA conditions, total solubility of reagents and byproducts, fast and selective deprotection process, as well as reactivity of the building block itself. Therefore, the protecting groups and their positions in **7** were chosen carefully for optimal performance in AGA. From the plethora of protecting groups that have been developed for solution phase chemistry, Fmoc, Lev, 2-naphthylmethyl ether (NAP), and chloroacetate ester (ClAc) were selected as orthogonal protecting groups^12,19^ to be used for AGA. Fmoc and Lev had already shown promise for rapid cleavage in the DTRR system, though it was found that repeated exposure of the Lev group to piperidine resulted in side reactions. The simple switch to triethylamine resolved the issue (Figure 3C). The Lev group was positioned at the C-3 as O-3 ester groups are reported to assist with strong α-selectivity.^23^ It was found that the Fmoc was best positioned at the C-4 position, as a C-4 AcCl group tended to migrate to the primary C-6 hydroxy group upon a C-6 Fmoc cleavage. As a result, the AcCl was used to protect the primary C-6 position in mannose building block **7**. Now capable of reaching 80 °C in the DTRR system—which was not achievable in the standard AGA system—the deprotection of the AcCl group using thiourea was facilitated in 45 min (Figure 3C).^19^ To balance the electron-withdrawing ester and carbonate groups (Fmoc, AcCl, and Lev), the electron-donating NAP group was chosen to protect the C-2 position to ensure proper reactivity of **7** when serving as both a glycosyl donor and as an acceptor. Even with the C-2 nonparticipating NAP ether group, complete α-stereoselectivity was observed for building block **7**. To date, NAP ethers have been used in AGA,^24^ but their cleavage is very slow, sometimes incomplete, and accompanied by side products, arising from debenzylation. While in the previous system the NAP group removal required an impractical 240 min using DDQ/MeOH/water in DCE at 40 °C, the DTRR system provides more practical and reliable NAP deprotections in 60 min at 60 °C using DDQ/MeOH in DCE (Figure 3C). Acidic conditions^25,26^ failed to selectively cleave the NAP ether in the presence of Lev, AcCl, and Fmoc.

To illustrate the concept, α-(1,2), α-(1,3), α-(1,4), and α-(1,6)-tetramannosides **14**–**17** were assembled from building block **7** (Figure 2, yields α-(1,2) **14** = 19%, α-(1,3) **15** = 12%, α-(1,4) **16**, α-(1,6) **17** = 18%) with excellent regio- and stereoselectivity. Encouraged by these results, *P. falciparum* GPI anchor mannose trisaccharide portion **18**(27) containing both α-(1,2) and α-(1,6) linkages was achieved on polystyrene resin equipped with traceless-reducible photolabile linker **8**. For that, the NAP group at the first sugar unit was removed and acetylated before extending at C-6 and then the C-2 position. After the assembly, solid support-bound unprotected oligosaccharide was prepared by removing the remaining NAP ether, followed by methanolysis using sodium methoxide (Figures 3C and S2). The global deprotection at the solid support was confirmed by on-resin Fourier transform infrared spectroscopy (see Supporting Information). Photocleavage of the linker released the trisaccharide product **18** from the solid support (yield = 15%). This oligosaccharide is the first example of a natural, unmodified glycan produced by AGA without any manual protecting group manipulations, otherwise known only from fully enzymatic or chemoenzymatic approaches. The syntheses of **14**–**17** as well as **18** also show that a single building block such as **7** can give rise to many possible combinations of oligosaccharides.

### Synthesis of Highly Branched Glycans

Naturally occurring glycans are frequently branched, and in rare cases, such as of chorella viruses, three branches extend from a single monosaccharide.^28^ For artificial systems, the creation of highly branched glycans where every hydroxy group serves as a point of modulation are also desirable. Orthogonally protected mannose building block **7** served in the assembly of branched structures including a portion of bisecting *N*-glycan **19** (Figure 2, yield = 28%). Hyperbranched, unnatural tetrasaccharide **20** containing four branches bearing fucose, mannose, *N*-acetylglucosamine, and galactose was assembled (Figure 2, yield = 32%). This highly branched tetrasaccharide illustrates the challenges associated with the analysis of such complex glycans. Characterization by NMR spectroscopy initially gave rise to concerns, as complex spectra seemed to suggest a failed synthesis. NMR analysis of the same samples at elevated temperature (see Supporting Information) revealed an entirely different picture and shows that such densely functionalized, protected oligosaccharides adopt structures containing different rotamers. This has been observed for other glycan structures before.^29,30^

### Postassembly Modification

Many naturally occurring glycans are modified by sulfation, acetylation, or lipidation. Glycosaminoglycans and many marine glycans, for example, are heavily sulfated.^31,32^ The synthesis and purification of sulfated glycans is challenging due to the high polarity and sulfate lability. Solid support synthesis of sulfated glycans is advantageous as it minimizes the number of purification steps.^13,14^ The automated construction of keratan sulfate tetrasaccharide **13** (Figure 2) served to illustrate microwave accelerated sulfation^33^ on the solid support. Following the assembly of a tetrasaccharide employing building blocks **2** and **6**, the levulinoyl esters were selectively cleaved to expose the C-6 hydroxy groups of the glucosamine residues as well as the C-3-hydroxy group in the terminal galactose by Fmoc removal. The three unprotected hydroxy groups were sulfated within 30 min by a sulfur trioxide trimethyl amine complex in DMF at 90 °C (Figures 3C and S2).^33^ This sulfation process was a significant improvement over the previous protocol that required 9 h.^13^ Salts and excess sulfation reagents were simply washed away from the resin such that, following photocleavage from the resin, routine reverse-phase chromatography purification yielded 24% of desired trisulfated tetrasaccharide **13**.

## Conclusion

Carbohydrates, the most abundant biomolecules in nature, are essential for structure, energy supply, and molecular interactions of living organisms.^34^ Rapid access to homogeneous glycans is essential for medical, biological, and material science investigations. Here, we report a technological advance, a new oligosaccharide synthesizer that broadens the range of possible synthetic transformations. Now, access to glycans with and without modifications is possible that were previously not accessible. The new AGA instrument combines microwave irradiation and constant cooling to allow for fast adjustments of temperatures from −40 up to +100 °C with minimal energy consumption. The versatility of the new instrument concerning temperature range and control has no precedent in AGA or automated solid support synthesis in general. The reaction vessel is supplied with reagents via a delivery system that can handle a wide variety of gases, acids, bases, and high vapor pressure solvents.

Previously, some carbohydrate transformations were performed using microwave radiation^16,35^ and even cooled microwave reactors.^36,37^ However, microwave heating has not been implemented to regulate several steps during an oligosaccharide synthesis or for AGA.^36^ Real-time monitoring of the rapid and accurate heating of the reaction mixture resulted in a significantly faster synthetic process, as illustrated by the assembly of several linear and branched oligosaccharides. The ability to heat and cool reactions quickly and reliably enables new chemical strategies for oligosaccharide assembly. A variety of temporary protecting groups can be cleaved on a solid support such that fully deprotected oligosaccharides are released upon cleavage from the solid support. With four temporary protecting groups available, the construction of highly branched glycans is now possible. Glycan modifications such as sulfation found in glycosaminoglycans and marine glycans are now quickly possible.

Simultaneous cooling and microwave heating improves reactor temperature control by preventing run away temperatures, eases postsynthesis vessel handling, modifies the nucleation and growth of solid products,^38−41^ and explores nonthermal microwave effects.^17,36,41^ Rapid adjustment to the optimal temperatures required during multistep syntheses is readily achieved in microwave-assisted reaction systems. By combining microwave dielectric heating and constant cooling, a wide temperature range for each reaction within a multistep synthesis is available such that complex molecules can be prepared within a single device. The use of optimized monosaccharide building blocks and further improved coupling cycles will help the new AGA synthesizer to prepare ever more complex glycans even faster. The modular nature of the new device makes it ideally suited for expansion to the synthesis of other complex molecules and not just carbohydrates.^42^

To further accelerate AGA, a better understanding of coupling conditions including optimal temperature and incubation times for each building block is required. Process intensification strategies will be explored to enhance both mass and heat transfer.

## Experimental Section

### Glycan Assembly

Automated syntheses were performed on a home-built synthesizer developed at the Max Planck Institute of Colloids and Interfaces. All details concerning preautomation steps, building blocks, and modules used for the automated synthesis as well as postassembly manipulations can be found in the Supporting Information.

### Temperature Regulation System

A Discover microwave reactor (CEM) houses the reaction vessel. A jacket surrounding the reaction vessel provides constant cooling to the lowest target temperature during the synthesis (>−40 °C). The cooling jacket is in fluid communication with a chiller that circulates a microwave transparent coolant working at a constant temperature. Any higher temperature during the synthesis cycle (up to 100 °C) is reached by microwave radiation. The reagent temperature is continuously monitored with an fiber-optic probe inside the reaction vessel. The maximum microwave power used depends on the reagents and is dynamically adjusted. The solvents, building block, and activator solutions are cooled to −8 °C before reaching the reaction vessel by a precooling unit (see Supporting Information). The reaction vessel holds the solid support. Gas and liquid flow in and out of the reaction vessel via the top and bottom inlets/outlets. A top outlet vents the exhaust gas.

### Delivery Systems

The reagent containers are categorized into solvents, building blocks, activators, capping, and deprotection/functionalization. Each group has a separate pressurized inert atmosphere provided by a gas manifold. A syringe pump drives the building block and activator solutions from the reservoir to the upper part of the reaction vessel. Both reagents travel through separate handles by rotary valves. A buffering volume line between the pump and the reservoirs prevents the reagents from mixing. A third top inlet dispenses the solvents for washing and the gas for draining the reactor vessel. A top outlet vents the exhaust gas. The bottom inlet/outlet connects multiport valve. It serves to drain the liquid, delivering bubbling gas for mixing and postcoupling reagents (deprotection, capping, or postmodification reagent solutions). The washing solvents and bottom supplied reagents are gas-driven by differential pressure. The vent gases and drained liquid go to the waste container. Alternatively, the drained solutions can be collected for analysis or recovery of unreacted components.

### Modular Design

A computer centralizes the control of delivery and temperature regulation systems. The entire device has a modular construction. All of the components are accessible and replaceable. Capabilities can be expanded by adding elements or reorganizing the fluid pathway. In a single working environment, the software allows the creation and storage of operational modules listing a series of ground-level commands (on, off the device, and setting parameters). The modules execute generic process tasks such as system initialization, reactions, and the standby operation. The user builds a synthesis program by compiling modules. The settings of each module are adjustable.

### AGA Cycle Modules

This section describes the key modules during an AGA-MW cycle; for further modules, refer to the Supporting Information.

#### Glycosylation

Once the temperature of the reaction vessel has adjusted to the desired temperature of the subsequent glycosylation by the cooling device, 1 mL of the acid wash solution is delivered to the reaction vessel. After 3 min, the solution is drained. Finally, the resin is washed with 3 mL of CH_2_Cl_2_. Upon draining the reaction vessel, 1 mL of building block solution containing the appropriate building block is delivered from the building block storing component to the reaction vessel through the precooling device (set at −20 °C). After the temperature again reaches the desired temperature (*T*_1_), 1 mL of appropriate activator solution (see Supporting Information for specific cases) is delivered to the reaction vessel from the respective activator storing component to the reaction vessel through the precooling device (set at −20 °C). The glycosylation mixture is incubated for the selected duration (*t*_1_) at the desired *T*_1_, then by microwave irradiation (max power = 120 W), the reaction temperature is linearly ramped to *T*_2_ (rate = 4 °C/min). Once *T*_2_ is reached, it is maintained by microwave irradiation and the reaction mixture is incubated for an additional time (*t*_2_). Once the incubation time is finished, the reaction mixture is drained and the resin is washed with CH_2_Cl_2_ (1 × 2 mL for 15 s), then dioxane (1 × 2 mL for 15 s), and finally CH_2_Cl_2_ (2 × 2 mL for 15 s). During the module, the active cooling element is maintained at the lowest temperature required throughout the synthesis.

#### Capping

The resin is washed with DMF (2 × 3 mL for 15 s). Then 2 mL of precapping solution (10% v/v pyridine in DMF) is delivered, and under microwave irradiation, the reaction temperature is adjusted to and maintained at 50 °C for 1 min (max power = 5 W). The resin is then washed with CH_2_Cl_2_ (3 × 2 mL for 15 s). Upon washing, 4 mL of capping solution (20% Ac_2_O, 4% MsOH in CH_2_Cl_2_) is then delivered and the temperature is adjusted and maintained at 25 °C by microwave irradiation (max power = 100 W). The resin and the reagents are incubated for 8 min. The solution is then drained from the reactor vessel, and the resin is washed with CH_2_Cl_2_ (3 × 3 mL for 15 s). During the entire module, the active cooling element is maintained at the lowest temperature required throughout the synthesis.

#### Fmoc Deprotection

The resin is first washed with DMF (3 × 3 mL for 15 s), and then 2 mL of Fmoc deprotection solution (20% v/v of piperidine or 20% v/v Et_3_N in DMF; see Supporting Information for specific cases) is delivered to the reaction vessel. The temperature of the reagents inside the reactor vessel is then adjusted to and maintained at 60 °C by microwave irradiation (max power = 60 W). After 1 min, the reaction solution is drained and the resin is washed with DMF (3 × 3 mL for 15 s) and CH_2_Cl_2_ (5 × 3 mL for 15 s). During the module, the active cooling element is maintained at the lowest temperature required throughout the synthesis. After this module, the resin is ready for the next glycosylation cycle.

#### Lev Deprotection

The resin is washed with CH_2_Cl_2_ (3 × 2 mL for 15 s), and then 2 mL of Lev deprotection solution, and N_2_H_4_·HOAc in CH_2_Cl_2_/Pyr/HOAc/H_2_O (20:16:4:1) is delivered to the reaction vessel. The temperature of the reagents inside the reactor vessel is then adjusted to and maintained at 35 °C by microwave irradiation (max power = 180 W). After 5 min, the reaction solution is drained from the reactor vessel and the resin is washed with CH_2_Cl_2_ (3 × 2 mL for 15 s). Then, fresh Lev deprotection solution (2 mL) is delivered, and the process is repeated twice more. Then, the resin is washed with DMF, THF, and CH_2_Cl_2_ (3 × 3 mL for 15 s). During the module, the active cooling element is maintained at the lowest temperature required throughout the synthesis. After this module, the resin is ready for the next glycosylation cycle.

#### NAP Deprotection

The resin is first washed with CH_2_Cl_2_ (3 × 2 mL for 15 s), and then 2 mL of NAP deprotection solution (0.022 m/v DDQ in a 4:1 mixture of DCE/methanol) was delivered to the reaction vessel. The temperature of the reagents inside the reactor vessel is then adjusted to and maintained at 60 °C by microwave irradiation (max power = 180 W). After 30 min, the reaction solution is drained from the reactor vessel. The resin is washed with CH_2_Cl_2_ (3 × 2 mL for 15 s). Then, fresh NAPdeprotection solution (2 mL) is delivered, and the process is repeated twice more. Then, the resin is washed with DMF, THF, and CH_2_Cl_2_ (3 × 3 mL for 120 s). During the module, the active cooling element is maintained at the lowest temperature required throughout the synthesis. After this module, the resin is ready for the next glycosylation cycle.

#### ClAc Deprotection

The resin is first washed with CH_2_Cl_2_ (3 × 2 mL for 15 s), and then ClAc deprotection solution (2 mL, 5% w/w thiourea in a 10:1 mixture of 2-methoxyethanol/pyridine) was delivered to the reaction vessel. The temperature of the reagents inside the reactor vessel is then adjusted to and maintained at 90 °C by microwave irradiation (max power = 180 W). After 22 min, the reaction solution is drained from the reactor vessel. The resin is washed with DMF (3 × 2 mL for 15 s). Then fresh ClAc deprotection solution (2 mL) is delivered, and the process is repeated twice more. Then, the resin is washed with DMF (3 × 3 mL for 15 s) and CH_2_Cl_2_ (5 × 3 mL for 15 s). During the module, the active cooling element is maintained at the lowest temperature required throughout the synthesis. After this module, the resin is ready for the next glycosylation cycle.

### Glycan Modification

Sulfation serves as an example for a postassembly modification reaction. The resin is first washed with CH_2_Cl_2_ (3 × 2 mL for 15 s), and then 2 mL of sulfation solution, SO_3_·TMA (20 equiv/OH) in DMF, was delivered to the reaction vessel. The temperature of the reagents inside the reactor vessel is then adjusted to and maintained at 90 °C by microwave irradiation (max power = 90 W). After 15 min, the reaction solution is drained from the reactor vessel. Again, 2 mL of fresh sulfation solution is added, and the temperature is adjusted and maintained at 90 °C by microwave irradiation for 15 min (max power = 90 W). Upon completion, the resin is washed with DMF (3 × 2 mL for 15 s).

Methanolysis on resin was accomplished by washing the resin with CH_2_Cl_2_ (3 × 2 mL for 15 s), and 2 mL of methanolyis solution (1:9 dissolution of sodium methoxide in methanol (0.5 M) in THF) is delivered to the reaction vessel at room temperature. After 1 h, the reaction solution is drained from the reactor vessel. The incubation in methanolysis solution was repeated three more times. Then, the resin is washed with 10% aqueous citric acid, DMF, THF, and CH_2_Cl_2_ (3 × 3 mL for 120 s).

## Supporting Information Available

Additional figures, detailed materials and conditions for AGA; microwave-assisted AGA system (DTRR system) construction; oligosaccharide characterization, including HPLC chromatograms, mass, and NMR spectra; preparation conditions of the orthogonal building block **7** (PDF)
