# Supplementary material for: Microwave-Assisted Automated Glycan Assembly
Source: J Am Chem Soc. 2021 Jun 1;143(23):8893–901. doi: 10.1021/jacs.1c03851 (PMC8213053; doi:10.1021/jacs.1c03851)
Supplement: Supplementary file 1 — ja1c03851_si_001.pdf [file ja1c03851_si_001.pdf]

Supporting Information

Microwave-assisted Automated Glycan Assembly

José Danglad-Flores[*†], Sabrina Leichnitz[*†‡], Eric T. Sletten[*†], A. Abragam Joseph[†], Klaus Bienert[†], Kim Le Mai Hoang[†§], Peter. H. Seeberger[†‡]

[†]Department of Biomolecular Systems, Max-Planck-Institute of Colloids and Interfaces, Am Mühlenberg 1, 14476 Potsdam, Germany
[‡]Institute of Chemistry and Biochemistry, Freie Universität Berlin, Arnimallee 22, 14195 Berlin, Germany
§GlycoUniverse GmbH & Co KGaA, Am Mühlenberg 11, 14476 Potsdam, Germany

AUTHOR INFORMATION

Corresponding author: Prof. Dr. Peter H. Seeberger

E-mail: peter.seeberger@mpikg.mpg.de

Table of Contents

1 Additional Figures 3
2 General Information 4
3 Materials and Conditions for Automated Synthesis 5
3.1 Materials and Measurements 5
3.2 Preparation of Stock Solutions 5
3.3 Modules for Automated Synthesis 7
3.4 Post-synthesizer Manipulation 10
3.5 Analytical NP/RP-HPLC and purification 12
4 Microwave-assisted Automated Glycan Assembly System (DTRR System) 13
4.1 Dual Temperature Regulation Reaction System 13
4.2 Reagents storage 13
4.3 Delivery Systems 14
4.4 Automated Control System 14
5 Automated Glycan Assembly of Oligosaccharides 15
5.1 Glucose Octamer 10 15
5.2 Lewis Antigen 11 19
5.3 Mannose Octamer 12 23
5.4 Keratan Sulfate 13 27
5.5 Automated Glycan Assembly Using an Orthogonal Building Block 32
5.5.1 Linear Mannose Tetramers (14-17) 32
5.5.2 Non-Linear On-Resin Deprotected Trimer (18) 48
5.5.3 Double Branched Trimer (S2) 54
5.5.4 Bisecting GlcNAc Tetramer (Triple-Branched Mannose 19) 58
5.5.5 Hyperbranched Mannose Pentamer (20) 62
6 Preparation of Orthogonal Building Block 7 67

1 Additional Figures

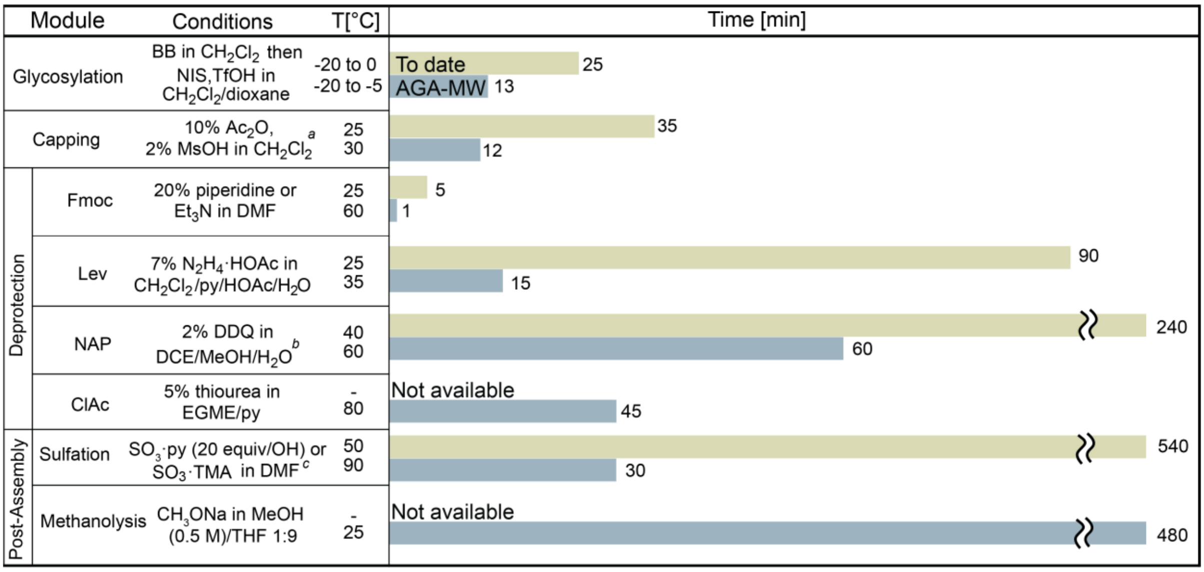

[a]AGA-MW: double concentration. [b]AGA-MW: only DCE and MeOH. [c]Traditional AGA: SO_3·py in py/DMF, AGA-MW:SO_3·TMA in DMF.

Figure S-1. Comparison of modules in the standard system (without microwave) and the DTRR system (with microwave) and overview over the modules and conditions.

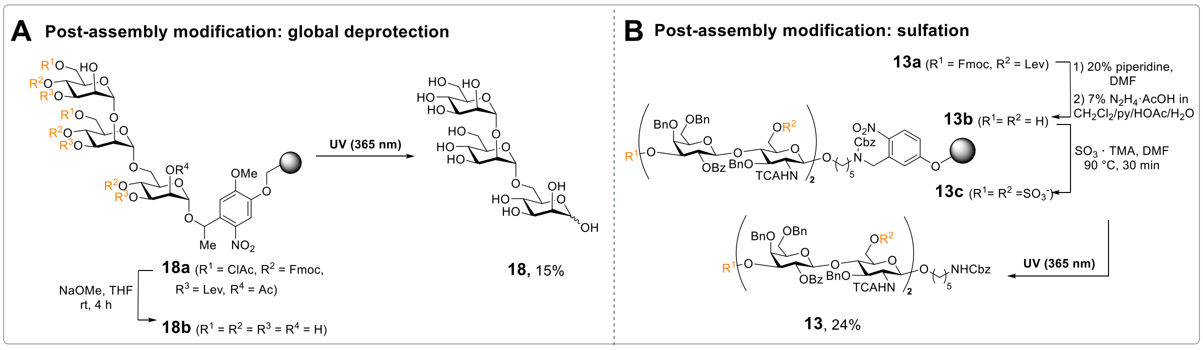

Figure S-1. Post-assembly modifications achieved by the DTRR system. (A) Trisaccharide **18** was assembled from building block **7** and fully deprotected on-resin. (B) Synthesis and on-resin sulfation of tetrasaccharide **13**.

2 General Information

All **chemicals** were reagent grade and used as supplied unless otherwise noted. All **solvents** for chemical reactions were commercially purchased in p.a. quality. If stated, they were dried in a Solvent Dispensing System (J.C. Meyer). For HPLC and MS spectrometry, solvents with corresponding quality were used. Water was used from a Milli Q-station from Millipore. **The automated syntheses** were performed on a TempDUO home-built synthesizer developed at the Max Planck Institute of Colloids and Interfaces described in section 4.

Reaction completion, identity, and purity of all compounds were determined by low resolution mass spectrometry (**ESI-LRMS**) or analytical thin-layer chromatography (**TLC**). TLC was performed on Merck silica gel 60 F_{254} plates (0.25 mm). Compounds were visualized by UV irradiation (254 nm) or stained (*p*-Anisaldehyde Stain: 3.7 mL *p*-anisaldehyde, 135 mL ethanol, 5 mL sulfuric acid, 1.5 mL glacial acetic acid or Hanessian's Stain: 235 mL of distilled water, 12 g of ammonium molybdate, 0.5 g of ceric ammonium molybdate, and 15 mL sulfuric acid). **Flash column chromatography** was performed on Kieselgel 60 with 230-400 mesh (Sigma-Aldrich, St. Louis, USA) or C_{18}-reverse phased silica gel, fully endcapped (Sigma-Aldrich, St. Louis, USA). Analysis and purification by normal and reverse phase **HPLC** and ESI-LRMS was performed by using an Agilent 1200 series. Products were **lyophilized** using a Christ Alpha 2-4 LD plus freeze dryer. ^{1}H, ^{13}C, COSY and HSQC **NMR spectra** were recorded in parts per million (δ) relative to the resonance of the solvent on a Varian 400-MR (400 MHz), Varian 600-MR (600 MHz), or Bruker Biospin AVANCE700 (700 MHz) spectrometer. Assignments were supported by COSY and HSQC experiments. High resolution mass spectra (**HRMS**) were obtained using 6210 ESI-TOF mass spectrometer (Agilent) and **MALDI-TOF** autoflexTM (Bruker) instruments. **IR spectra** were recorded on a Perkin-Elmer 1600 FTIR spectrometer. **Optical rotations** were measured by using a Perkin-Elmer 241 and Unipol L1000 polarimeter, with concentrations expressed in g/100 mL.

Amberlite IR-120 (Across Organics) protonic exchange resin was rinsed with THF, water, methanol and dichloromethane before use. Palladium on carbon was removed from reaction mixtures by filtration with Rotilabo syringe filters (Roth), PTFE filters (pore size: 0.45 μm).

3 Materials and Conditions for Automated Synthesis

3.1 Materials and Measurements

Solvents used for dissolving all building blocks and making of various solutions were taken from Solvent Dispensing System (J.C. Meyer). Wash solvents were HPLC grade. The building blocks were purchased from GlycoUniverse GmbH & CO KGaA or synthesized if stated. Prior to automated synthesis, the building blocks were weighed and co-evaporated three times with anhydrous toluene and dried for at least 1 hour under high vacuum prior to use. All solutions were freshly prepared and kept under argon during the automation process. Isolated yields of products were calculated on the basis of resin loading. Functionalized resin **8** and **9** were synthesized as previously reported[1] and resin loading (0.3 mmol/g for **9** and 0.4 mmol/g for **8**) was determined following a published protocol.[2] Resin was placed in the reaction vessel and was swollen in dichloromethane for 20 min at room temperature before starting the first module. During this time, all reagent lines involved in the synthesis were washed and primed.

3.2 Preparation of Stock Solutions

Building Block Solution 1 (for thioglycosides): Thioglycoside building block (0.09 mmol, 6.5 equiv. per cycle) was dissolved in 1 mL (per cycle) of anhydrous CH_2Cl_2.

Building Block Solution 2 (for glycosyl phosphates): Glycosyl phosphate building block (0.06 mmol, 4.7 equiv. per cycle) was dissolved in 1 mL (per cycle) of anhydrous CH_2Cl_2.

Acidic Wash Solution: TMSOTf (0.45 mL, 2.5 mmol) was added to 40 mL of anhydrous CH_2Cl_2.

Activator Solution 1 (for thioglycosides): Recrystallized NIS (1.58 g, 7.02 mmol) was dissolved in 45 mL of a 2:1 mixture of anhydrous CH_2Cl_2/dioxane, followed by addition of triflic acid (55 µL, 0.6 mmol). The solution was kept under ice-bath cooling for the duration of the automated run.

Activator Solution 2 (for glycosyl phosphates): TMSOTf (0.9 mL, 5.0 mmol) was added to 40 mL of anhydrous CH_2Cl_2.

Pre-capping Solution: Pyridine (10 mL) was added to 90 mL of DMF.

Capping Solution: Methanesulfonic acid (1.2 mL, 18.5 mmol), acetic anhydride (6 mL, 63.5 mmol) were added to 50 mL of anhydrous CH_2Cl_2.

Concentrated Capping Solution: Methanesulfonic acid (2.4 mL, 37 mmol), acetic anhydride (12 mL, 127 mmol) were added to 50 mL anhydrous CH_2Cl_2.

Lev Deprotection Solution: $N_2H_4 \cdot HOAc$ (725 mg, 7.87 mmol) was dissolved in 50 mL of a 4:1:0.25 mixture of pyridine/acetic acid/water.

Fmoc Deprotection Solution 1: Piperidine (20 mL) was added to 80 mL anhydrous DMF.

Fmoc Deprotection Solution 2: Et_3N (20 mL) was added to 80 mL anhydrous DMF.

NAP Deprotection Solution: DDQ (910 mg, 4.00 mmol) was dissolved in 40 mL of a 4:1 mixture of DCE/methanol. The solution was protected from light by aluminium foil for the duration of the automated run.

ClAc Deprotection Solution: Thiourea (2.5 g, 32.84 mmol) was dissolved in 55 mL of a 10:1 mixture of 2-methoxyethanol/pyridine.

Sulfation Solution: $SO_3 \cdot TMA$ (900 mg, 6.5 mmol) was added to 16 mL of DMF and sonicated until dissolved.

Methanolysis Solution: A solution of sodium methoxide in methanol (1 mL, 0.5 M) was added to 9 mL THF.

3.3 Modules for Automated Synthesis

Initiation: The resin loaded in the reaction vessel is washed with DMF, THF, and CH_2Cl_2 (3 x 3 mL for 15 s, respectively). The resin is then swollen in 2 mL CH_2Cl_2 for 20 minutes while the temperature of the reaction vessel is cooled to the lowest temperature required throughout the synthesis.

Module I - Acidic Washing: Once the temperature of the reaction vessel has adjusted to the desired temperature of the subsequent glycosylation by the cooling device, 1 mL of the **Acidic Wash Solution** is delivered to the reaction vessel through the precooling device (set at -20 °C). After three minutes, the solution is drained. Finally, the resin is washed with 3 mL CH_2Cl_2 (bubbling = 15 s) and drained.

Module IIa – Glycosylation (for thioglycosides): Upon draining the CH_2Cl_2 in the reaction vessel, 1 mL of **Building Block Solution 1** containing the appropriate building block is delivered from the building block storing component to the reaction vessel through the precooling device (set at -20 °C). After the temperature reaches the desired temperature (T_1), **Activator Solution 1** (1 mL) is delivered to the reaction vessel from the respective activator storing component to the reaction vessel through the precooling device (set at -20 °C). The glycosylation mixture is incubated for the selected duration (t_1) at the desired T_1, then by microwave irradiation (max power = 120 W) the reaction temperature is linearly ramped to T_2 (rate = 4 °C/min). Once T_2 is reached, it is maintained by microwave irradiation and the reaction mixture is incubated for an additional time (t_2). Once the incubation time is finished, the reaction mixture is drained and the resin is washed with CH_2Cl_2 (1 x 2 mL for 15 s), then dioxane (1 x 2 mL for 15 s), and finally CH_2Cl_2 (2 x 2 mL for 15 s). During the module, the active cooling element is maintained at the lowest temperature required throughout the synthesis.

Module IIb – Glycosylation (for glycosyl phosphate): Upon draining the CH_2Cl_2 in the reaction vessel, **Building Block Solution 1** (1 mL) containing the appropriate building block is delivered from the building block storing component to the reaction vessel through the precooling device (set at -20 °C). After the temperature again reaches the desired temperature (T_1), **Activator Solution 1** (1 mL) is delivered to the reaction vessel from the respective activator storing component to the reaction vessel through the precooling device (set at -20 °C). The glycosylation mixture is incubated for the selected duration (t_1) at the desired T_1, then by microwave irradiation (max power = 180 W) the reaction temperature is linearly ramped to T_2 (rate = 4 °C/min). Once T_2 is reached, it is maintained by microwave irradiation and the reaction mixture is incubated for an additional time (t_2). Once the incubation time is finished, the reaction mixture is drained and the resin is washed with DCE (1 x 2 mL for 5 s). During the module, the active cooling element is maintained at the lowest temperature required throughout the synthesis.

Module IIIa - Capping: The resin is washed with DMF (2 x 3 mL for 15 s). Then **Pre-capping Solution** (2 mL) is delivered and under microwave irradiation the reaction temperature is adjusted to and maintained at 50 °C for one minute (max power = 5 W). The resin is then washed with CH_2Cl_2 (3 x 2 mL for 15 s). Upon washing, **Capping Solution** (4 mL) is delivered and the temperature is adjusted and maintained 25 °C by microwave irradiation (max power = 100 W). The resin and the reagents are incubated for 8 min. The solution is then drained from the reactor vessel and the resin is washed with CH_2Cl_2 (3 x 3 mL for 15 s). During the entire module, the active cooling element is maintained at the lowest temperature required throughout the synthesis.

Module IIIb – Capping (Sterically hindered hydroxyls): The resin is initially washed with DMF (2 x 3 mL for 15 s). Then **Pre-capping Solution** (2 mL) is delivered and under microwave irradiation the reaction temperature is adjusted to and maintained at 50 °C for one minute (max power = 5 W). The resin is then washed with CH_2Cl_2 (3 x 2 mL for 15 s). Upon washing, of **Concentrated Capping Solution** (4 mL) is then delivered and the temperature is adjusted to and maintained at 25 °C by microwave irradiation (max power = 100 W). The resin and the reagents are incubated for 8 min. The solution is then drained from the reactor vessel and the resin is washed with CH_2Cl_2 (2 x 3 mL for 15 s). Then, DMF (4 mL) is delivered and irradiated with microwaves (max power = 5 W) for 10 s and the solution is allowed to incubate for an additional 50 s before draining. During the module, the active cooling element is maintained at the lowest temperature required throughout the synthesis.

Module IVa - Fmoc Deprotection 1: The resin is first washed with DMF (3 x 3 mL for 15 s), and then **Fmoc Deprotection Solution 1** (2 mL) is delivered to the reaction vessel. The temperature of the reagents inside the reactor vessel is then adjusted to and maintained at 60 °C by microwave irradiation (max power = 60 W). After 1 min the reaction solution is drained and the resin is washed with DMF (3 x 3 mL for 15 s) and CH_2Cl_2 (5 x 3 mL for 15 s). During the module, the active cooling element is maintained at the lowest temperature required throughout the synthesis. After this module the resin is ready for the next glycosylation cycle.

Module IVb - Lev Deprotection: The resin is washed with CH_2Cl_2 (3 x 2 mL for 15 s), and then **Lev Deprotection Solution** (2 mL) is delivered to the reaction vessel. The temperature of the reagents inside the reactor vessel is then adjusted to and maintained at 35 °C by microwave irradiation (max power = 180 W). After 5 min, the reaction solution is drained from the reactor vessel and the resin is washed with CH_2Cl_2 (3 x 2 mL for 15 s). Then, of fresh **Lev Deprotection Solution** (2 mL) is delivered and the process is repeated twice more. Then, the resin is washed with DMF, THF, and CH_2Cl_2 (3 x 3 mL for 15 s, respectively). During the module, the active cooling element is maintained at the lowest temperature required throughout the synthesis. After this module the resin is ready for the next glycosylation cycle.

Module IVc - Fmoc Deprotection 2: The resin is first washed with DMF (3 x 3 mL for 15 s), and then **Fmoc Deprotection Solution 2** (2 mL) is delivered to the reaction vessel. The temperature of the reagents inside the reactor vessel is then adjusted to and maintained at 60 °C by microwave irradiation (max power = 60 W). After 5 min the reaction solution is drained and the resin is washed with DMF (3 x 2 mL for 15 s). Then, fresh **Fmoc Deprotection Solution 2** (2 mL) is delivered and the process is repeated twice more. Then, the resin is washed with DMF (3 x 3 mL) and CH_2Cl_2 (3 x 3 mL) for 15 s each time. During the module, the active cooling element is maintained at the lowest temperature required throughout the synthesis. After this module the resin is ready for the next glycosylation cycle.

Module IVd – NAP Deprotection: The resin is first washed with CH_2Cl_2 (3 x 2 mL for 15 s) then **NAP Deprotection Solution** (2 mL) was delivered to the reaction vessel. The temperature of the reagents inside the reactor vessel is then adjusted to and maintained at 60 ºC by microwave irradiation (max power = 180 W). After 30 min, the reaction solution is drained from the reactor vessel. The resin is washed with CH_2Cl_2 (3 x 2 mL for 15 s). Then, fresh **NAP Deprotection Solution** (2 mL) is delivered and the process is repeated twice more. Then, the resin is washed with DMF, THF, and CH_2Cl_2 (3 x 3 mL for 120 s, respectively). During the module, the active cooling element is maintained at the lowest temperature required throughout the synthesis. After this module the resin is ready for the next glycosylation cycle.

Module IVe – ClAc Deprotection: The resin is first washed with CH_2Cl_2 (3 x 2 mL for 15 s) then **ClAc Deprotection Solution** (2 mL) was delivered to the reaction vessel. The temperature of the reagents inside the reactor vessel is then adjusted to and maintained at 90 °C by microwave irradiation (max power = 180 W). After 22 min, the reaction solution is drained from the reactor vessel. The resin is washed with DMF (3 x 2 mL for 15 s). Then fresh **ClAc Deprotection Solution 2** (2 mL) is delivered and the process is repeated twice more. Then, the resin is washed with DMF (3 x 3 mL for 15 s) and CH_2Cl_2 (5 x 3 mL for 15 s). During the module, the active cooling element is maintained at the lowest temperature required throughout the synthesis. After this module the resin is ready for the next glycosylation cycle.

Module V – Sulfation: The resin is first washed with CH_2Cl_2 (3 x 2 mL for 15 s) then **Sulfation Solution** (2 mL) was delivered to the reaction vessel. The temperature of the reagents inside the reactor vessel is then adjusted to and maintained at 90 ºC by microwave irradiation (max power = 90 W). After 15 min the reaction solution is drained from the reactor vessel. Again, fresh **Sulfation Solution** (2 mL) is added and the temperature is adjusted and maintained at 90 ºC by microwave irradiation for 15 min (90 W). Upon completion, the resin is washed with DMF (3 x 2 mL for 15 s).

Module VI – Methanolysis: The resin was washed with CH_2Cl_2 (3 x 2 mL for 15 s) then **Methanolyis Solution** (2 mL) was delivered to the reaction vessel at room temperature. After

1 h the reaction solution is drained from the reactor vessel. The incubation in **Methanolysis Solution** was repeated three more times. Then, the resin is washed with 10% aqueous citric acid, DMF, THF, and CH_2Cl_2 (3 x 3 mL for 120 s, respectively).

3.4 Post-synthesizer Manipulation

Cleavage from Solid Support (Method A-1): Protected Oligosaccharides

After automated synthesis, the resin was removed from the reaction vessel, suspended in CH_2Cl_2 (20 mL), and photocleaved in a continuous-flow photoreactor. A Vapourtec E-Series easy-MedCHem, equipped with a UV-150 Photochemical reactor having a UV-150 Medium-Pressure Mercury Lamp (arc length 27.9 cm, 450 W) surrounded by a long-pass UV filter (Pyrex, 50% transmittance at 305 nm) was used. A Pump 11 Elite Series (Harvard Apparatus syringe pump at a flow rate of 0.8 mL/min was used to pump the mixture through a FEP tubing (i.d. 3.0 inch, volume: 12 mL) at 20 °C. The reactor was washed with 20 mL CH_2Cl_2 at a flow rate of 2.0 mL/min. The output solution was filtered to remove the resin and the solvent was evaporated *in vacuo*. Crude was then analyzed by MALDI.

Cleavage from Solid Support (Method A-2): Deprotected Oligosaccharides

After automated synthesis, the resin was removed from the reaction vessel, suspended in a 10:1 mixture of THF/water (20 mL), and photocleaved in a continuous-flow photoreactor. A Vapourtec E-Series easy-MedCHem, equipped with a UV-150 Photochemical reactor having a UV-150 LED lamp (365 nm) was used. A Pump 11 Elite Series (Harvard Apparatus syringe pump at a flow rate of 2 mL/min was used to pump the mixture through a FEP tubing (i.d. 3.0 inch, volume: 12 mL) at 20 °C. The reactor was washed with 20 mL of a 10:1 mixture of THF/water at a flow rate of 2.0 mL/min, followed by water, acetonitrile and CH_2Cl_2. The output solution was filtered to remove the resin and the solvent was evaporated *in vacuo*. Crude was then analyzed by MALDI.

Cleavage from Solid Support (Method A-3): Sulfated Oligosaccharides

After automated synthesis, the resin was removed from the reaction vessel, suspended in a 9:1 mixture of CH_2Cl_2/MeOH (20 mL) and photocleaved in a continuous-flow photoreactor. A Vapourtec E-Series easy-MedCHem, equipped with a UV-150 Photochemical reactor having a UV-150 Medium-Pressure Mercury Lamp (arc length 27.9 cm, 450 W) surrounded by a long-pass UV filter (Pyrex, 50% transmittance at 305 nm) was used. A Pump 11 Elite Series (Harvard Apparatus syringe pump at a flow rate of 0.7 mL/min was used to pump the mixture through a FEP tubing (i.d. 3.0 inch, volume: 12 mL) at 20 °C. An addition 20 mL of a 9:1 mixture of CH_2Cl_2/MeOH at a flow rate of 0.7 mL/min was then passed through the reactor. The reactor

was then washed with CH_2Cl_2 at rate of 2.0 mL/min. The output solution was filtered to remove the resin and the solvent was evaporated *in vacuo*. Crude was then analyzed by QToF (negative mode).

Deprotection of Oligosaccharides

AGA-synthesized and photocleaved product was subjected to methanolysis and hydrogenolysis. The hydrogenolysis product was purified by RP-HPLC and lyophilized on a Christ Alpha 2-4 LD plus freeze dryer to afford the final deprotected compound.

- **Methanolysis (Method C):** To a solution of protected oligosaccharide in MeOH:CH_2Cl_2 (2 mL, 1:1), sodium methoxide (0.5 M solution in MeOH, 2.2 equiv. per ester group) was added. The mixture was stirred at room temperature for 2 h. Then Amberlite IR-120 (H^+ form) was added to quench. After neutralization, the reaction mixture was filtered and the solvent was removed *in vacuo*. The crude compound was used for hydrogenolysis without further purification.
- **Hydrogenolysis (Method D):** The crude compound obtained after methanolysis was dissolved in 4 mL of EtOAc:*t*-BuOH:H_2O (2:1:1). Pd/C (10%) was added to the solution and the suspension was stirred in a H_2 bomb with 60 psi pressure over night. The insoluble material was removed by a CHROMAFIL ®Xtra, RC 0.45 syringe filter. The solid was washed once with *t*-BuOH and several times with water. The filtrate was collected and concentrated *in vacuo*.

3.5 Analytical NP/RP-HPLC and purification

Analytical NP-HPLC of Crude Material (Method B-1)

Analytical NP-HPLC was conducted on an Agilent 1200 Series system. A YMC-Diol-300-NP column (150 mm x 4.60 mm I.D.) was used with a flow rate of 1.00 mL/min and hexane/EtOAc as eluent (20% EtOAc in hexane for 5 min, 20 → 100% EtOAc in hexane over 35 min, 100% EtOAc for 10 min).

Analytical NP-HPLC of Crude Material (Method B-2)

Analytical NP-HPLC was conducted on an Agilent 1200 Series system. A YMC-Diol-300-NP column (150 mm x 4.60 mm I.D.) was used at a flow rate of 1.00 mL/min with hexane/EtOAc as eluent (20% EtOAc in hexane for 5 min, 20 → 55% EtOAc in hexane over 35 min, 55 → 100% EtOAc in hexane over 35 min, 100% EtOAc for 10 min).

Preparative NP-HPLC of Crude Material (Method B-3)

Analytical NP-HPLC was conducted on an Agilent 1200 Series system. A YMC-Diol-300-NP column (150 mm x 20 mm I.D.) was used at a flow rate of 15.00 mL/min with hexane/EtOAc as eluent (20% EtOAc in hexane for 5 min, 20 → 55% EtOAc in hexane over 35 min, 100% EtOAc for 10 min).

Preparative NP-HPLC of Crude Material (Method B-4)

Analytical NP-HPLC was conducted on an Agilent 1200 Series system. A YMC-Diol-300-NP column (150 mm x 20 mm I.D.) was used at a flow rate of 15.00 mL/min with hexane/EtOAc as eluent (20% EtOAc in hexane for 5 min, 20 → 100% EtOAc in hexane over 35 min, 100% EtOAc for 10 min).

Analytical/preparative RP-HPLC of Crude Material (Method E-1)

Crude products were dissolved in water and analyzed/purified using analytical/preparative HPLC. A Thermo-Scientific Hypercarb column (150 mm x 4.60 mm I.D.) was used for analytical RP-HPLC with a flow rate of 0.70 mL/min with water (0.1% HCO_2H)/acetonitrile as eluents (100% H_2O (0.1% HCO_2H) for 5 min, 0 → 30% acetonitrile in H_2O (0.1% HCO_2H) over 30 min, 30 → 100% acetonitrile in H_2O (0.1% HCO_2H) over 5 min, 100% acetonitrile for 5 min).

Analytical/preparative RP-HPLC of Crude Material (Method E-2)

Crude products were dissolved in water and analyzed/purified using analytical/preparative HPLC. A Thermo-Scientific Hypercarb column (150 mm x 4.60 mm I.D.) was used for analytical RP-HPLC with a flow rate of 0.70 mL/min with H_2O (0.1% HCO_2H)/acetonitrile as eluents (100% H_2O (0.1% HCO_2H) for 5 min, 0 → 10% acetonitrile in H_2O (0.1% HCO_2H) over 30 min, 10 → 100% acetonitrile in H_2O (0.1% HCO_2H) over 5 min, 100% acetonitrile for 5 min).

Analytical RP-HPLC of Crude Material (Method E-3)

Crude product was dissolved in 50% water/acetonitrile and analyzed using a Synergi column (250 mm x 4.60 mm I.D.) for analytical RP-HPLC with a flow rate of 1.0 mL/min with aqueous 0.01 M NH_4HCO_3/acetonitrile as eluents (95% 0.01 M NH_4HCO_3 for 5 min, 5 → 100% acetonitrile over 30 min, 100% acetonitrile for 5 min).

4 Microwave-assisted Automated Glycan Assembly System (DTRR System)

4.1 Dual Temperature Regulation Reaction System

A microwave reactor Discover from CEM accommodates the reaction vessel. A jacket surrounding the reaction vessel provides constant cooling to the lowest target temperature during the synthesis (up to -40 °C). The cooling jacket is in fluid communication with a Unistat 705w chiller from Huber that circulates a microwave transparent coolant working at a constant temperature. We adjust any higher temperature during the cycle (up to 100 °C) by microwave irradiation. The reagent temperature is continuously monitored with an optic fiber probe in the reaction vessel. While the maximum microwave power irradiated depends on the reagents, and it is dynamically adjusted. The solvents, donor, and activator solutions are cooled to -8 °C before reach the reaction vessel. The cooling power, in this case, is provided by Peltier Elements supported with circulating cooling water at 15 °C, in aluminum constructions thermally isolate.

4.2 Reagents storage

The reagents are separately loaded under argon in glass vessels categorized into solvents, donors, activators, capping, and deprotection/functionalization. Three 2.5 L bottles supply CH_2Cl_2, DMF and THF for washing, one 2.5 L bottle containing DCE as driving liquid for the syringe pump. Up to eight tubes (capacity = 10 mL) can accommodate the building block solutions. Two 100 mL vessels are available for capping reagents. There are positions for three activator reagents (100 mL or optional 250 mL each), with one container placed in an ice bath to preserve sensitive reagents (e.g. NIS/TfOH). Simultaneously, up to four reagents are loaded for deprotection and/or functionalization. Each group of reagents has a separate pressurized inert atmosphere. A tailor-made Swagelok manifold provides argon (grade = 5.0) throughout the system.

4.3 Delivery Systems

A Kloehn V6 syringe pump with 0.05 mL precision delivers the building block and activator solutions from their reservoirs through the top of the reaction vessel. Both reagents travel through separate lines via Kloehn rotary valves, a 12-way (glycosyl donor building block) and a 6-way (activators) valve. A buffering volume line between the pump and the reservoirs prevents the reagents from mixing. A third top inlet is connected to a Bio-Chem 4-way solenoid valve which provides the solvents for washing and the gas for draining the reactor vessel. A top outlet vents the exhaust gas. The bottom inlet/outlet connects to a Bio-Chem 8-way valve. This valve serves to drain the liquid, provides gas for bubbling/mixing, and delivery post-coupling reagents (deprotections, capping, or post glycan synthesis modification reagent solutions). The washing solvents and bottom supplied reagents are gas driven by differential pressure. The vent gases and drained liquid go to a waste container and are controlled by solenoid valves (Biochem). Alternatively, the drained solutions could be collected for analysis or recovery of the unreacted components. The tubing is made of PTEF and the wetted surface on the valves is Teflon.

4.4 Automated Control System

A computer centralizes the control of the system by a National Instruments PCI-6519 Digital I/O Device connected to the delivery and temperature regulation system. The entire device has a modular construction. All components are accessible and replaceable. The capabilities are expandable by adding elements or reorganizing the pathway of the fluids.

In a single working environment, the software allows for the creation and storage of operational modules by listing a series of ground-level commands (on/off of the devices, and setting parameters). The modules execute generic process tasks such as system initialization, reactions, and the standby operation. The user builds a synthesis program by compiling modules. The settings of each module are adjustable. Once saved the synthesis program can could be loaded and run. The temperature at the reaction vessel and the chiller is monitored and registered. A Voltcraft energy check 3000 reads online the power consumption of the chiller.

5 Automated Glycan Assembly of Oligosaccharides

5.1 Glucose Octamer 10

***N*-Benzyloxycarbonyl-5-amino-pentyl 2-*O*-benzoyl-3,6-di-*O*-benzyl-β-D-glucopyranosyl-(1→4)-2-*O*-benzoyl-3,6-di-*O*-benzyl-β-D-glucopyranosyl-(1→4)-2-*O*-benzoyl-3,6-di-*O*-benzyl-β-D-glucopyranosyl-(1→4)-2-*O*-benzoyl-3,6-di-*O*-benzyl-β-D-glucopyranosyl-(1→4)-2-*O*-benzoyl-3,6-di-*O*-benzyl-β-D-glucopyranosyl-(1→4)-2-*O*-benzoyl-3,6-di-*O*-benzyl-β-D-glucopyranosyl-(1→4)-2-*O*-benzoyl-3,6-di-*O*-benzyl-β-D-glucopyranosyl-(1→4)-2-*O*-benzoyl-3,6-di-*O*-benzyl-β-D-glucopyranoside (10)**

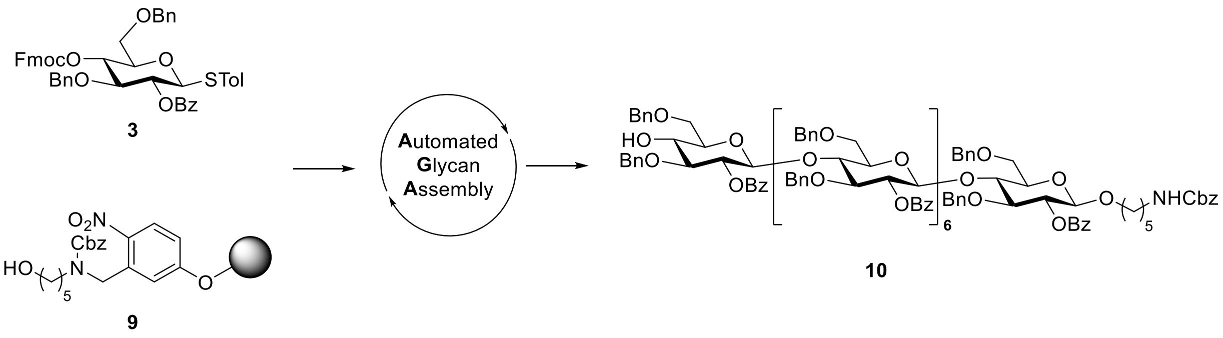

Repeat	Building Blocks	Modules	Notes
**4x**		**I** – Acidic Wash	
	**3** (6.5 equiv.)	**IIb** – Glycosylation with thioglycoside	-20 °C (T_1) 8 min (t_1)<br>-5 °C (T_2) 8 min (t_2)
		**III** – Capping (Conc Capping)<br>**IVa** – Fmoc Deprotection	
**4x**		**I** – Acidic Wash	
	**3** (6.5 equiv.)	**IIb** – Glycosylation with thioglycoside	-20 °C (T_1) 8 min (t_1)<br>-5 °C (T_2) 10 min (t_2)
		**III** – Capping (Conc Capping)<br>**IVa** – Fmoc Deprotection	

Protected **10** was obtained after photocleavage from solid support following **Method A-1**. The crude residue was then purified by normal phase flash chromatography (SiO_2, Hex/EtOAc) or preparative HPLC (**Method B-4**) to afford compound **10** (21 mg, 0.0055 mmol, 41%) as a colorless oil.

^{1}H NMR (700 MHz, $CDCl_3$) δ 7.90 (d, *J* = 7.0 Hz, 2H), 7.87 (d, *J* = 7.4 Hz, 2H), 7.85 – 7.79 (m, 12H), 7.63 – 7.52 (m, 7H), 7.48 (t, *J* = 7.4 Hz, 1H), 7.45 – 7.36 (m, 15H), 7.38 – 7.29 (m, 13H), 7.23 (d, *J* = 7.0 Hz, 2H), 7.18 – 6.84 (m, 54H), 6.99 – 6.84 (m, 19H), 5.23 (dd, *J* = 9.5, 8.1 Hz, 1H), 5.17 (dd, *J* = 9.6, 8.1 Hz, 1H), 5.14 – 5.01 (m, 8H), 4.94 – 4.81 (m, 7H), 4.73 (d, *J* = 11.7

Hz, 1H), 4.67 (d, *J* = 11.6 Hz, 1H), 4.63 (d, *J* = 8.1 Hz, 1H), 4.59 – 4.45 (m, 10H), 4.44 – 4.33 (m, 8H), 4.31 – 4.21 (m, 6H), 4.14 (dd, *J* = 18.7, 12.1 Hz, 2H), 4.08 (t, *J* = 9.3 Hz, 1H), 4.04 – 3.86 (m, 11H), 3.82 – 3.77 (m, 1H), 3.74 – 3.67 (m, 1H), 3.62 – 3.55 (m, 2H), 3.54 – 3.18 (m, 24fH), 3.10 (d, *J* = 9.5 Hz, 1H), 3.04 (d, *J* = 1.9 Hz, 1H), 2.90 – 2.71 (m, 8H), 1.44 – 1.37 (m, 4H), 1.13 – 1.01 (m, 2H) ppm.

^{13}C NMR (176 MHz, $CDCl_3$): δ 165.2, 165.1, 164.9, 156.4, 139.0, 138.9, 138.8, 138.3, 138.3, 137.9, 137.8, 137.7, 136.9, 133.5, 133.3, 133.0, 129.8, 128.6, 128.5, 128.5, 128.4, 128.4, 128.3, 128.3, 128.2, 128.2, 128.1, 128.0, 128.0, 127.9, 127.8, 127.7, 101.3, 100.2, 100.2, 100.0, 82.0, 80.2, 80.1, 76.6, 76.3, 76.2, 76.0, 74.8, 74.7, 74.6, 74.5, 74.4, 74.3, 74.2, 73.9, 73.7, 73.6, 73.4, 73.2, 71.3, 69.5, 67.6, 67.5, 67.3, 66.6, 40.9, 32.1, 29.8, 29.4, 28.9, 23.2, 22.8, 14.3 ppm.

HRMS (QToF): Calcd for $C_{229}H_{227}NO_{51}Na_2$ $[M + 2Na]^{2+}$ 1926.2492; found 1926.2581.

NP-HPLC of crude **10** (ELSD trace, **Method B-3**, t_R = 36.6 min):

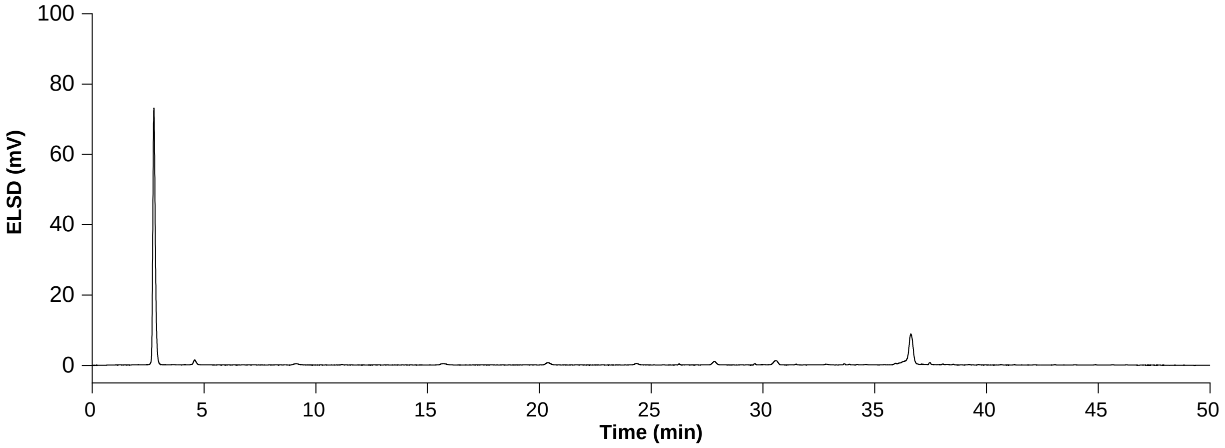

NP-HPLC of purified **10** (ELSD trace, **Method B-3**, t_R = 36.7 min):

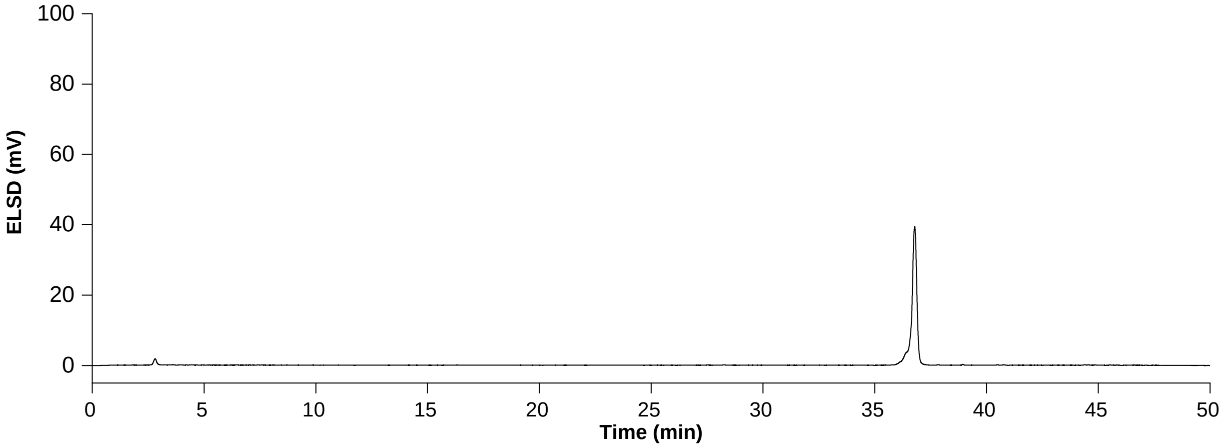

Temperature profile inside the reaction vessel during the synthesis of **10**:

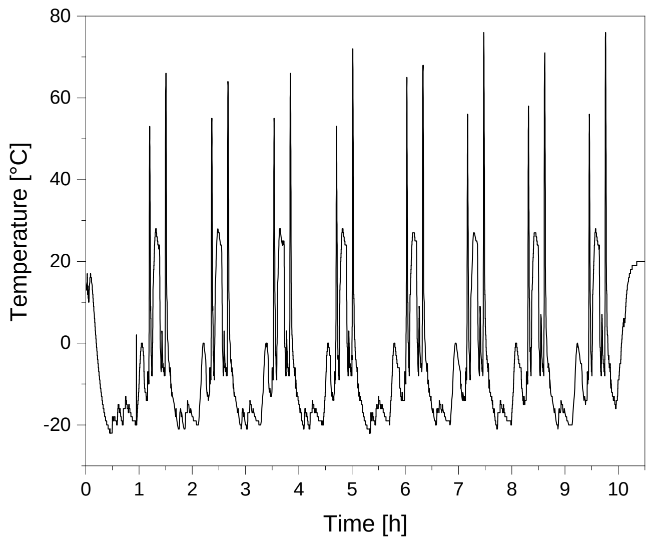

^{1}H NMR (700 MHz, $CDCl_3$) of **10**:

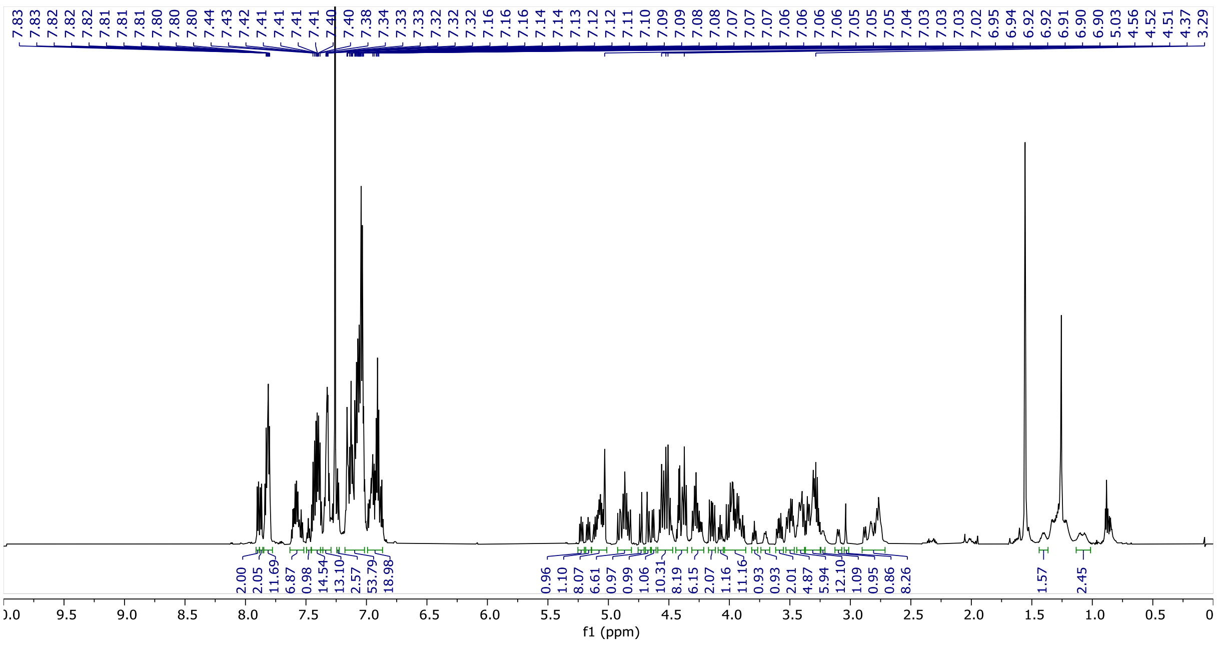

^{13}C NMR (176 MHz, $CDCl_3$) of **10**:

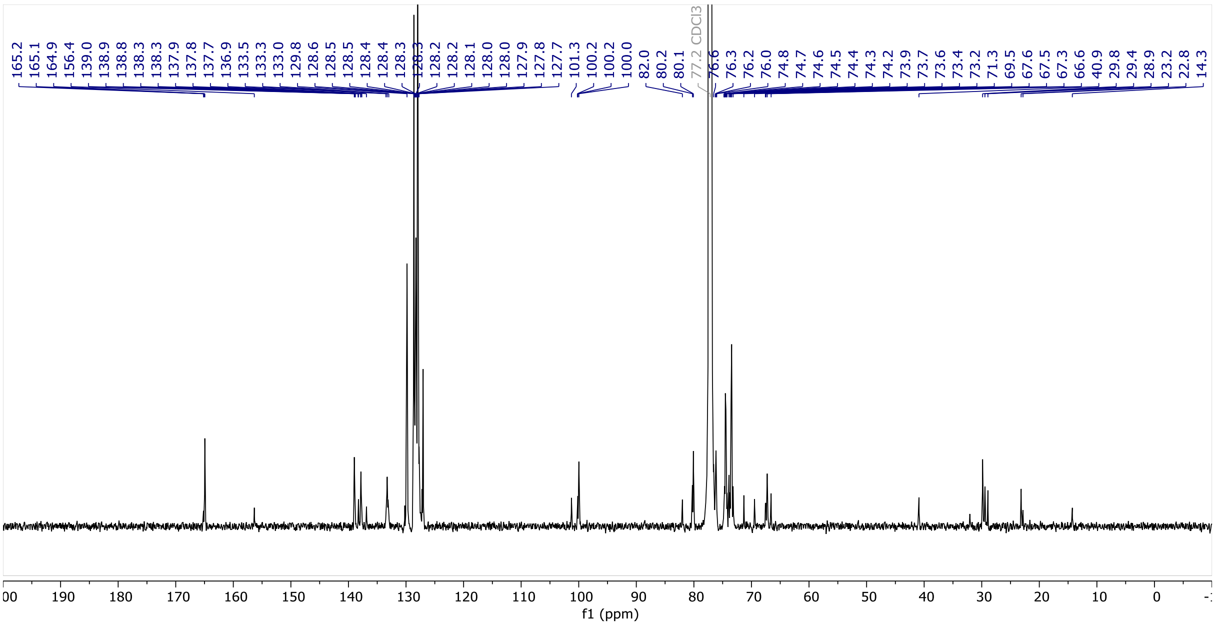

^{13}C,^{1}H HSQC of **10**:

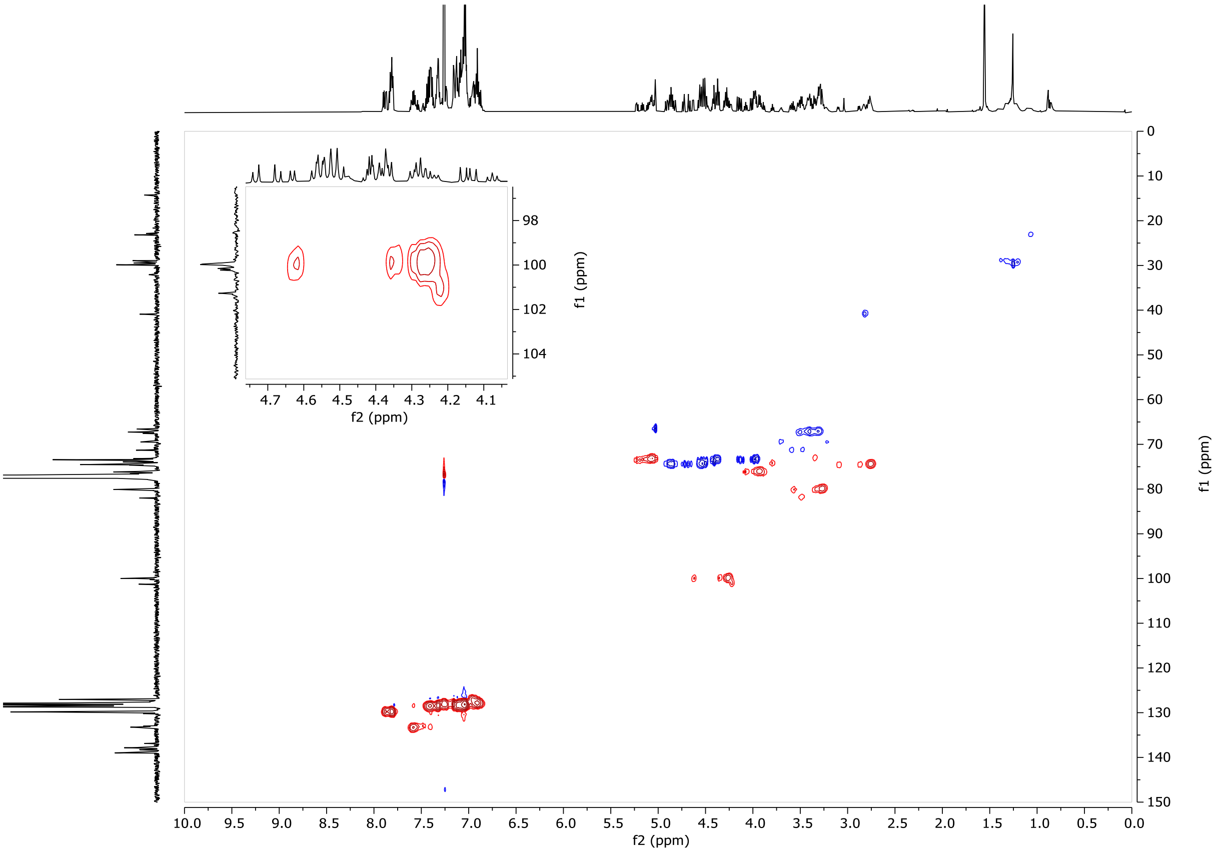

5.2 Lewis Antigen 11

***N*-Benzyloxycarbonyl-5-amino-pentyl 4,6-di-*O*-benzyl-β-D-galactopyranosyl-(1→4)-6-*O*-benzyl-3-*O*-[2,3,4-tri-*O*-benzyl-α-L-fucopyranosyl]-2-deoxy-2-*N*-trichloroacetyl-β-D-glucopyranosyl-(1→3)-2-*O*-benzoyl-4,6-di-*O*-benzyl-β-D-galactopyranoside (11)**

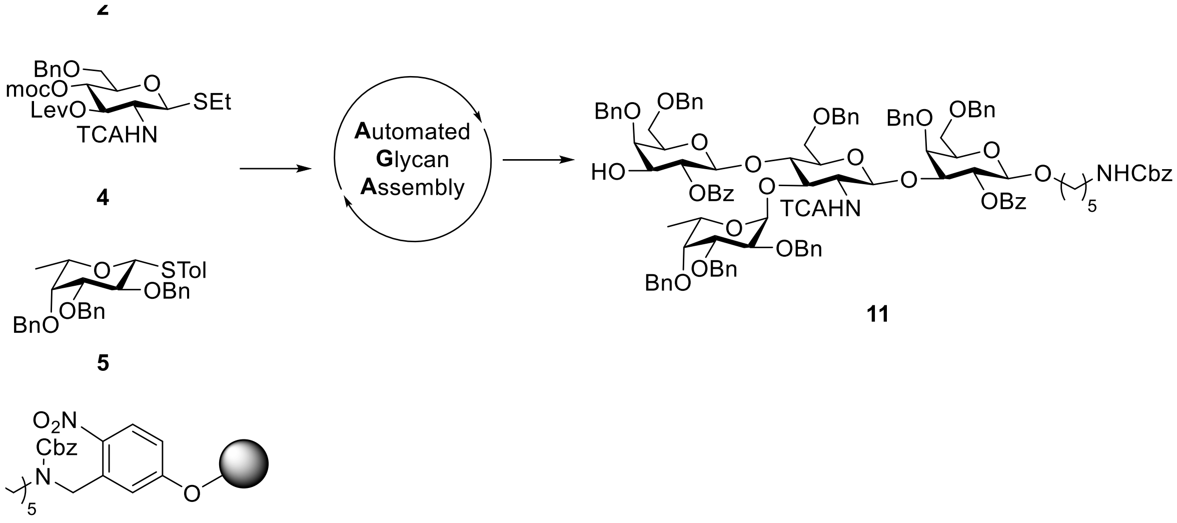

9

Repeat	Building Blocks	Modules	Notes
1x		**I** – Acidic Wash	
	**2** (6.5 equiv.)	**IIb** – Glycosylation with thioglycoside	-30 °C (T_1) 8 min (t_1); -10 °C (T_2) 10 min (t_2)
		**IVa** – Fmoc Deprotection	
1x		**I** – Acidic Wash	
	**4** (6.5 equiv.)	**IIb** – Glycosylation with thioglycoside (2 cycles)	-30 °C (T_1) 10 min (t_1); -5 °C (T_2) 30 min (t_2)
		**IVb** – Lev Deprotection	
1x		**I** – Acidic Wash	
	**5** (6.5 equiv.)	**IIb** – Glycosylation with thioglycoside	-30 °C (T_1) 8 min (t_1); -5 °C (T_2) 10 min (t_2)
		**IVa** – Fmoc Deprotection	
1x		**I** – Acidic Wash	
	**2** (6.5 equiv.)	**IIb** – Glycosylation with thioglycoside	-30 °C (T_1) 8 min (t_1); -5 °C (T_2) 10 min (t_2)
		**IVa** – Fmoc Deprotection	

Protected **11** was obtained after photocleavage from solid support following **Method A-1**. The crude residue was then purified by normal phase flash chromatography (SiO_2, Hex/EtOAc) or

preparative HPLC (**Method B-4**) to afford compound **11** (14 mg, 0.0072 mmol, 53%) as a colorless oil.

^{1}H NMR (700 MHz, $CDCl_3$): δ 7.96 (d, *J* = 6.9 Hz, 2H), 7.93 (d, *J* = 7.0 Hz, 2H), 7.62 (t, *J* = 7.4 Hz, 1H), 7.49 – 7.43 (m, 3H), 7.37 – 7.16 (m, 46H), 6.50 (d, *J* = 8.5 Hz, 1H), 5.46 (dd, *J* = 10.2, 7.8 Hz, 1H), 5.14 (d, *J* = 3.8 Hz, 1H), 5.10 (dd, *J* = 10.1, 8.0 Hz, 1H), 5.05 (s, 2H), 4.89 (d, *J* = 11.8 Hz, 1H), 4.76 – 4.61 (m, 8H), 4.58 – 4.52 (m, 5H), 4.46 (d, *J* = 6.5 Hz, 1H), 4.42 (d, *J* = 12.0 Hz, 1H), 4.39 – 4.30 (m, 6H), 4.25 (d, *J* = 11.5 Hz, 1H), 4.13 (t, *J* = 8.0 Hz, 1H), 4.00 (d, *J* = 2.9 Hz, 1H), 3.96 – 3.92 (m, 3H), 3.90 (dd, *J* = 10.3, 3.0 Hz, 2H), 3.83 – 3.78 (m, 3H), 3.69 – 3.59 (m, 4H), 3.55 – 3.52 (m, 2H), 3.48 (dd, *J* = 9.2, 5.1 Hz, 1H), 3.45 – 3.41 (m, 1H), 3.39 (s, 1H), 3.30 – 3.24 (m, 2H), 2.83 (q, *J* = 6.7 Hz, 2H), 2.20 (d, *J* = 9.5 Hz, 1H), 1.36 – 1.17 (m, 6H), 1.13 (d, *J* = 6.5 Hz, 3H) ppm.

^{13}C NMR (176 MHz, $CDCl_3$): δ 166.3, 165.2, 161.4, 156.4, 139.2, 139.1, 138.9, 138.6, 138.1, 138.1, 137.9, 137.7, 136.9, 133.5, 133.3, 130.2, 129.9, 129.8, 129.1, 128.9, 128.8, 128.7, 128.7, 128.6, 128.6, 128.5, 128.5, 128.4, 128.3, 128.2, 128.2, 128.2, 128.1, 128.0, 127.9, 127.7, 127.6, 127.5, 127.5, 127.1, 101.9, 100.5, 99.5, 97.3, 92.1, 79.7, 78.9, 78.6, 76.4, 76.3, 76.1, 75.8, 75.4, 75.1, 74.7, 74.2, 74.1, 74.0, 73.7, 73.7, 73.5, 73.5, 73.3, 73.2, 73.1, 73.0, 72.6, 69.4, 69.2, 68.2, 67.6, 66.7, 66.6, 40.9, 29.9, 29.5, 29.0, 23.2, 16.6 ppm.

HRMS (QToF): Calcd for $C_{109}H_{115}Cl_3N_2O_{24}Na$ $[M + Na]^+$ 1963.6797; found 1963.6837.

NP-HPLC of crude **11** (ELSD trace, **Method B-3**, t_R = 26.5 min):

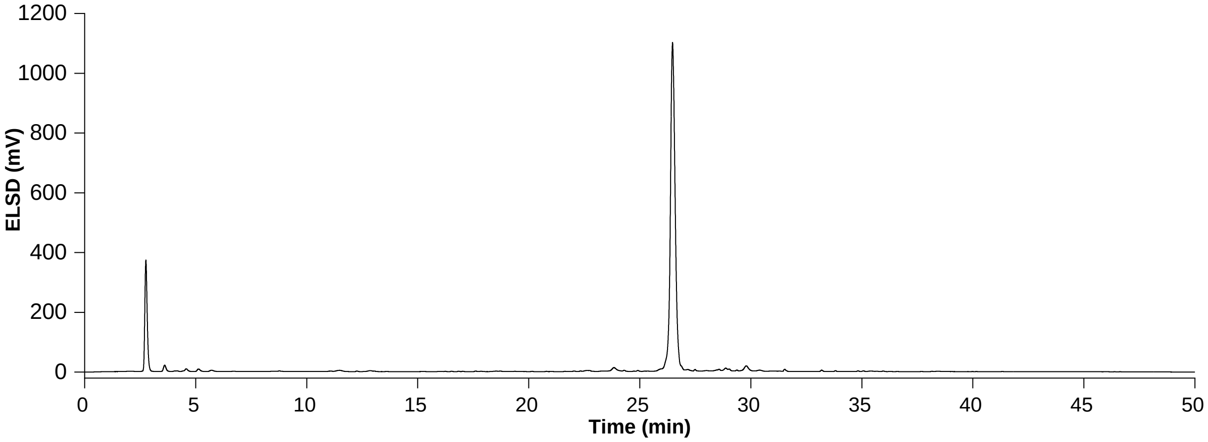

NP-HPLC of purified **11** (ELSD trace, **Method B-3**, t_R = 26.5 min):

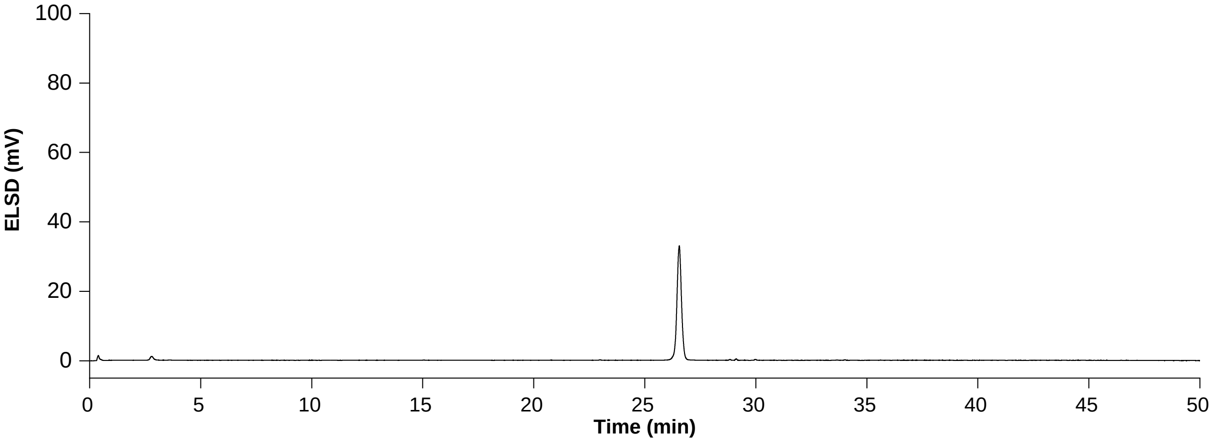

Temperature profile inside the reaction vessel during the synthesis of **11**:

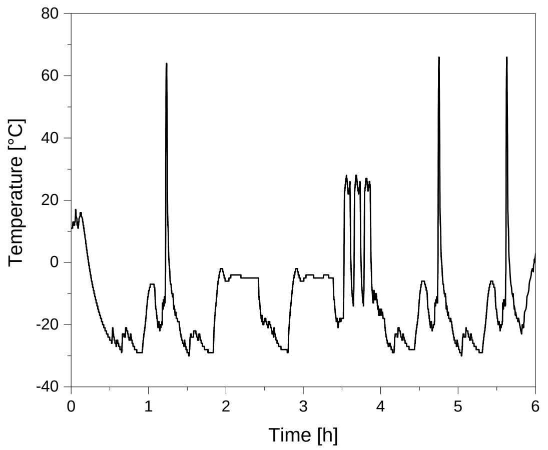

^{1}H NMR (700 MHz, $CDCl_3$) of **11**:

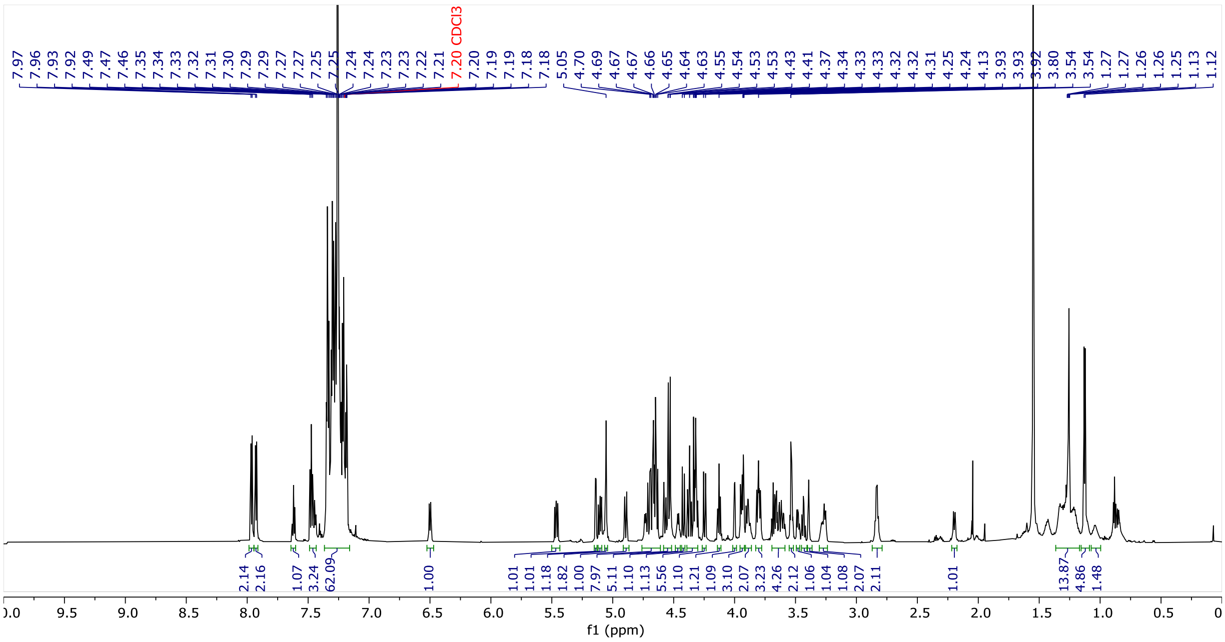

^{13}C NMR (176 MHz, $CDCl_3$) of **11**:

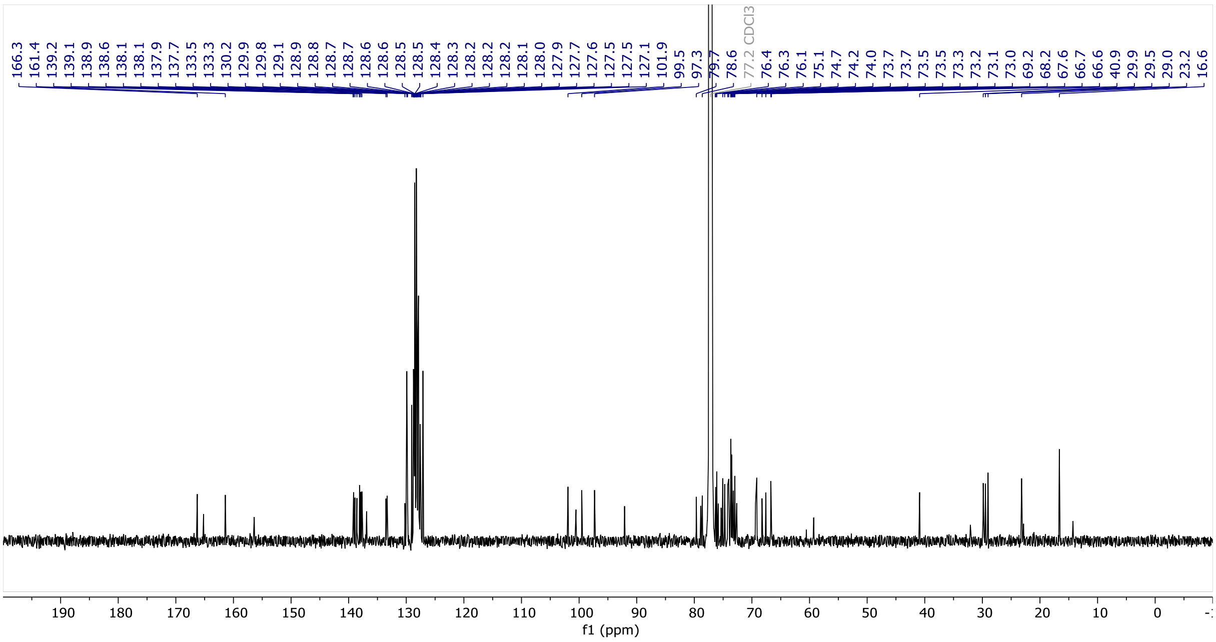

^{13}C,^{1}H HSQC of **11**:

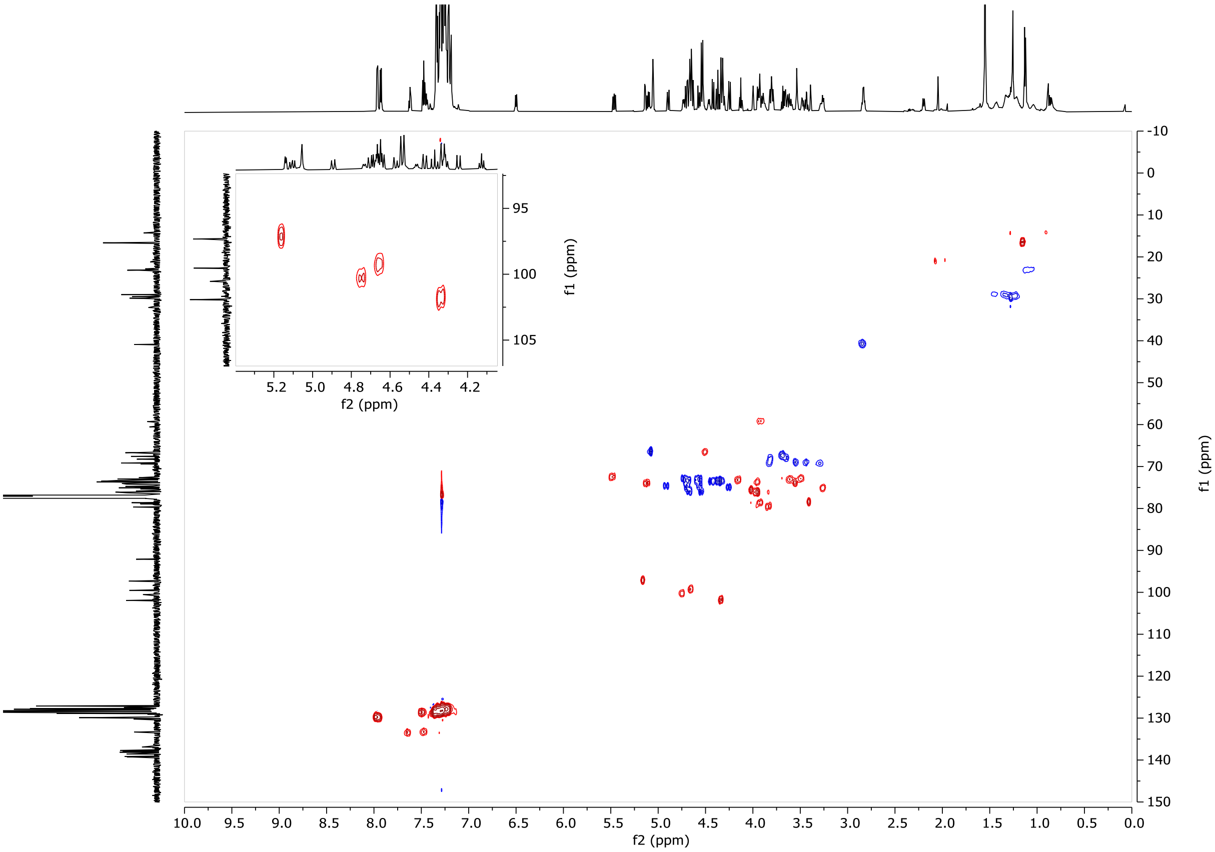

5.3 Mannose Octamer 12

***N*-Benzyloxycarbonyl-5-amino-pentyl 2-*O*-benzoyl-3,4-di-*O*-benzyl-α-D-mannopyranosyl-(1→6)-2-*O*-benzoyl-3,4-di-*O*-benzyl-α-D-mannopyranosyl-(1→6)-2-*O*-benzoyl-3,4-di-*O*-benzyl-α-D-mannopyranosyl-(1→6)-2-*O*-benzoyl-3,4-di-*O*-benzyl-α-D-mannopyranosyl-(1→6)-2-*O*-benzoyl-3,4-di-*O*-benzyl-α-D-mannopyranosyl-(1→6)-2-*O*-benzoyl-3,4-di-*O*-benzyl-α-D-mannopyranosyl-(1→6)-2-*O*-benzoyl-3,4-di-*O*-benzyl-α-D-mannopyranosyl-(1→6)-2-*O*-benzoyl-3,4-di-*O*-benzyl-α-D-mannopyranoside (12)**

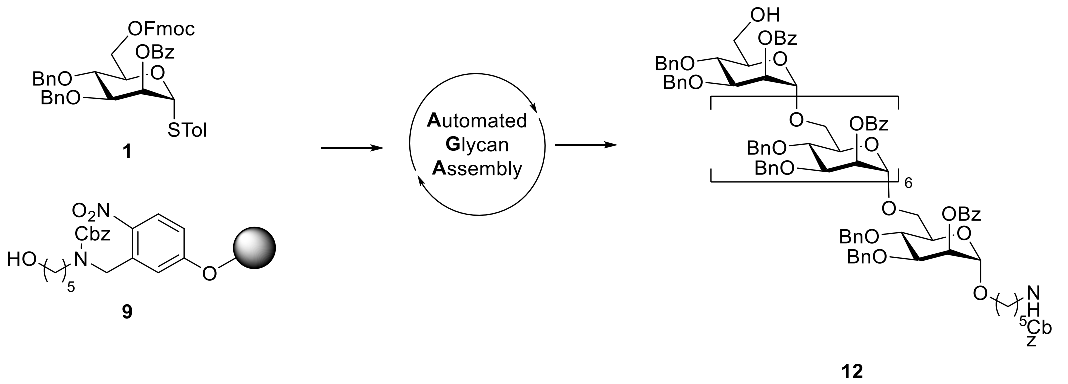

Repeat	Building Blocks	Modules	Notes
**4x**		**I** – Acidic Wash	
	**1** (6.5 equiv.)	**IIb** – Glycosylation with thioglycoside	-20 °C (T_1) 8 min (t_1)<br>0 °C (T_2) 5 min (t_2)
		**III** – Capping<br>**IVa** – Fmoc Deprotection	
**4x**		**I** – Acidic Wash	
	**1** (6.5 equiv.)	**IIb** – Glycosylation with thioglycoside	-20 °C (T_1) 8 min (t_1)<br>0 °C (T_2) 10 min (t_2)
		**III** – Capping<br>**IVa** – Fmoc Deprotection	

Protected **12** was obtained after photocleavage from solid support following **Method A-1**. The crude residue was then purified by normal phase flash chromatography (SiO_2, Hex/EtOAc) or preparative HPLC (**Method B-4**) to afford compound **12** (23 mg, 0.006 mmol, 45%) as a colorless oil.

^{1}H NMR (700 MHz, $CDCl_3$): δ 8.18 (t, *J* = 8.4 Hz, 12H), 8.13 (d, *J* = 8.0 Hz, 2H), 8.10 (d, *J* = 8.2 Hz, 2H), 7.60 (t, *J* = 7.5 Hz, 1H), 7.55 – 7.46 (m, 23H), 7.37 – 7.29 (m, 12H), 7.27 – 7.05 (m, 72H), 5.87 – 5.80 (m, 6H), 5.79 (s, 1H), 5.64 (s, 1H), 5.13 – 5.03 (m, 9H), 4.94 – 4.77 (m, 18H), 4.73 (d, *J* = 11.3 Hz, 1H), 4.61 (d, *J* = 11.1 Hz, 1H), 4.57 (d, *J* = 11.0 Hz, 1H), 4.49 (dd, *J* = 11.3, 5.6 Hz, 2H), 4.44 (dd, *J* = 15.1, 11.0 Hz, 7H), 4.39 – 4.33 (m, 5H), 4.12 – 4.08 (m,

2H), 4.07 – 4.02 (m, 6H), 4.01 – 3.83 (m, 12H), 3.82 – 3.71 (m, 7H), 3.71 – 3.57 (m, 9H), 3.56 – 3.39 (m, 7H), 3.19 (d, *J* = 8.0 Hz, 2H), 1.52 (m, 4H), 1.37 (m, 2H) ppm.

^{13}C NMR (101 MHz, $CDCl_3$): δ 166.0, 165.7, 165.6, 165.5, 156.5, 138.6, 138.6, 138.6, 138.5, 138.4, 138.4, 138.0, 137.7, 137.6, 137.6, 136.8, 133.5, 130.0, 128.8, 128.7, 128.6, 128.5, 128.5, 128.4, 128.3, 128.3, 128.2, 128.2, 128.1, 127.8, 127.8, 127.8, 127.7, 127.5, 127.3, 127.2, 98.6, 98.5, 98.2, 97.6, 78.7, 78.3, 77.8, 77.4, 75.3, 75.1, 74.3, 74.0, 73.8, 72.2, 71.7, 71.5, 71.4, 71.3, 71.1, 71.0, 70.8, 69.1, 68.6, 68.4, 67.9, 66.7, 66.2, 65.8, 61.9, 41.1, 29.9, 29.2, 23.5 ppm.

HRMS (QToF): Calcd for $C_{229}H_{227}NO_{51}Na_2$ [M + 2Na]$^{2+}$ 1926.2492; found 1926.2581.

NP-HPLC of crude **12** (ELSD trace, **Method B-3**, t_R = 27.0 min):

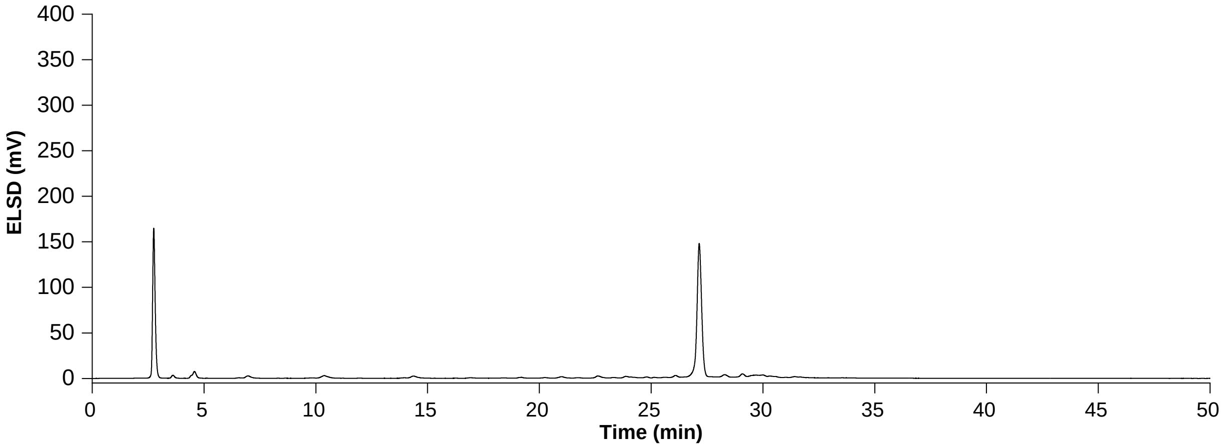

NP-HPLC of purified **12** (ELSD trace, **Method B-3**, t_R = 27.1 min):

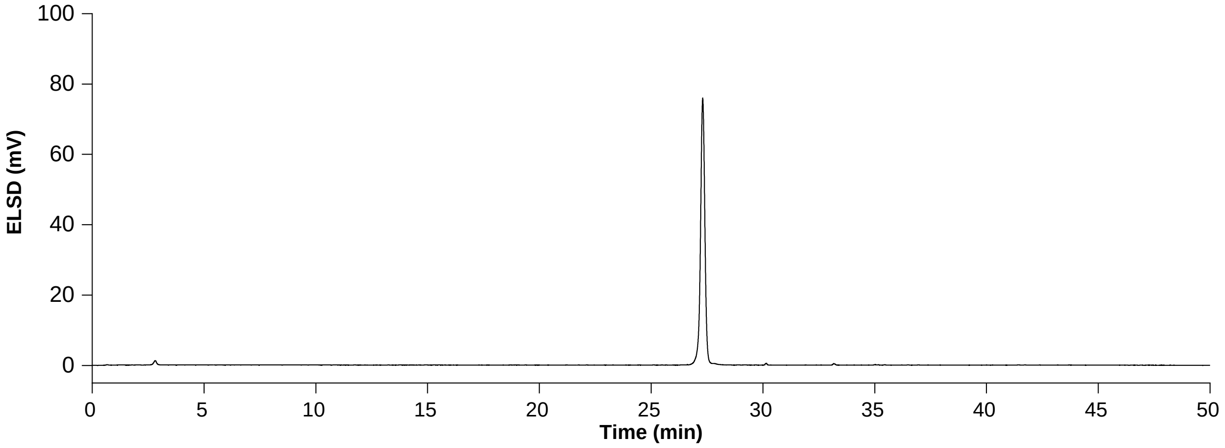

Temperature profile inside the reaction vessel during the synthesis of **12**:

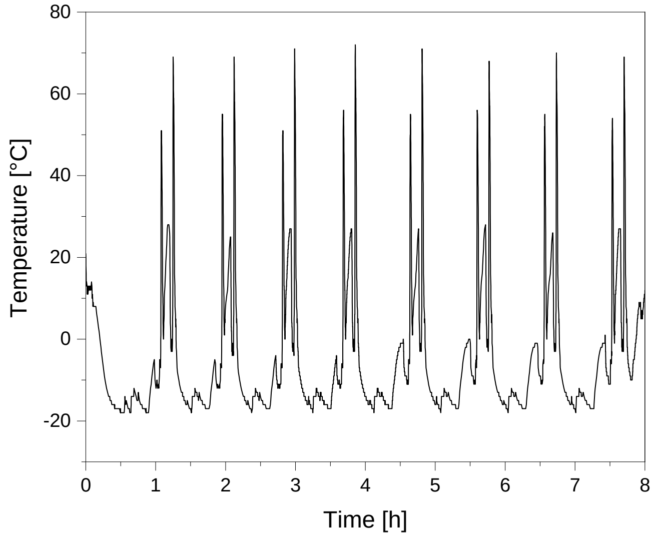

^{1}H NMR (700 MHz, $CDCl_3$) of **12**:

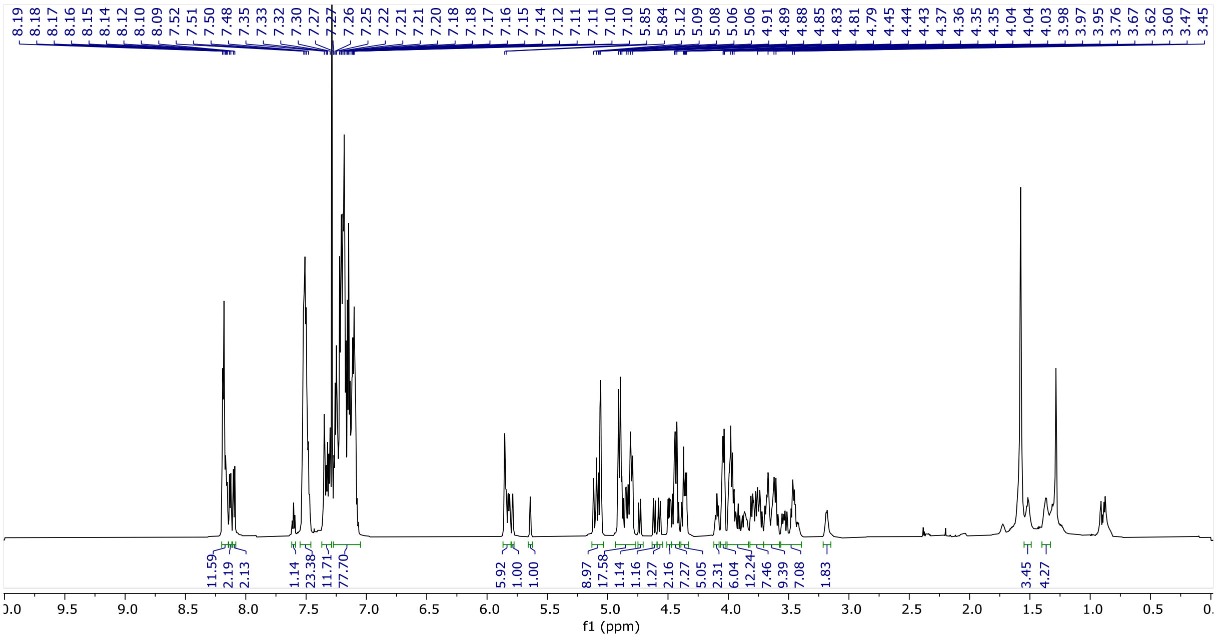

^{13}C NMR (101 MHz, $CDCl_3$) of **12**:

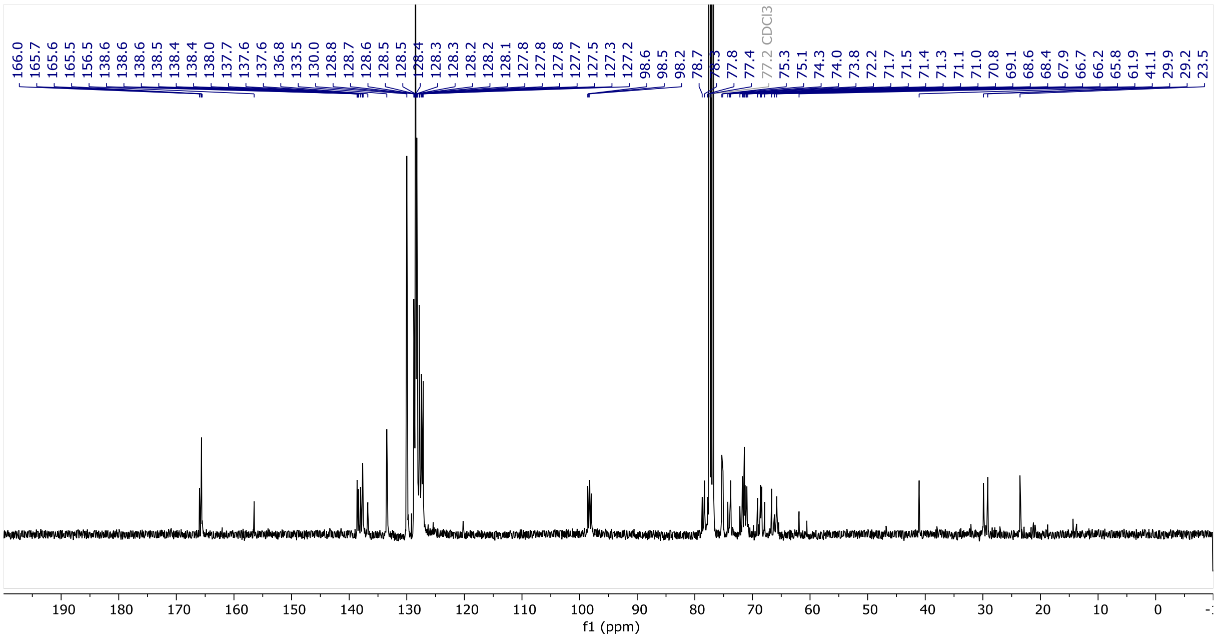

^{13}C,^{1}H HSQC of **12**:

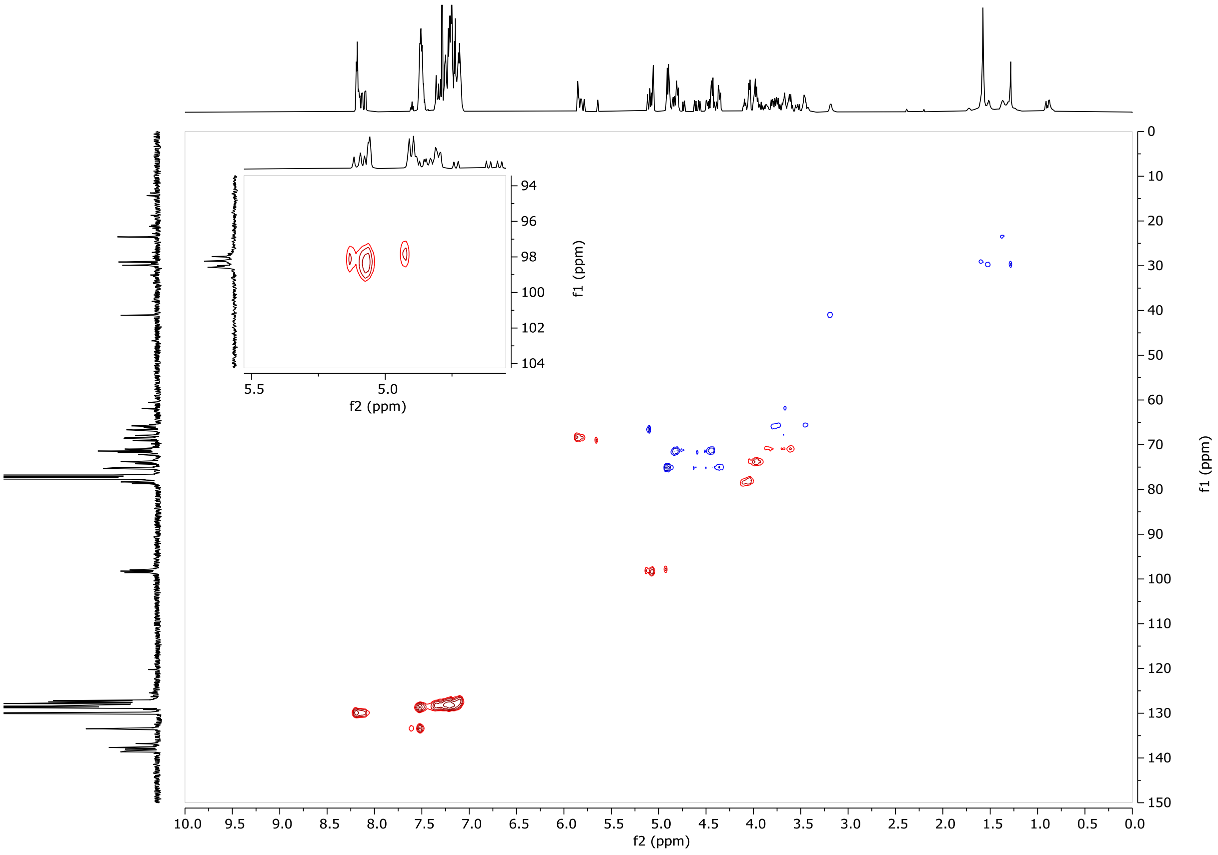

5.4 Keratan Sulfate 13

***N*-Benzyloxycarbonyl-5-amino-pentyl 4,6-di-*O*-benzyl-3-*O*-sulfo-β-D-galactopyranosyl-(1→4)-6-*O*-sulfo-3-*O*-benzyl-2-deoxy-2-*N*-trichloroacetyl-β-D-glucopyranosyl-(1→3)-4,6-di-*O*-benzyl-3-β-D-galactopyranosyl-(1→4)-6-*O*-sulfo-3-*O*-benzyl-2-deoxy-2-*N*-trichloroacetyl-β-D-glucopyranoside Tritrimethylammonium Salt (13)**

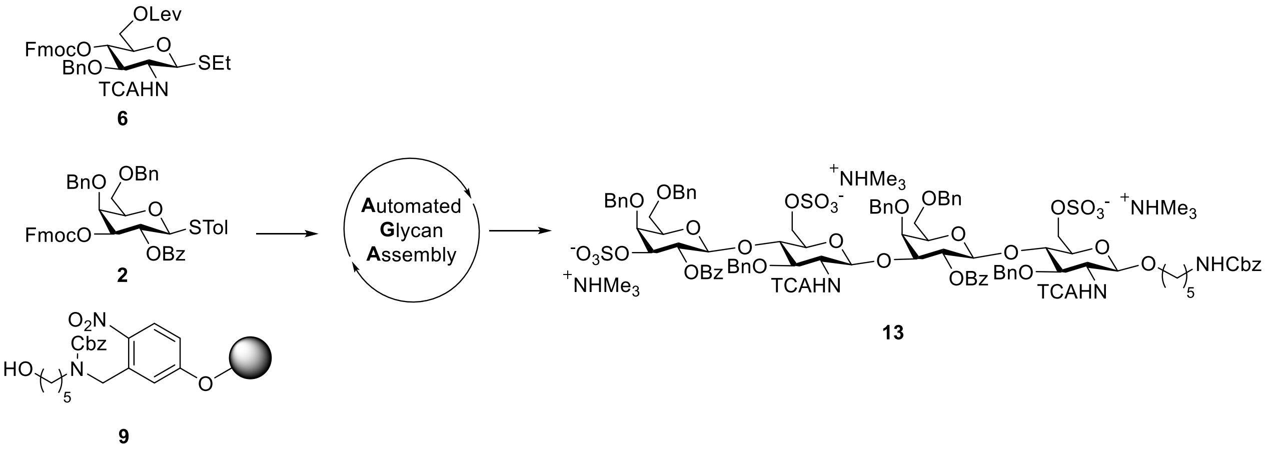

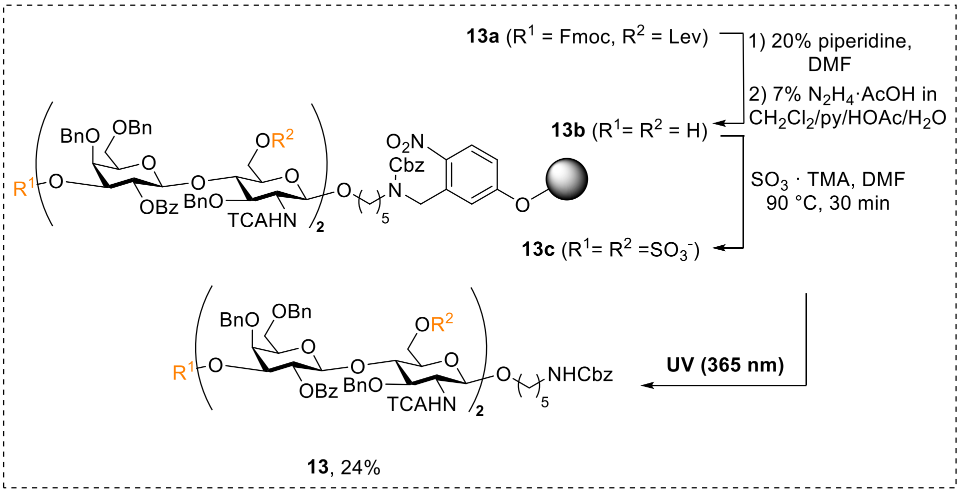

Repeat	Building Blocks	Modules	Notes
2x		**I** – Acidic Wash	
	**6** (6.5 equiv.)	**IIb** – Glycosylation with thioglycoside (2 cycles)	-30 °C (T_1) 10 min (t_1)<br>-5 °C (T_2) 30 min (t_2)
		**III** – Capping<br>**IVa** – Fmoc Deprotection	
		**I** – Acidic Wash	
	**2** (6.5 equiv.)	**IIb** – Glycosylation with thioglycoside (2 cycles)	-30 °C (T_1) 8 min (t_1)<br>-5 °C (T_2) 10 min (t_2)
		**IVa** – Fmoc Deprotection	
1x		**IVb** – Lev Deprotection	
		**V** – Sulfation	

Protected **13** was obtained after photocleavage from solid support following **Method A-3**. The crude residue was then purified by reverse phase flash chromatography (C-18 silica gel, water/acetonitrile) to afford compound **13** (7 mg, 0.0072 mmol, 24%) as a tan oil upon concentration. Note: Fractions were analyzed by mass spectrometry (negative mode) for purity before combination.

^{1}H NMR (700 MHz, MeOD): δ 8.23 (d, *J* = 6.9 Hz, 2H), 8.13 (d, *J* = 6.8 Hz, 2H), 7.62 (t, *J* = 7.4 Hz, 1H), 7.58 (t, *J* = 7.3 Hz, 1H), 7.54 (t, *J* = 7.8 Hz, 2H), 7.50 – 7.44 (m, 4H), 7.42 – 7.37 (m, 2H), 7.34 – 7.16 (m, 35H), 7.14 (t, *J* = 7.1 Hz, 2H), 7.10 (dt, *J* = 9.0, 7.3 Hz, 3H), 7.02 (dt, *J* = 14.9, 7.6 Hz, 4H), 5.56 (dd, *J* = 10.2, 7.9 Hz, 1H), 5.49 (dd, *J* = 10.2, 7.9 Hz, 1H), 5.14 (d, *J* = 9.8 Hz, 2H), 5.09 (d, *J* = 11.4 Hz, 1H), 5.05 – 5.01 (m, 4H), 4.90 (d, *J* = 8.0 Hz, 1H), 4.80 (dd, *J* = 10.2, 3.1 Hz, 1H), 4.68 (d, *J* = 8.3 Hz, 1H), 4.59 (dd, *J* = 21.4, 11.4 Hz, 2H), 4.48 (dd, *J* = 16.7, 10.0 Hz, 2H), 4.42 – 4.35 (m, 3H), 4.31 – 4.18 (m, 4H), 4.13 – 4.01 (m, 3H), 3.92 – 3.84 (m, 3H), 3.78 (dd, *J* = 7.1, 4.9 Hz, 1H), 3.73 – 3.66 (m, 3H), 3.61 (dd, *J* = 10.4, 8.6 Hz, 1H), 3.54 (dd, *J* = 10.6, 4.5 Hz, 1H), 3.47 – 3.38 (m, 3H), 3.35 (dd, *J* = 6.2, 3.6 Hz, 1H), 3.06 – 3.02 (m, 2H), 2.82 (s, 27H), 1.45 (d, *J* = 16.9 Hz, 4H), 1.35 – 1.25 (m, 2H) ppm.

^{13}C NMR (176 MHz, MeOD): δ 167.4, 167.1, 164.2, 158.9, 140.8, 140.6, 140.1, 140.0, 139.7, 138.5, 134.4, 131.6, 131.3, 131.1, 129.3, 128.4, 128.4, 128.3, 128.2, 128.1, 128.0, 103.2, 102.0, 101.8, 101.4, 94.2, 93.7, 81.2, 80.8, 79.7, 79.2, 76.9, 76.7, 76.4, 76.3, 76.0, 75.6, 74.8, 74.7, 74.5, 74.3, 73.7, 72.9, 70.8, 70.4, 69.6, 67.3, 66.4, 66.1, 58.3, 58.0, 57.6, 57.5, 57.4, 45.6, 41.7, 30.5, 30.2, 24.3, 17.4, 17.3, 17.2 ppm.

HRMS (QToF): Calcd for $C_{97}H_{100}Cl_6N_3O_{34}S_3$ $[M]^{3-}$ 718.7833; found 718.7853.

RP-HPLC of crude **13** (UV-Vis trace (230 nm), **Method E-3**, t_R = 22.5 min):

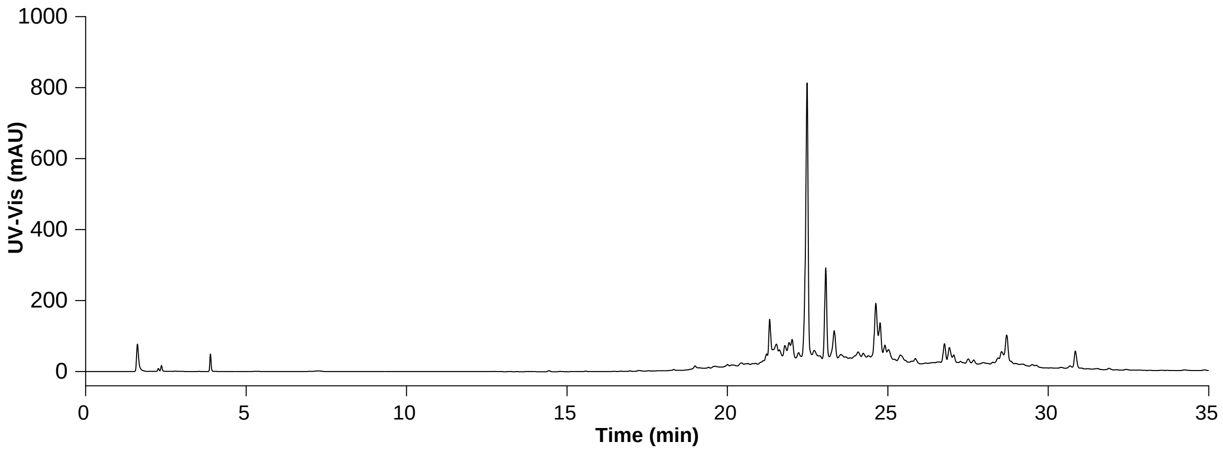

Mass spectrum at 22.5 min:

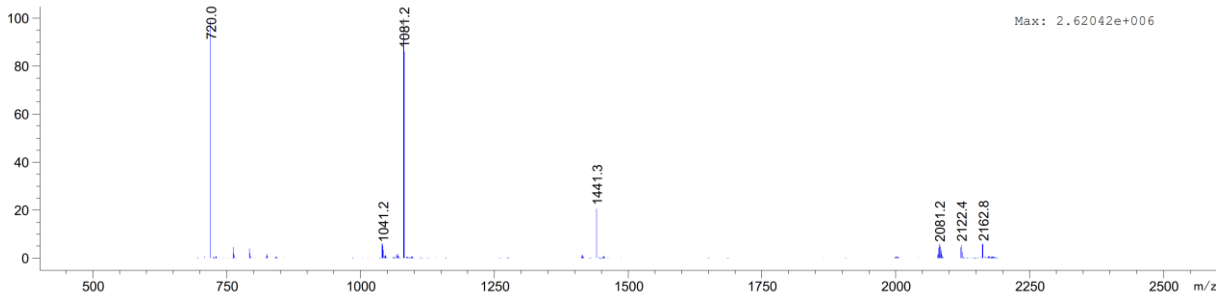

RP-HPLC of purified **13** (UV-Vis trace (230 nm), **Method E-3**, t_R = 21.6 min):

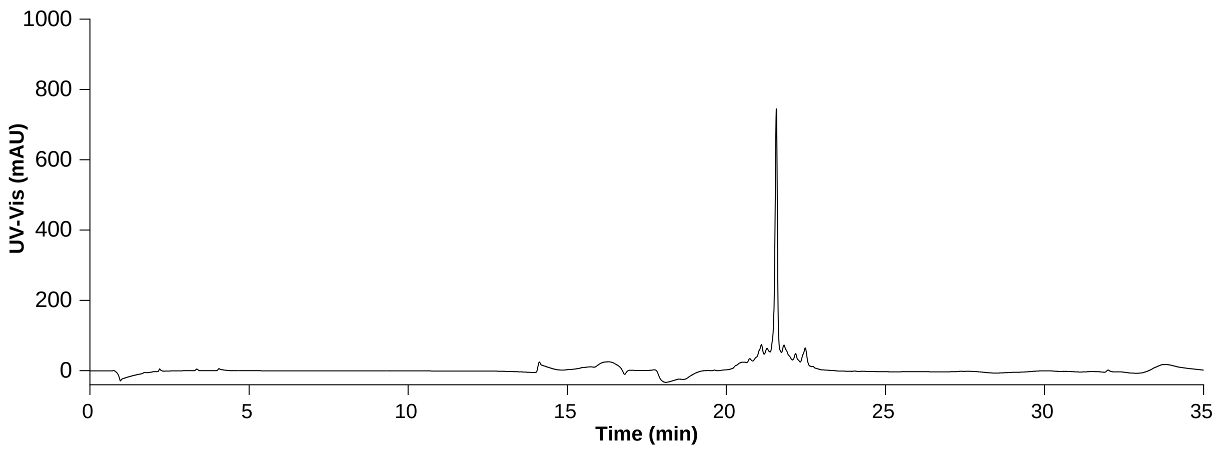

Mass spectrum at 21.6 min

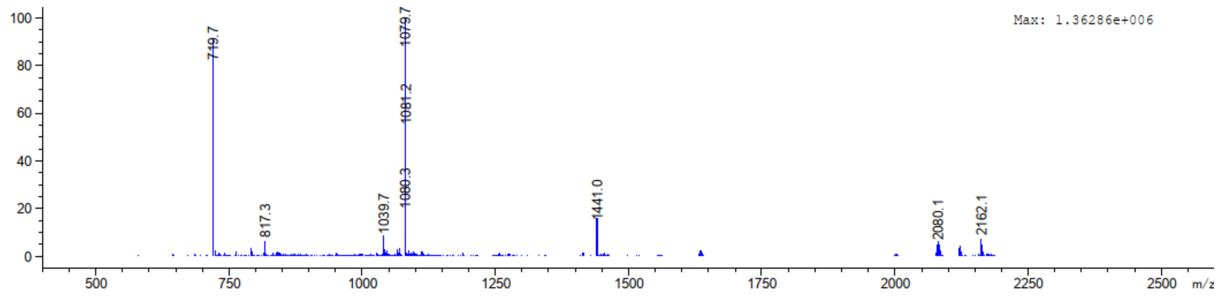

Temperature profile inside the reaction vessel during the synthesis of **13**:

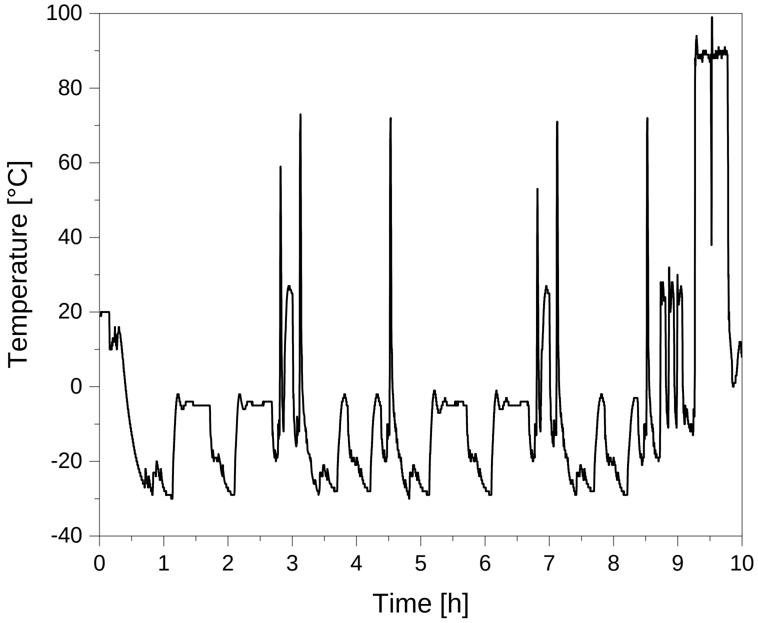

^{1}H NMR (700 MHz, $CDCl_3$) of **13**:

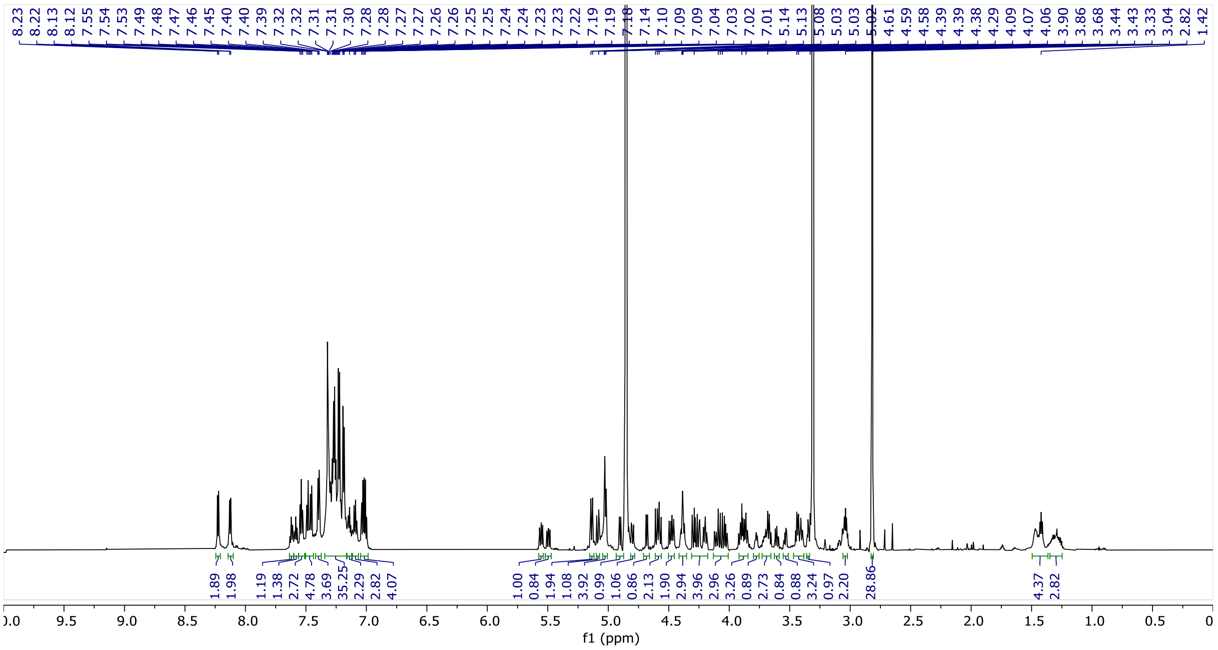

^{13}C NMR (176 MHz, $CDCl_3$) of **13**:

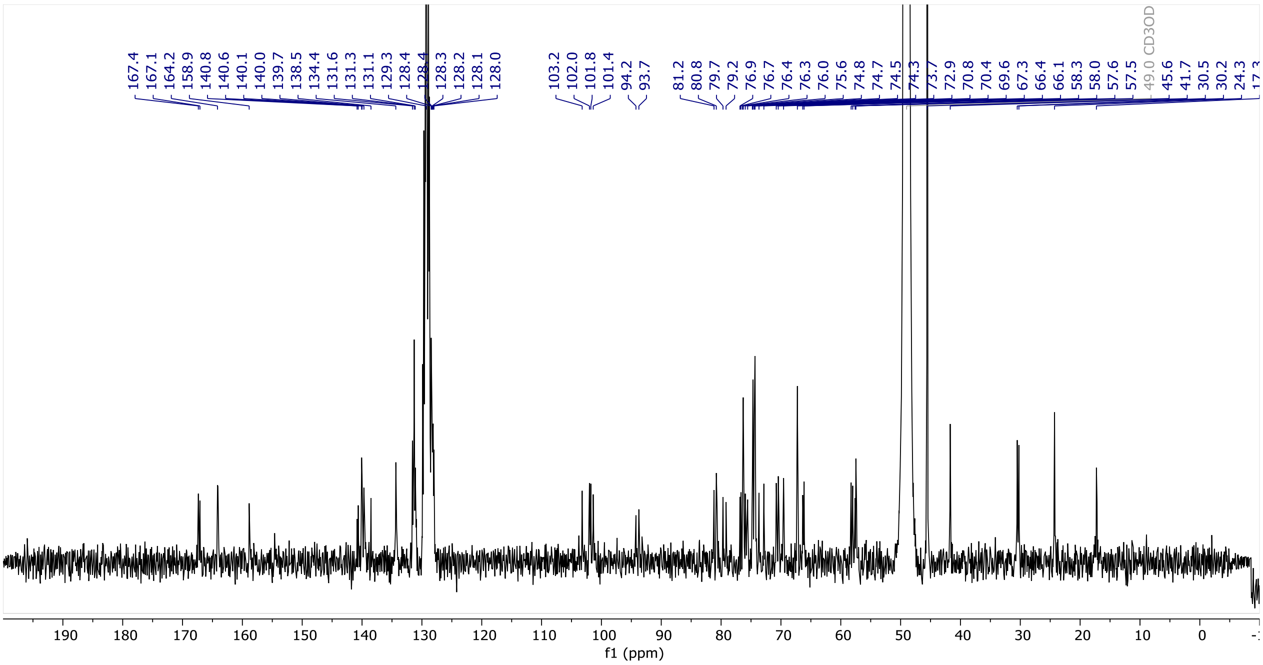

^{13}C,^{1}H HSQC of **13**:

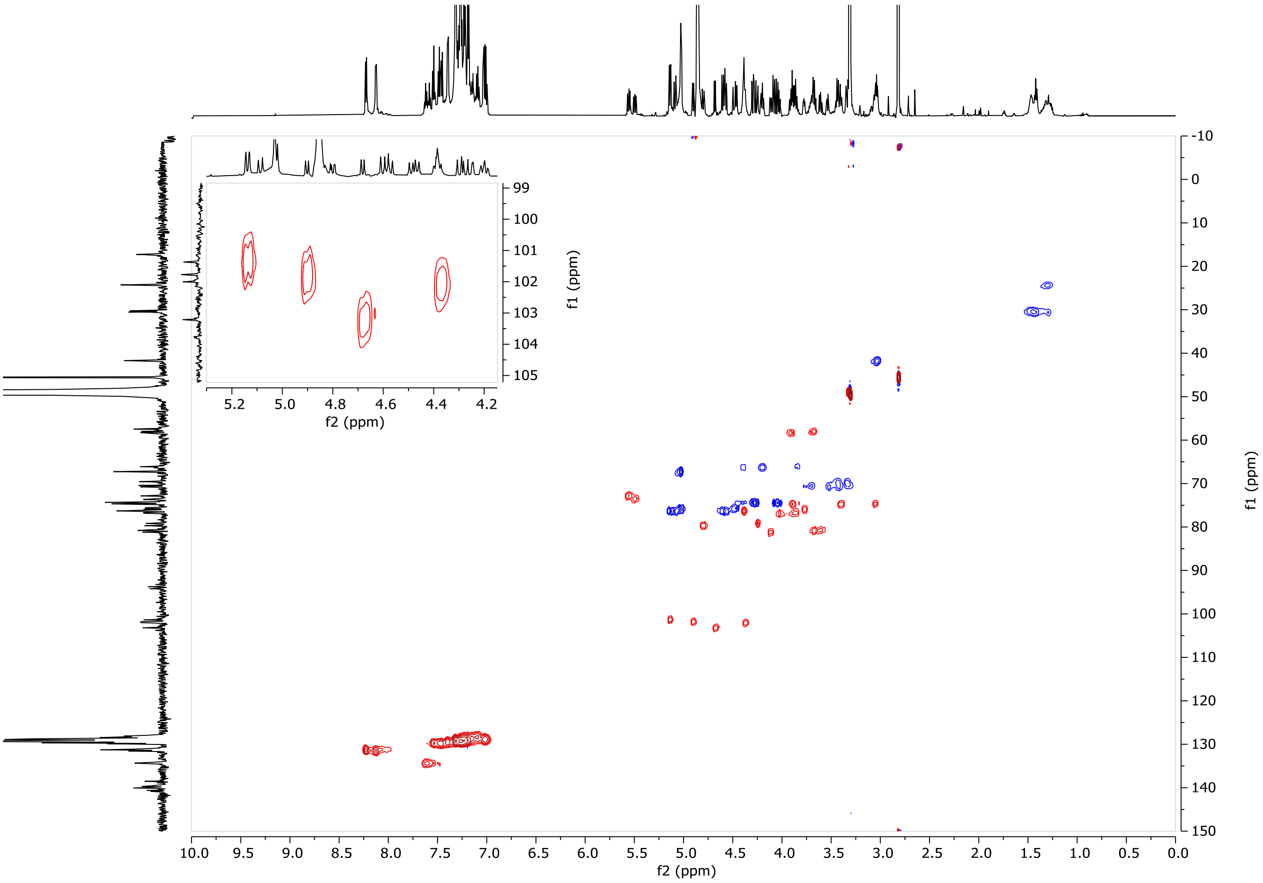

5.5 Automated Glycan Assembly Using an Orthogonal Building Block

5.5.1 Linear Mannose Tetramers (14-17)

5-Amino-pentyl α-(1→4)-D-tetramannopyranoside (16)

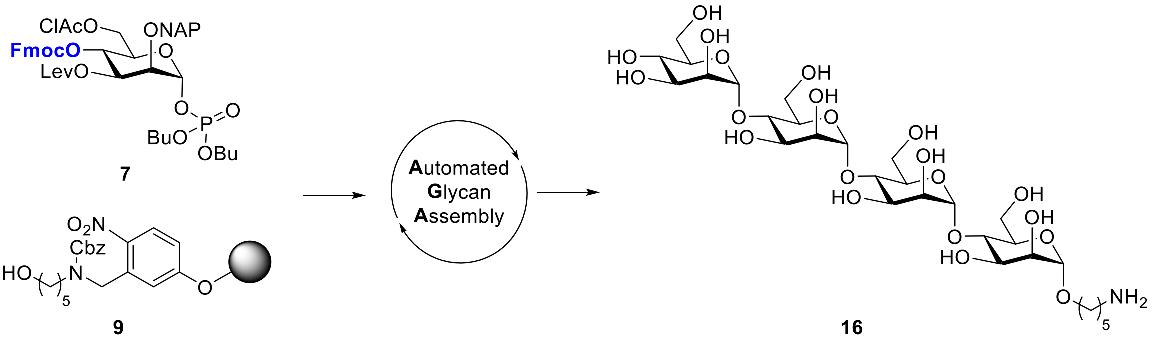

Repeat	Building Blocks	Modules	Notes
1x		**I** – Acidic Wash	
	**7** (2 x 4.7 equiv.)	**IIb** – Glycosylation with glycosyl phosphate – 2 cycles	-20 °C (T_1) 30 min (t_1)<br>0 °C (T_2) 10 min (t_2)
		**III** – Capping<br>**IVc** – Fmoc Deprotection	
3x		**I** – Acidic Wash	
	**7** (4.7 equiv.)	**IIb** – Glycosylation with glycosyl phosphate	-20 °C (T_1) 30 min (t_1)<br>0 °C (T_2) 10 min (t_2)
		**III** – Capping<br>**IVc** – Fmoc Deprotection	

Protected **16** (15 mg, 0.007 mmol, crude yield: 52%) was obtained as a colorless oil after photocleavage from solid support following **Method A-1**. Deprotection of **16** following **Method C** and **D** and purification by reverse-phase HPLC (**Method E-1**, t_R = 17.3 min) afforded deprotected compound **16** (1.8 mg, 0.002 mmol, 18%) as a white solid after lyophylization.

^{1}H NMR (700 MHz, D_2O): δ 5.28 – 5.24 (m, 3H), 4.88 (d, *J* = 1.8 Hz, 1H), 4.07 (dd, *J* = 3.3, 1.9 Hz, 1H), 4.03 (dd, *J* = 3.4, 1.9 Hz, 2H), 3.99 – 3.66 (m, 22H), 3.57 (dt, *J* = 10.0, 6.2 Hz, 1H), 3.04 – 3.00 (m, 2H), 1.75 – 1.62 (m, 4H), 1.52 – 1.41 (m, 2H) ppm.

^{13}C NMR (176 MHz, D_2O): δ 101.4, 101.3, 99.6, 74.3, 74.1, 74.1, 73.7, 72.3, 72.2, 71.2, 71.1, 70.8, 70.8, 70.7, 70.7, 70.6, 70.4, 70.3, 67.6, 66.5, 61.0, 61.0, 60.9, 39.4, 28.0, 26.8, 22.4 ppm.

HRMS (QToF): Calcd for $C_{29}H_{53}NO_{21}$ $[M + H]^+$ 752.3183; found 752.3205.

RP-HPLC of crude deprotected **16** (ELSD trace, **Method E-1**, t_R = 17.3 min):

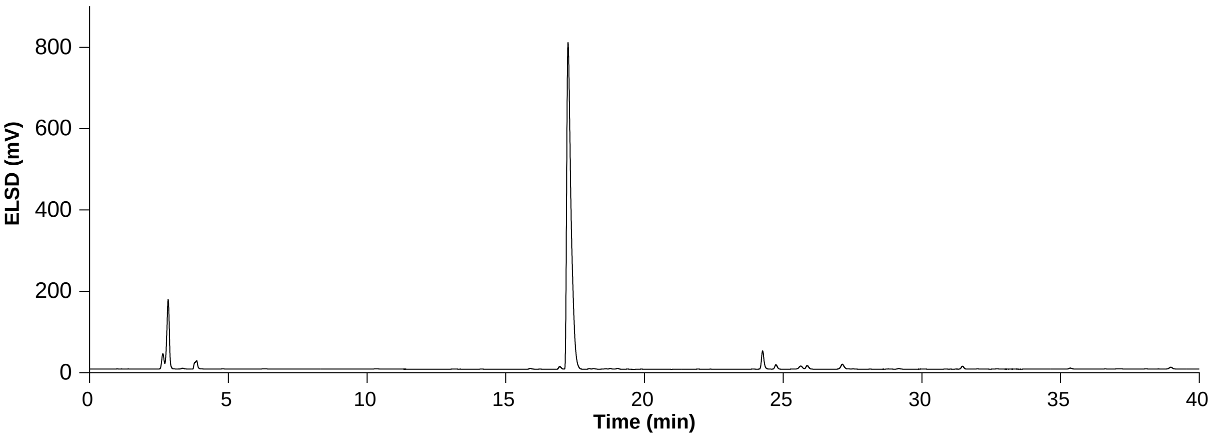

RP-HPLC of purified deprotected **16** (ELSD trace, **Method E-1**, t_R = 17.3 min):

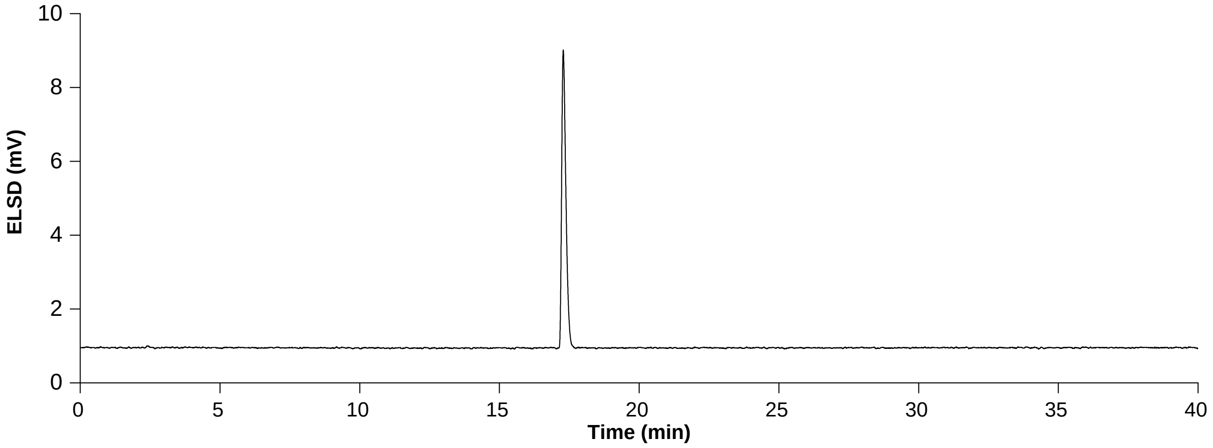

Temperature profile inside the reaction vessel during one synthesis cycle of **16**:

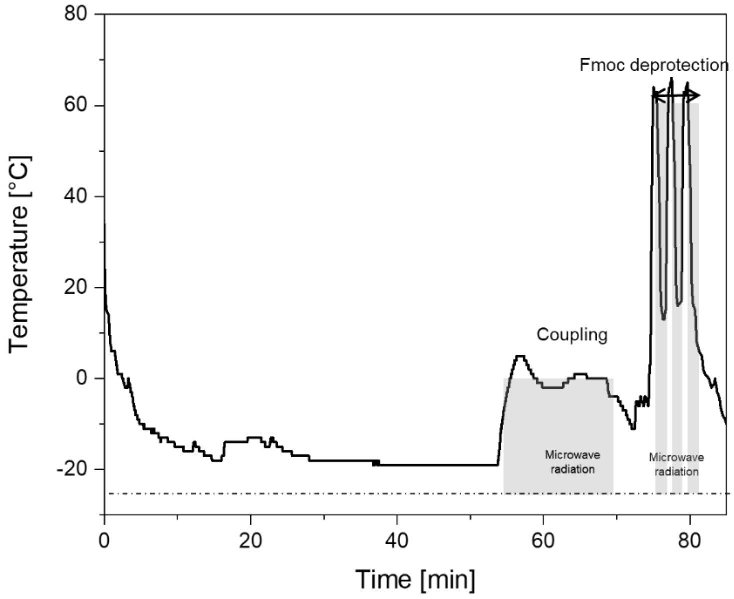

^{1}H NMR (700 MHz, D_2O) of **16**:

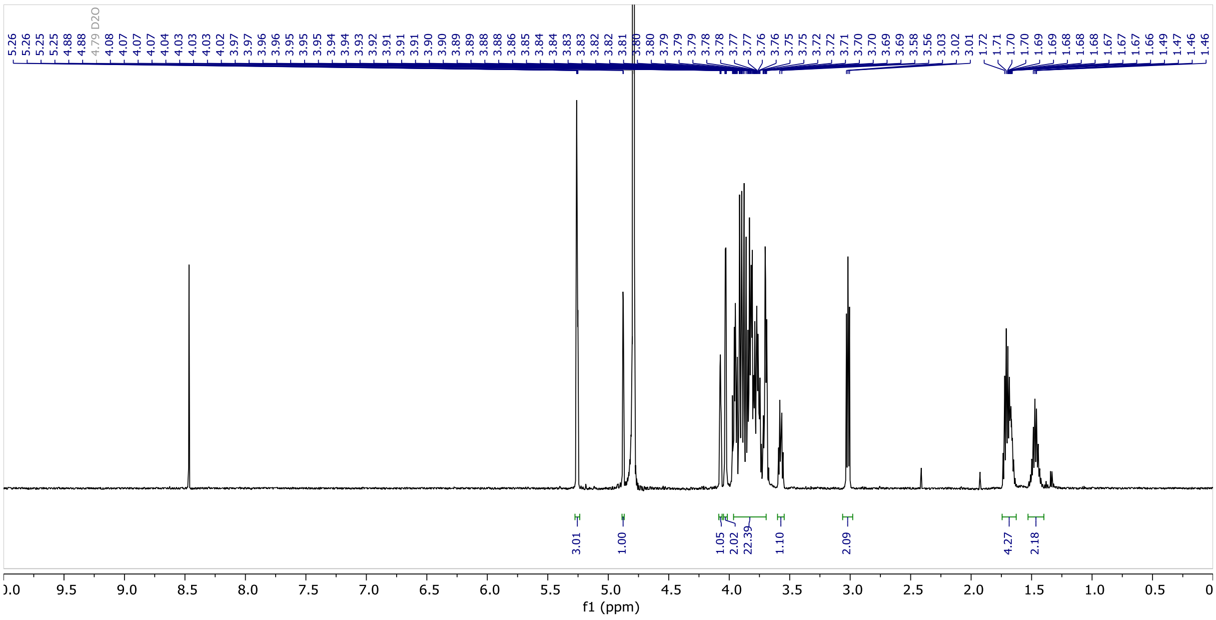

^{13}C NMR (176 MHz, D_2O) of **16**:

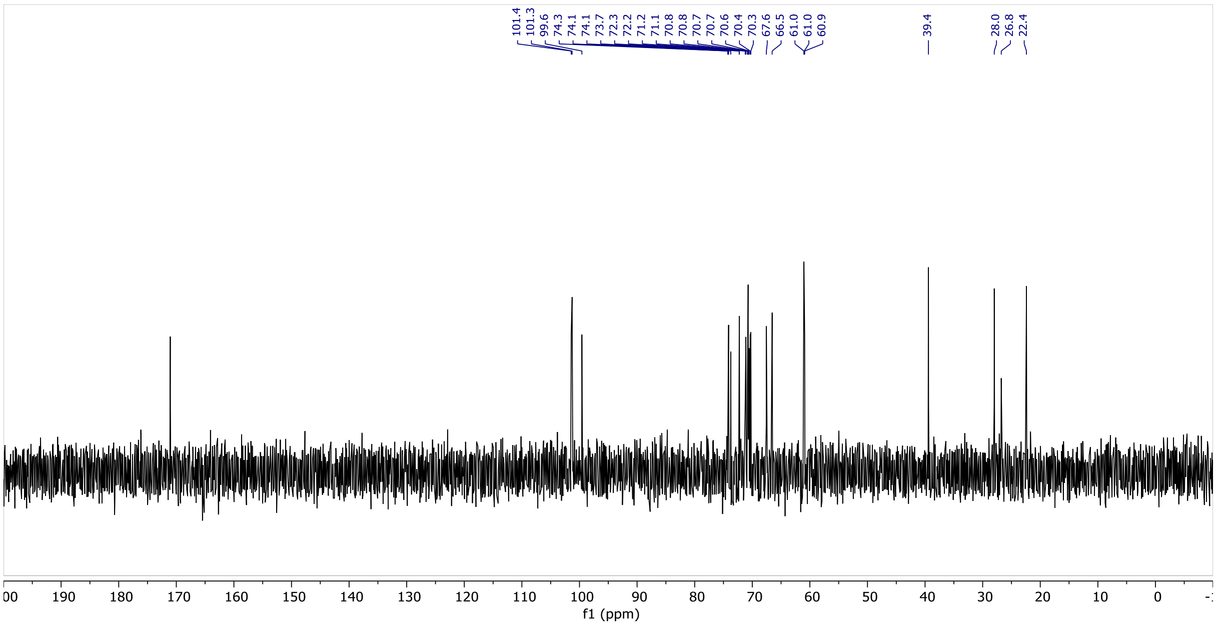

Coupled $^{13}C,^{1}H$ HSQC of **16**:

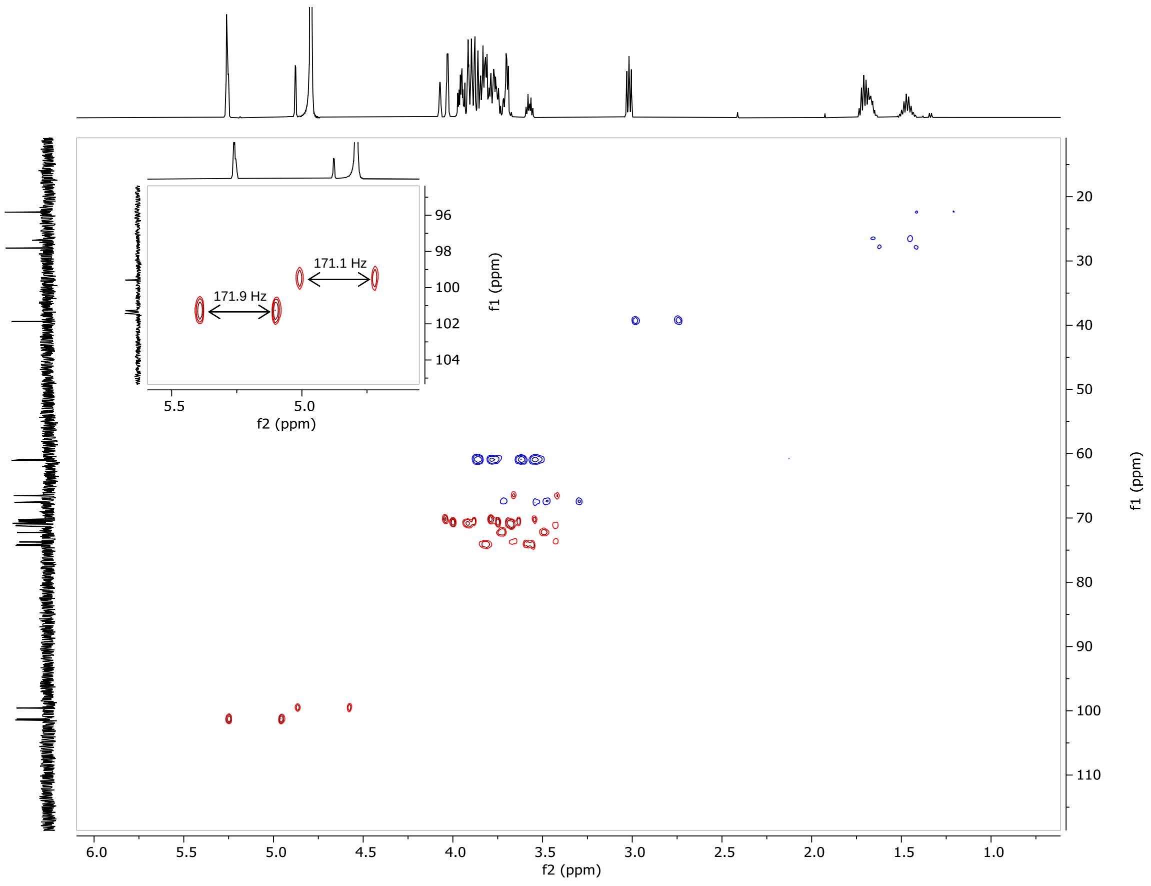

5-Amino-pentyl α-(1→3)-D-tetramannopyranoside (15)

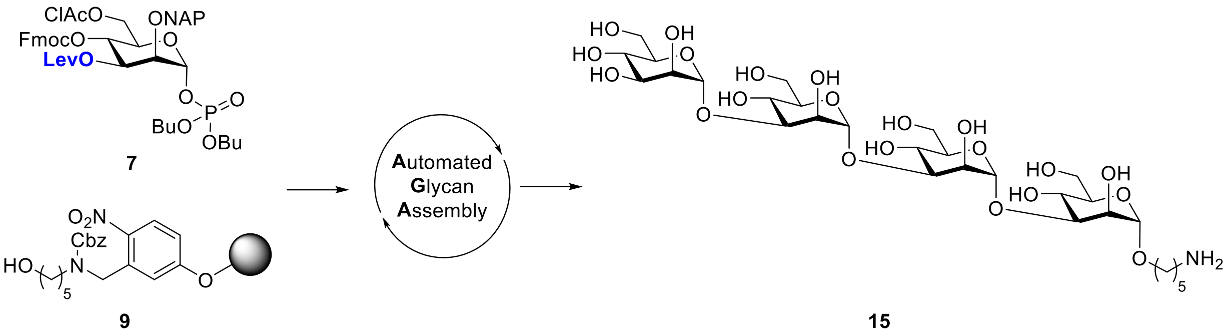

Repeat	Building Blocks	Modules	Notes	
1x		**I** – Acidic Wash		
	**7** (2 x 4.7 equiv.)	**IIb** – Glycosylation with glycosyl phosphate – 2 cycles	-20 °C (T_1)<br>0 °C (T_2)	30 min (t_1)<br>10 min (t_2)
		**III** – Capping<br>**IVb** – Lev Deprotection		
3x		**I** – Acidic Wash		
	**7** (4.7 equiv.)	**IIb** – Glycosylation with glycosyl phosphate	-20 °C (T_1)<br>0 °C (T_2)	30 min (t_1)<br>10 min (t_2)
		**III** – Capping<br>**IVb** – Lev Deprotection		

Protected **15** (26 mg, 0.010 mmol, crude yield: 74%) was obtained as a colorless oil after photocleavage from solid support following **Method A-1**. Deprotection of **15** following **Method C** and **D** and purification by reverse-phase HPLC (**Method E-1**, t_R = 18.0 min) afforded deprotected compound **15** (1.2 mg, 0.002 mmol, 12%) as a white solid after lyophylization.

^{1}H NMR (700 MHz, D_2O): δ 5.15 (d, *J* = 1.8 Hz, 1H), 5.13 (d, *J* = 1.7 Hz, 1H), 5.12 (d, *J* = 1.9 Hz, 1H), 4.86 (d, *J* = 1.4 Hz, 1H), 4.26 – 4.23 (m, 1H), 4.12 – 4.07 (m, 2H), 4.06 – 4.00 (m, 2H), 3.96 – 3.73 (m, 12H), 3.71 – 3.62 (m, 2H), 3.57 (dt, *J* = 9.4, 5.9 Hz, 1H), 3.02 (t, *J* = 7.6 Hz, 2H), 1.75 – 1.62 (m, 4H), 1.54 – 1.40 (m, 2H) ppm.

^{13}C NMR (176 MHz, D_2O): δ 102.3, 102.2, 102.2, 99.6, 78.3, 78.1, 78.1, 73.6, 73.5, 73.4, 72.9, 70.4, 70.0, 69.7, 69.7, 67.5, 66.9, 66.2, 66.1, 66.1, 61.1, 61.0, 61.0, 60.9, 39.4, 28.0, 26.6, 22.5 ppm.

HRMS (QToF): Calcd for $C_{29}H_{53}NO_{21}$ $[M + H]^+$ 752.3183; found 752.3218.

RP-HPLC of crude deprotected **15** (ELSD trace, **Method E-1**, t_R = 18.0 min):

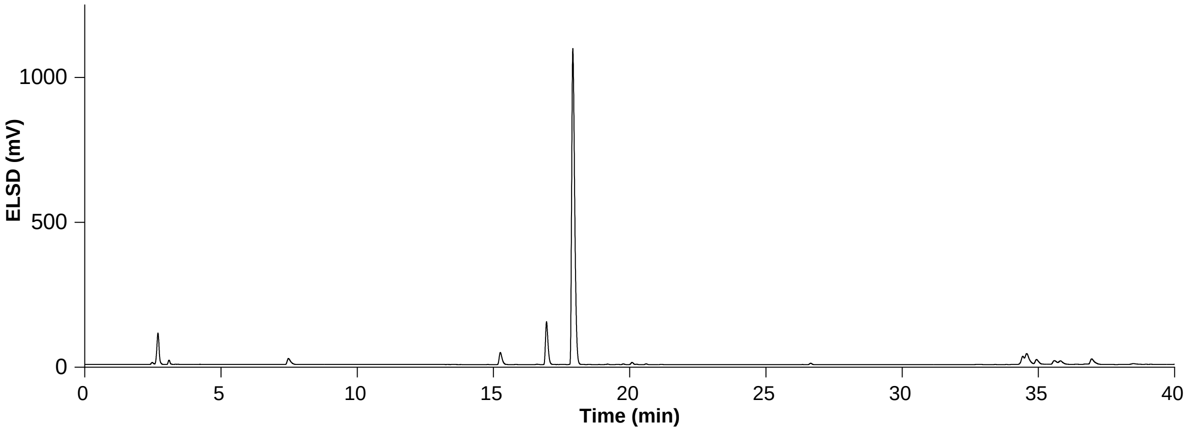

RP-HPLC of purified deprotected **15** (ELSD trace, **Method E-1**, t_R = 18.0 min):

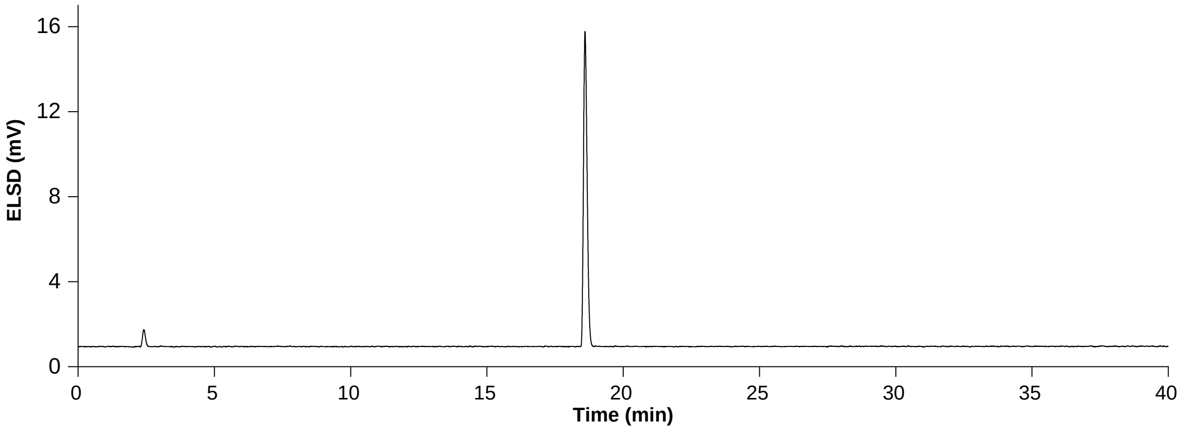

Temperature profile inside the reaction vessel during one synthesis cycle of **15**:

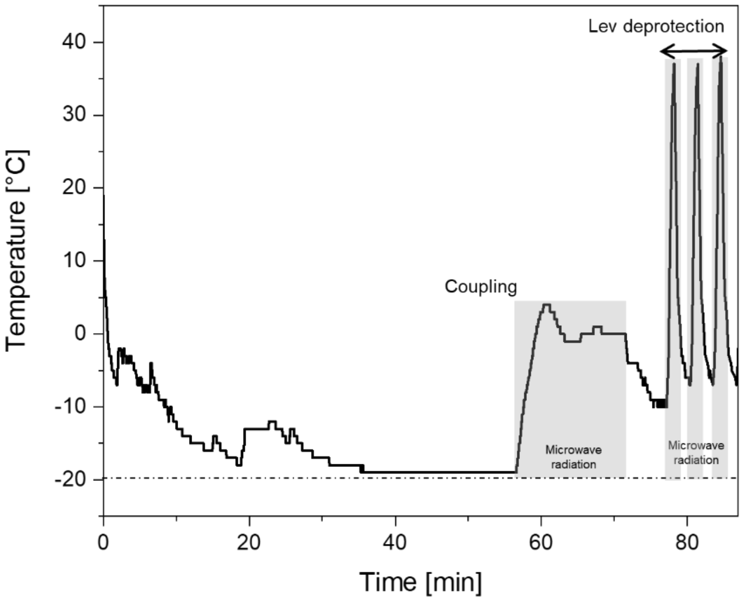

^{1}H NMR (700 MHz, D_2O) of **15**:

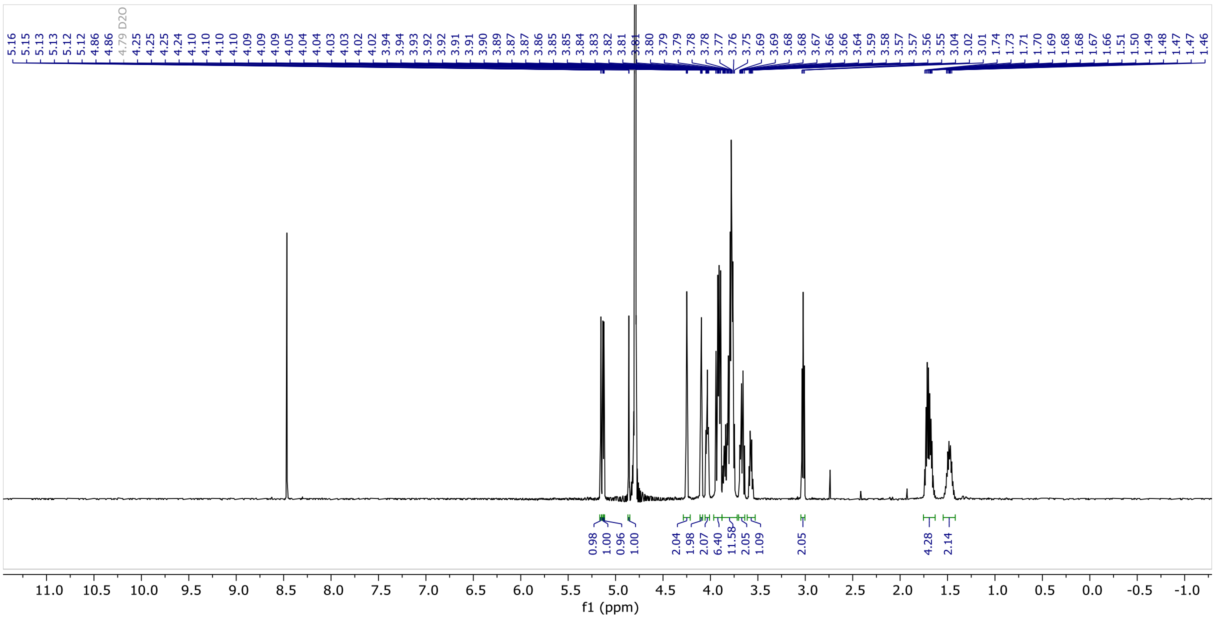

^{13}C NMR (176 MHz, D_2O) of **15**:

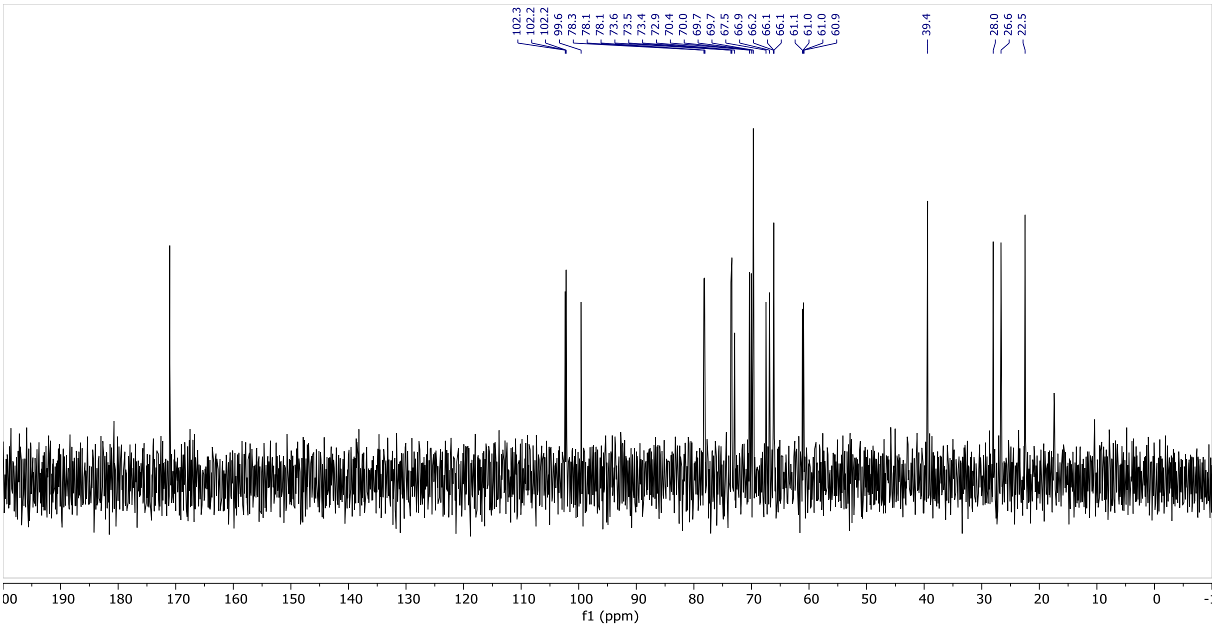

Coupled $^{13}C,^{1}H$ HSQC of **15**:

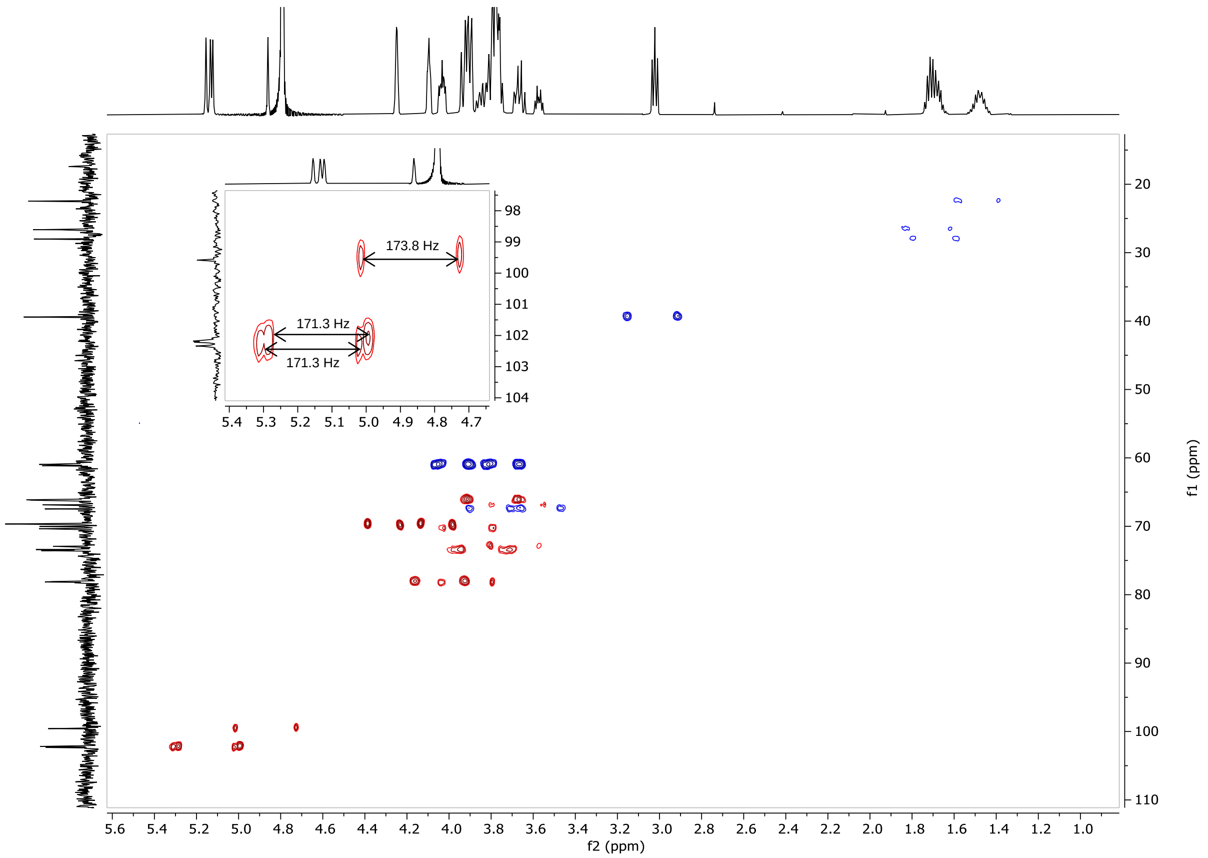

5-Amino-pentyl α-(1→6)-D-tetramannopyranoside (17)

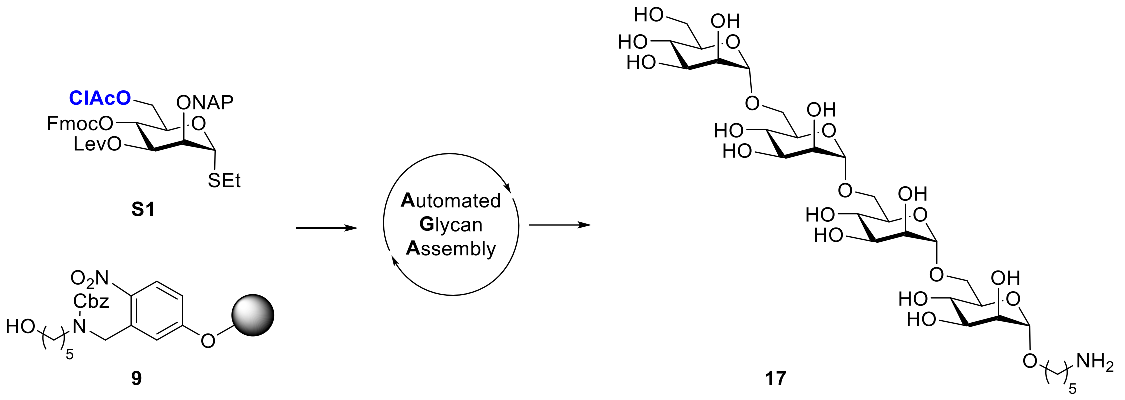

Repeat	Building Blocks	Modules	Notes	
1x		**I** – Acidic Wash		
	**S1** (2 x 6.5 equiv.)	**IIa** – Glycosylation with thioglycoside – 2 cycles	-20 °C (T_1)<br>0 °C (T_2)	10 min (t_1)<br>35 min (t_2)
		**III** – Capping<br>**IVe** – ClAc Deprotection		
3x		**I** – Acidic Wash		
	**S1** (6.5 equiv.)	**IIa** – Glycosylation with thioglycoside	-20 °C (T1)<br>0 °C (T2)	10 min (t1)<br>35 min (t2)
		**III** – Capping<br>**IVe** – ClAc Deprotection		

Protected **17** (22 mg, 0.008 mmol, crude yield: 58%) was obtained as a colorless oil after photocleavage from solid support following **Method A-1**. Deprotection of **17** following **Method C** and **D** and purification by reverse-phase HPLC (**Method E-1**, t_R = 18.8 min) afforded deprotected compound **17** (1.8 mg, 0.002 mmol, 18%) as a white solid after lyophylization.

^{1}H NMR (600 MHz, D_2O): δ 4.88 (d, *J* = 1.8 Hz, 1H), 4.86 (d, *J* = 1.9 Hz, 1H), 4.86 (d, *J* = 1.8 Hz, 1H), 4.83 (d, *J* = 1.8 Hz, 1H), 4.00 – 3.65 (m, 24H), 3.62 (t, *J* = 9.7 Hz, 1H), 3.54 (dt, *J* = 9.9, 6.1 Hz, 1H), 3.00 – 2.93 (m, 2H), 1.72 – 1.61 (m, 4H), 1.49 – 1.35 (m, 2H) ppm.

^{13}C NMR (151 MHz, D_2O): δ 99.8, 99.3, 99.2, 72.6, 70.8, 70.7, 70.6, 70.6, 70.5, 70.0, 69.9, 69.9, 67.6, 66.7, 66.5, 66.5, 66.5, 65.5, 65.5, 65.4, 60.8, 39.3, 28.0, 26.6, 22.5 ppm.

HRMS (QToF): Calcd for $C_{29}H_{53}NO_{21}$ $[M + H]^+$ 752.3183; found 752.3209.

NP-HPLC of crude protected **17** (ELSD trace, **Method B-1**, t_R = 21.9 min):

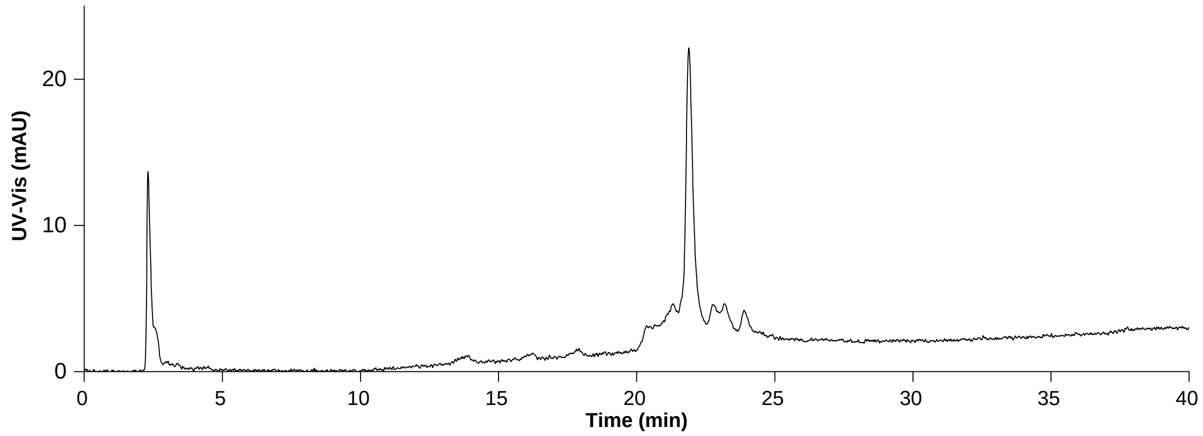

RP-HPLC of purified deprotected **17** (ELSD trace, **Method E-1**, t_R = 18.8 min):

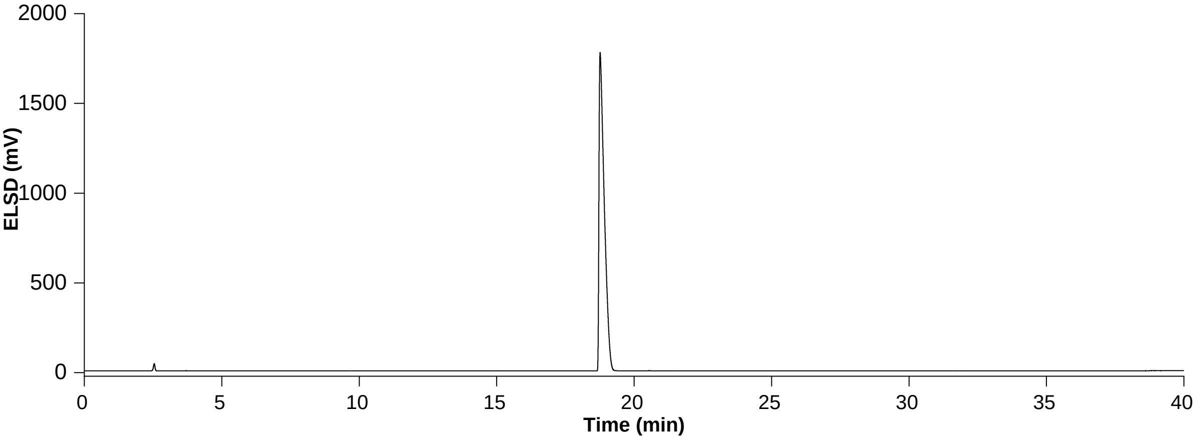

Temperature profile inside the reaction vessel during one synthesis cycle of **17**:

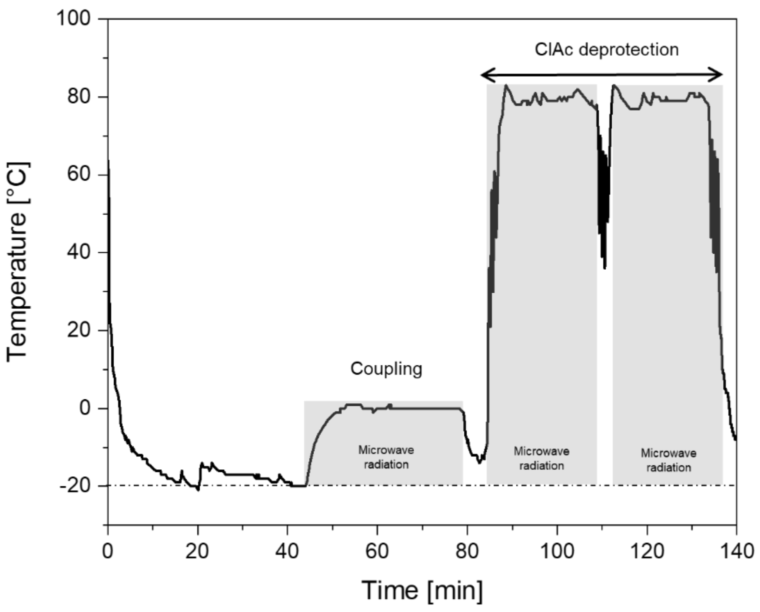

^{1}H NMR (600 MHz, D_2O) of **17**:

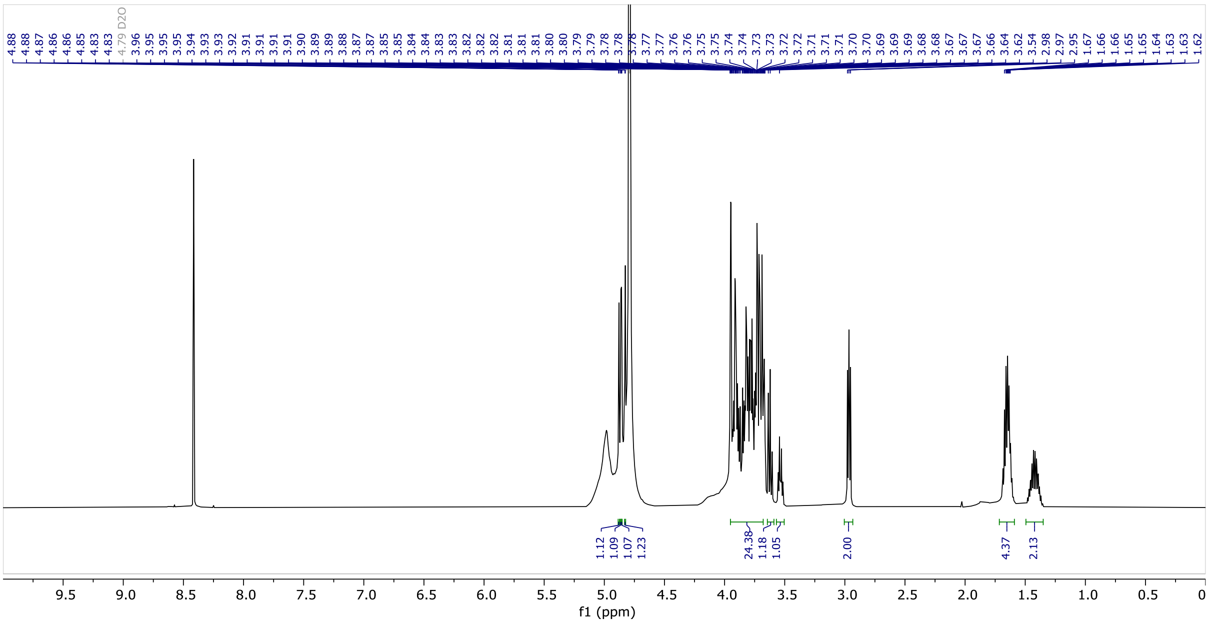

^{13}C NMR (151 MHz, D_2O) of **17**:

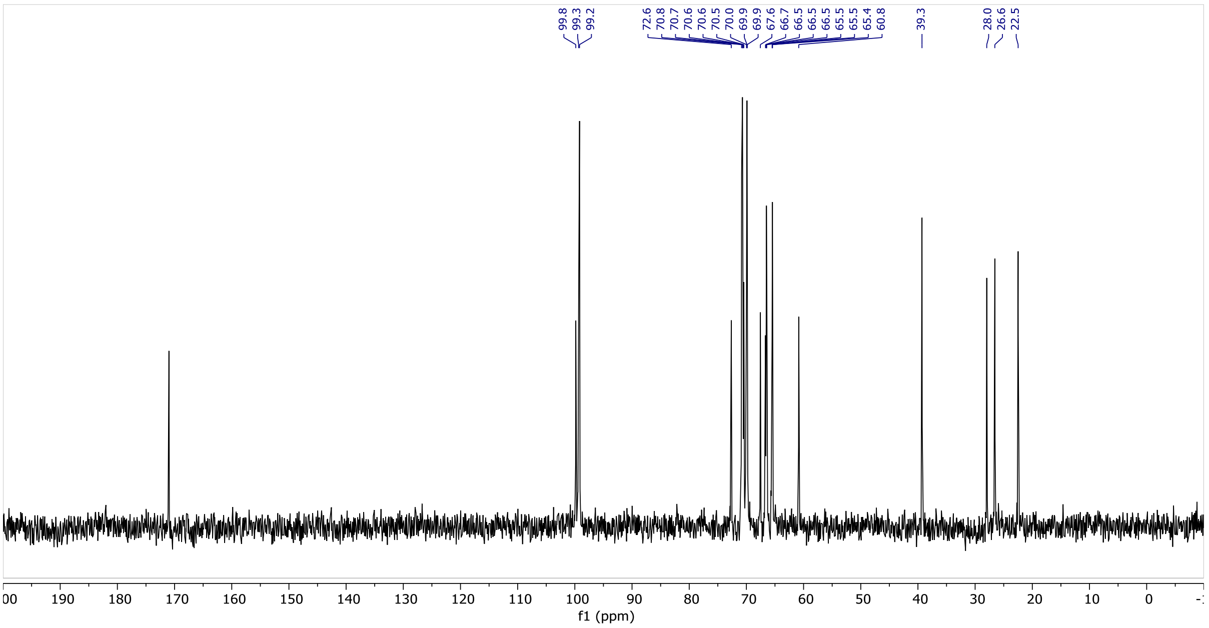

Coupled $^{13}C,^{1}H$ HSQC of **17**:

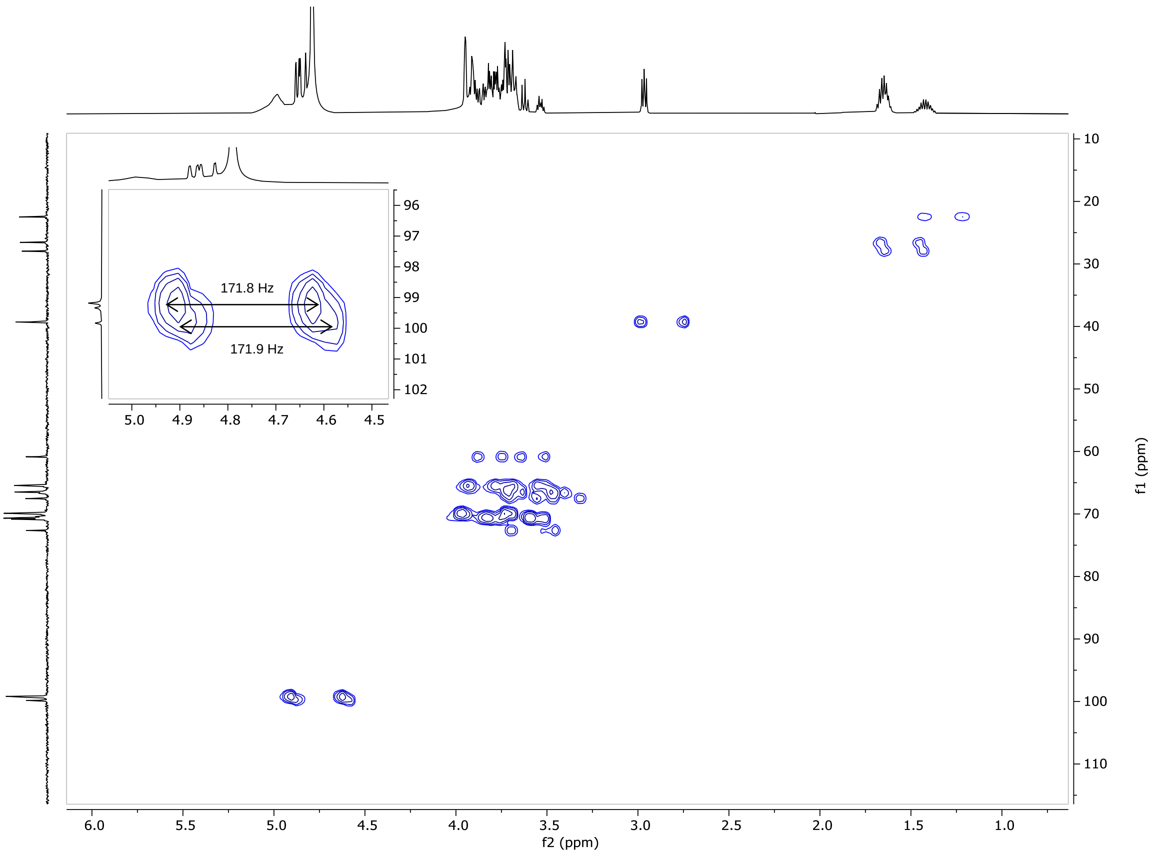

$^{13}C,^{1}H$ HSQC of **17**:

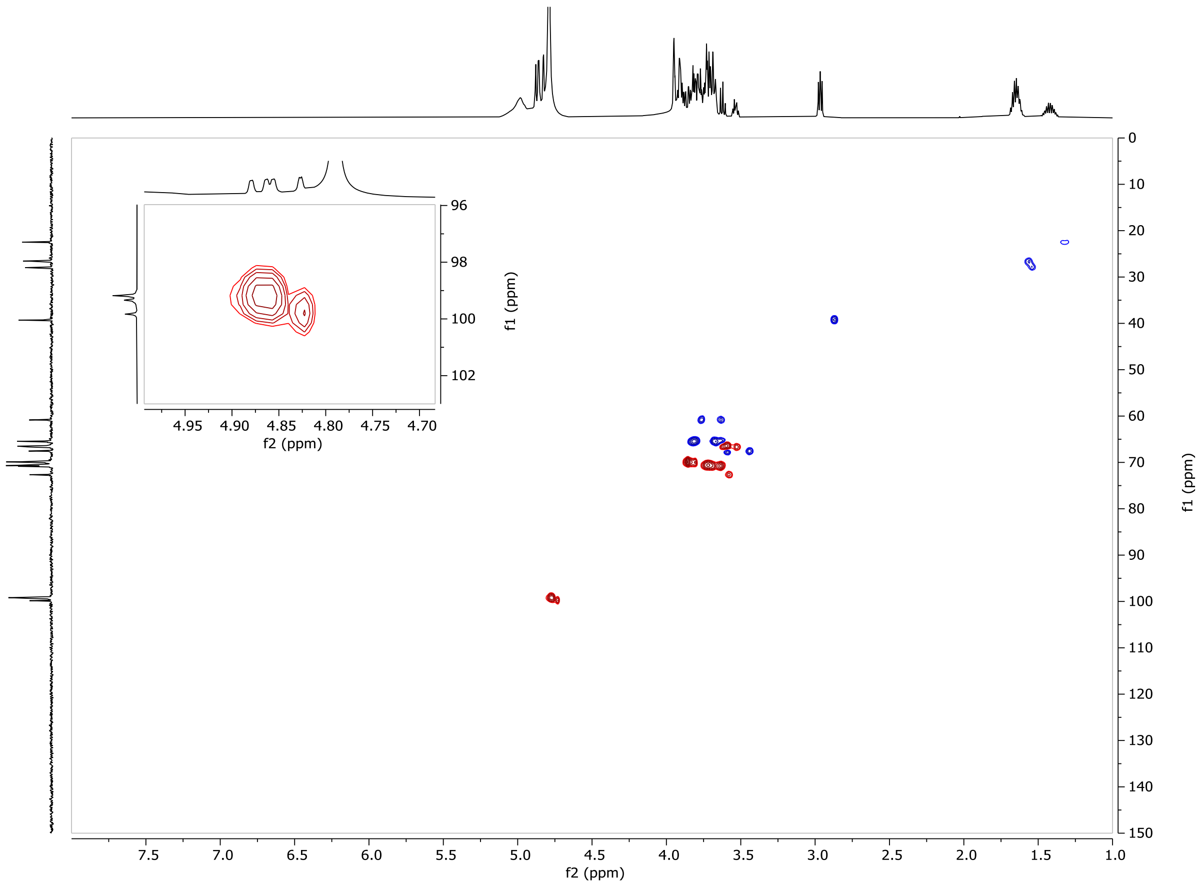

5-Amino-pentyl α-(1→2)-D-tetramannopyranoside (14)

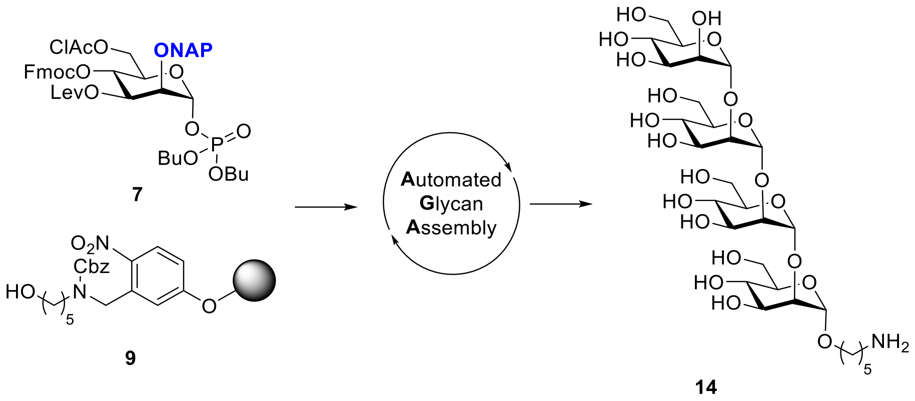

Repeat	Building Blocks	Modules	Notes	
4x		**I** – Acidic Wash		
	**7** (2 x 4.7 equiv.)	**IIb** – Glycosylation with glycosyl phosphate – 2 cycles	-20 °C (T_1)<br>0 °C (T_2)	30 min (t_1)<br>10 min (t_2)
		**III** – Capping		
		**IVd** – NAP Deprotection		

Protected **14** (17 mg, 0.007 mmol, crude yield: 51%) was obtained as a colorless oil after photocleavage from solid support following **Method A-1**. Deprotection of **14** following **Method C** and **D** and purification by reverse-phase HPLC (**Method E-1**, t_R = 15.0 min) afforded deprotected compound **14** (1.9 mg, 0.003 mmol, 19%) as a white solid after lyophylization.

^{1}H NMR (600 MHz, D_2O): δ 5.27 (d, *J* = 1.9 Hz, 1H), 5.25 (d, *J* = 1.9 Hz, 1H), 5.06 (d, *J* = 1.9 Hz, 1H), 5.01 (d, *J* = 1.9 Hz, 1H), 4.10 – 4.01 (m, 3H), 3.96 – 3.78 (m, 9H), 3.78 – 3.56 (m, 13H), 3.55 – 3.47 (m, 1H), 3.00 – 2.94 (m, 2H), 1.71 – 1.58 (m, 4H), 1.46 – 1.36 (m, 2H) ppm.

^{13}C NMR (151 MHz, D_2O): δ 102.1, 100.6, 100.5, 97.9, 85.8, 79.5, 78.8, 78.5, 73.2, 73.1, 72.7, 70.2, 70.1, 69.9, 69.8, 67.5, 67.0, 66.9, 66.9, 66.7, 61.8, 61.0, 60.9, 38.7, 27.9, 26.4, 22.4 ppm.

HRMS (QToF): Calcd for $C_{29}H_{53}NO_{21}$ $[M + H]^+$ 752.3183; found 752.3214.

RP-HPLC of crude deprotected **14** (ELSD trace, **Method E-1**, t_R = 15.0 min):

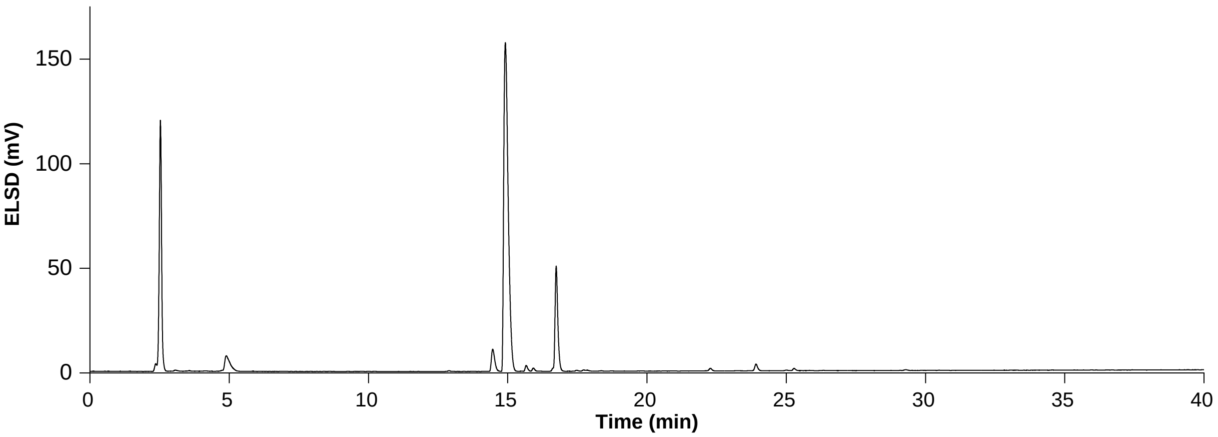

RP-HPLC of purified deprotected **14** (ELSD trace, **Method E-1**, t_R = 15.0 min):

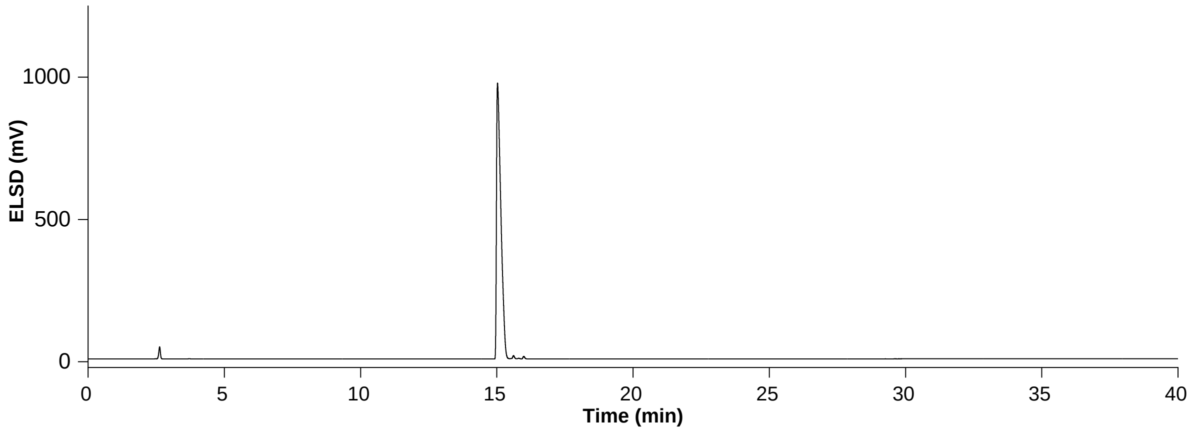

Temperature profile inside the reaction vessel during one synthesis cycle of **14**:

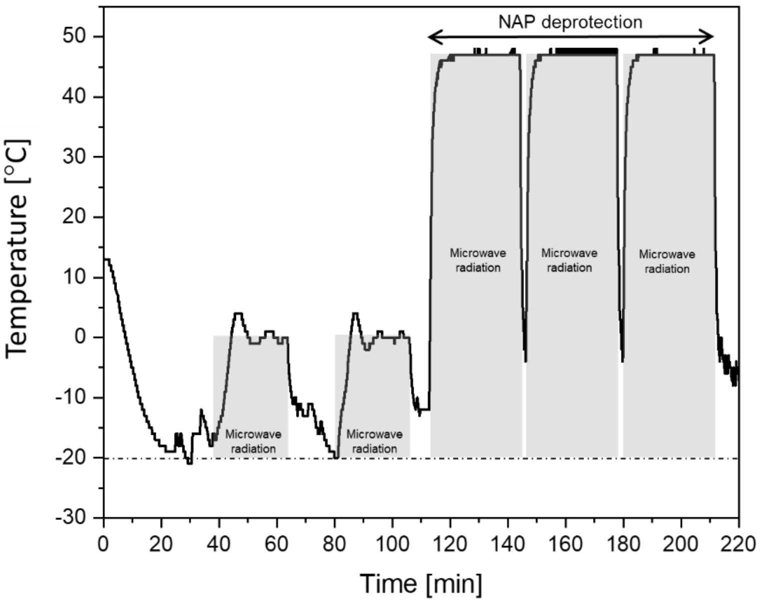

^{1}H NMR (600 MHz, D_2O) of **14**:

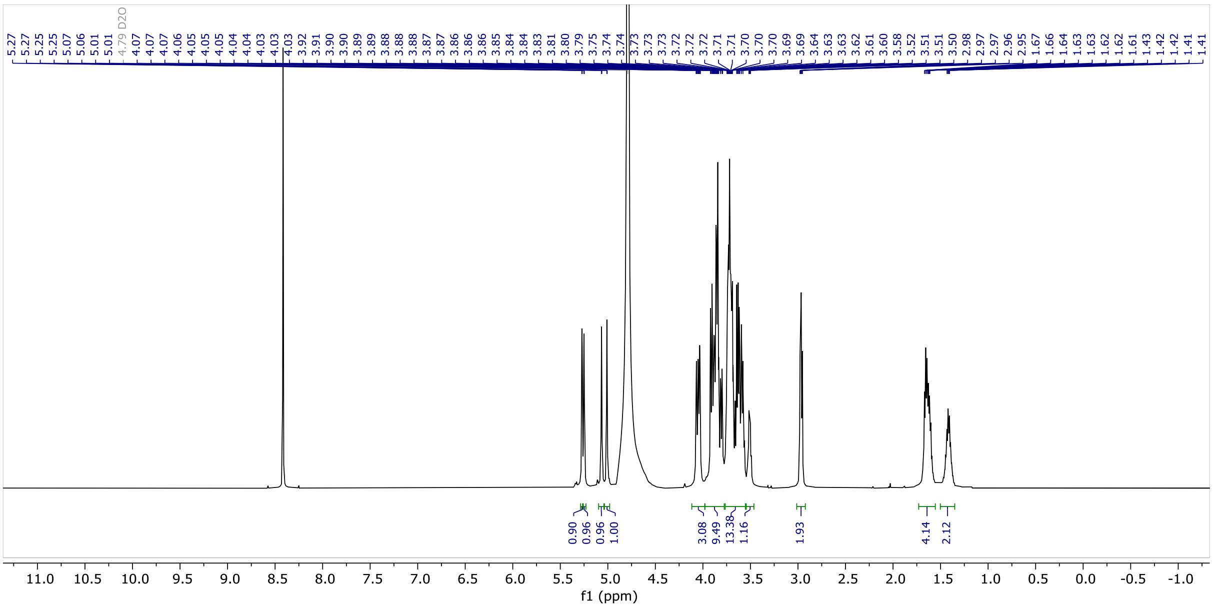

^{13}C NMR (151 MHz, D_2O) of **14**:

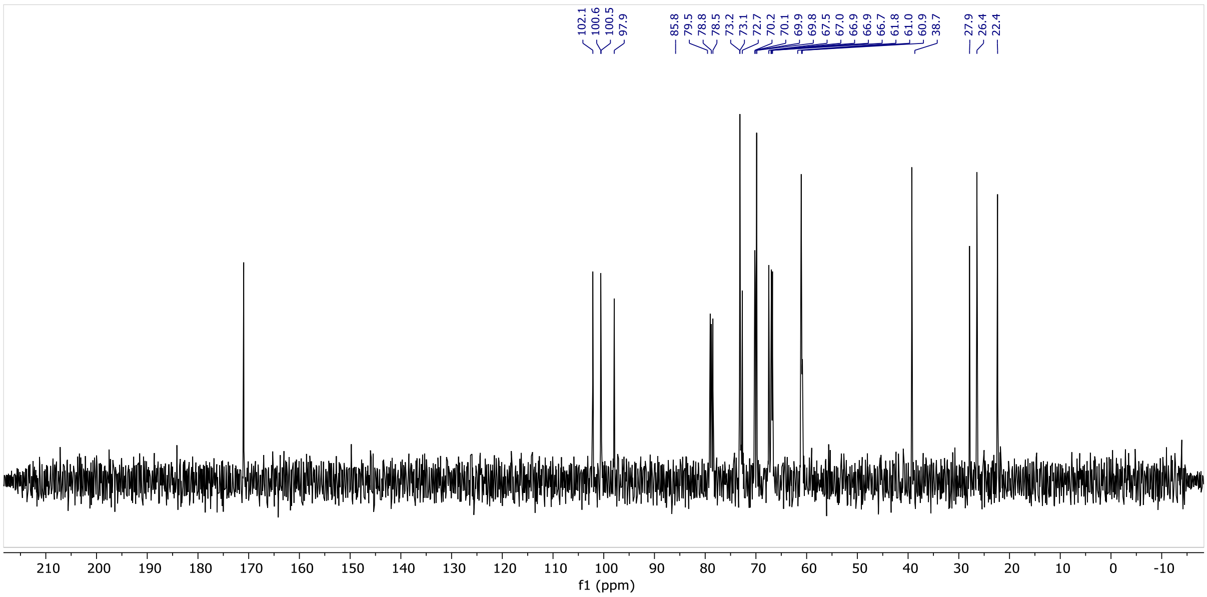

Coupled $^{13}C,^{1}H$ HSQC of **14**:

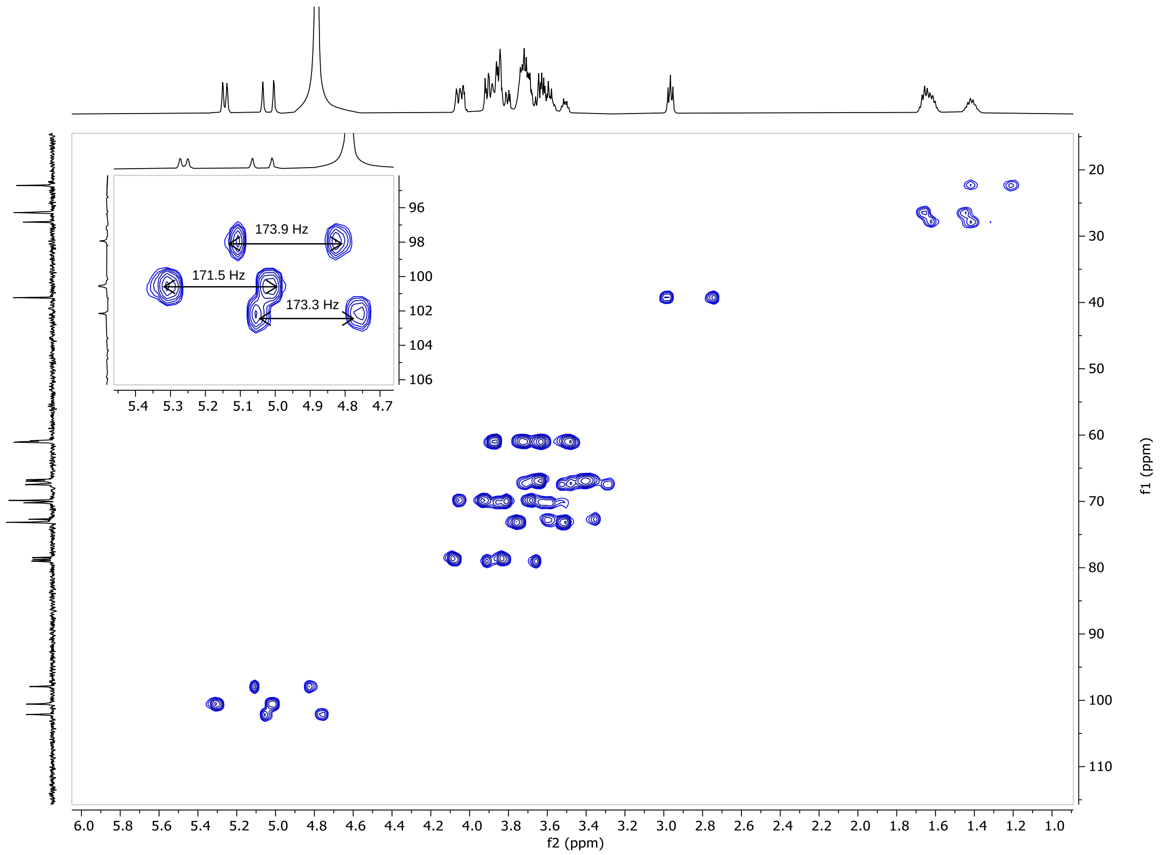

$^{13}C,^{1}H$ HSQC of **14**:

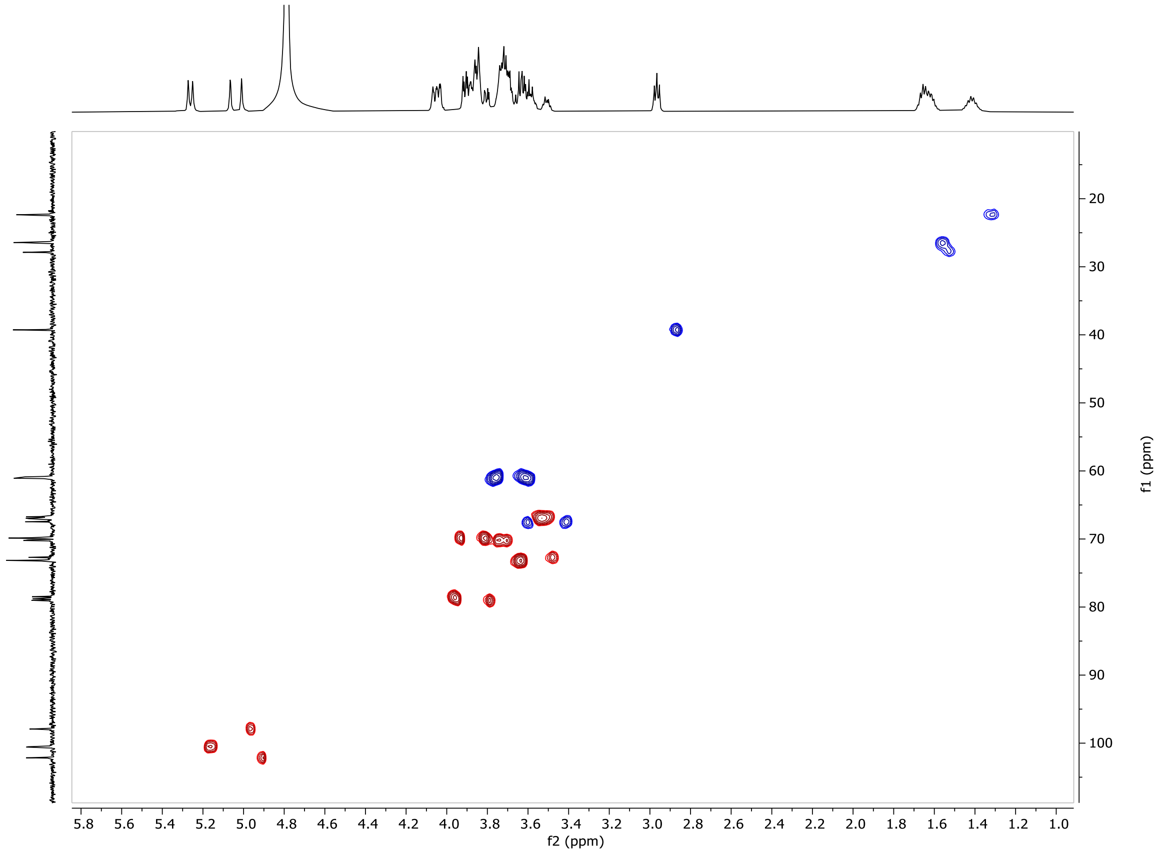

5.5.2 Non-Linear On-Resin Deprotected Trimer (18)

α-D-Mannopyranosyl-(1→2)-α-D-mannopyranosyl-(1→6)-α/β-D-mannopyranoside (18)

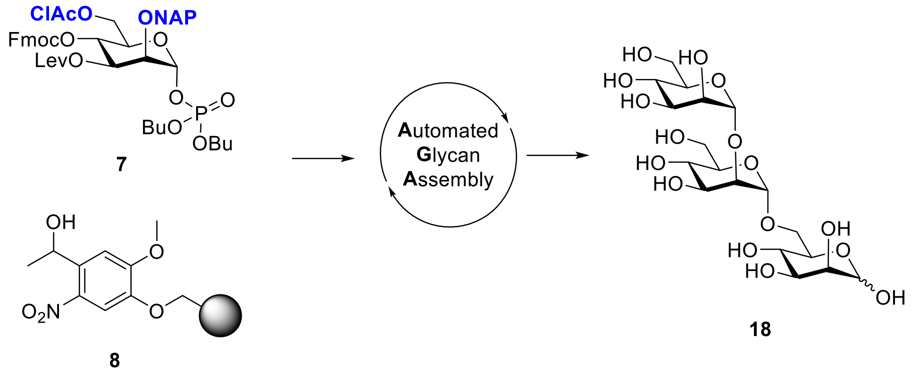

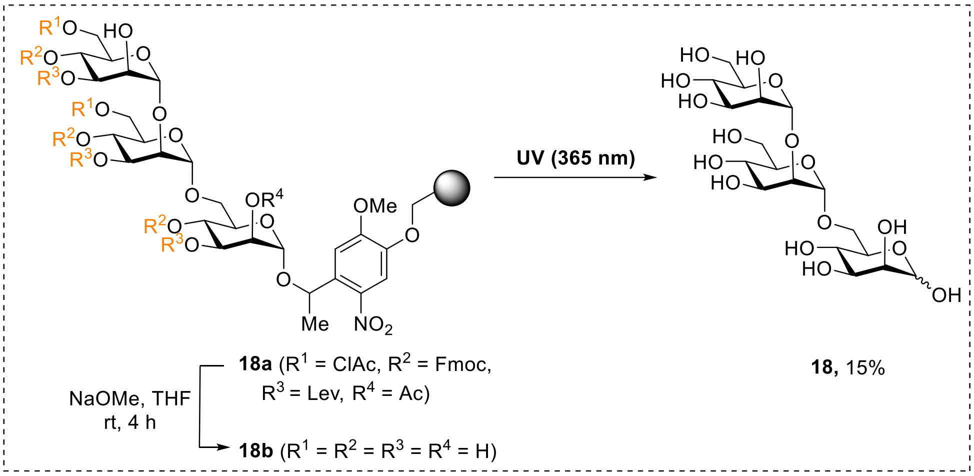

Repeat	Building Blocks	Modules	Notes
**1x**		**I** – Acidic Wash	
	**7** (2 x 4.7 equiv.)	**IIb** – Glycosylation with glycosyl phosphate – 2 cycles	-20 °C (T_1) 30 min (t_1)<br>0 °C (T_2) 10 min (t_2)
		**IVd** – NAP Deprotection<br>**III** – Capping<br>**IVe** – ClAc Deprotection	
**1x**		**I** – Acidic Wash	
	**7** (4.7 equiv.)	**IIb** – Glycosylation with glycosyl phosphate	-20 °C (T_1) 30 min (t_1)<br>0 °C (T_2) 10 min (t_2)
		**III** – Capping<br>**IVd** – NAP Deprotection	
**1x**		**I** – Acidic Wash	
	**7** (2 x 4.7 equiv.)	**IIb** – Glycosylation with glycosyl phosphate – 2 cycles	-20 °C (T_1) 30 min (t_1)<br>0 °C (T_2) 10 min (t_2)
		**III** – Capping<br>**IVd** – NAP Deprotection<br>**VI** - Methanolysis	

Photocleavage from solid support using **Method A-2** followed by purification by reverse-phase HPLC (**Method E-2**, t_R = 21.6 min) afforded deprotected compound **18** (1.2 mg, 0.002 mmol, 15%) as a white solid after lyophylization.

^{1}H NMR (700 MHz, D_2O, α-isomer): δ 5.17 (d, *J* = 1.9 Hz, 1H), 5.14 (d, *J* = 1.9 Hz, 1H), 5.05 (d, *J* = 2.2 Hz, 1H), 4.08 (dd, *J* = 3.4, 1.8 Hz, 1H), 4.03 (dd, *J* = 3.5, 1.8 Hz, 1H), 4.00 – 3.69 (m, 14H), 3.66 – 3.62 (m, 2H) ppm.

^{13}C NMR (176 MHz, D_2O, α-isomer): δ 102.3, 98.7, 94.2, 78.5, 73.2, 72.7, 71.1, 70.7, 70.6, 70.4, 70.3, 70.2, 69.9, 66.9, 66.7, 66.0, 61.1, 60.9 ppm.

HRMS (QToF): Calcd for $C_{18}H_{32}O_{16}Na$ $[M + Na]^+$ 527.1583; found 527.1586.

RP-HPLC of crude deprotected **18** (ELSD trace, **Method E-2**, t_R = 21.6 min):

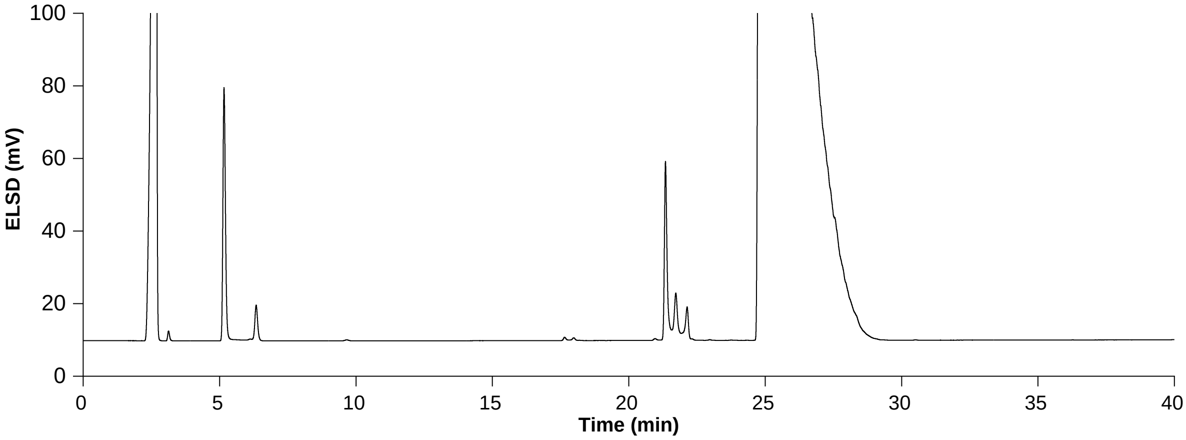

RP-HPLC of purified deprotected **18** (ELSD trace, **Method E-2**, t_R = 21.6 min):

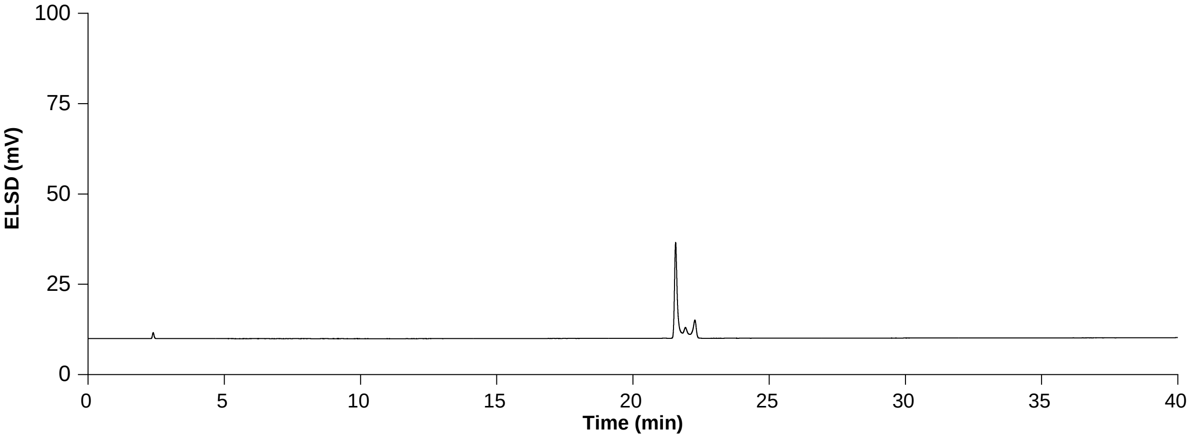

Temperature profile inside the reaction vessel during the synthesis of **18**:

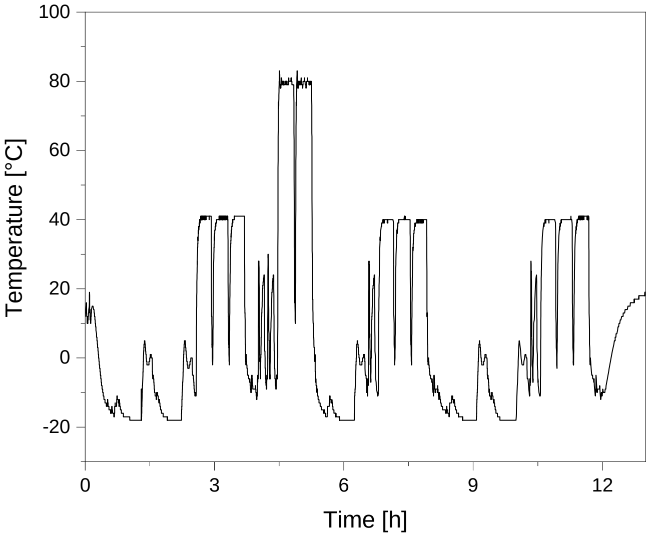

^{1}H NMR (700 MHz, D_2O) of **18**:

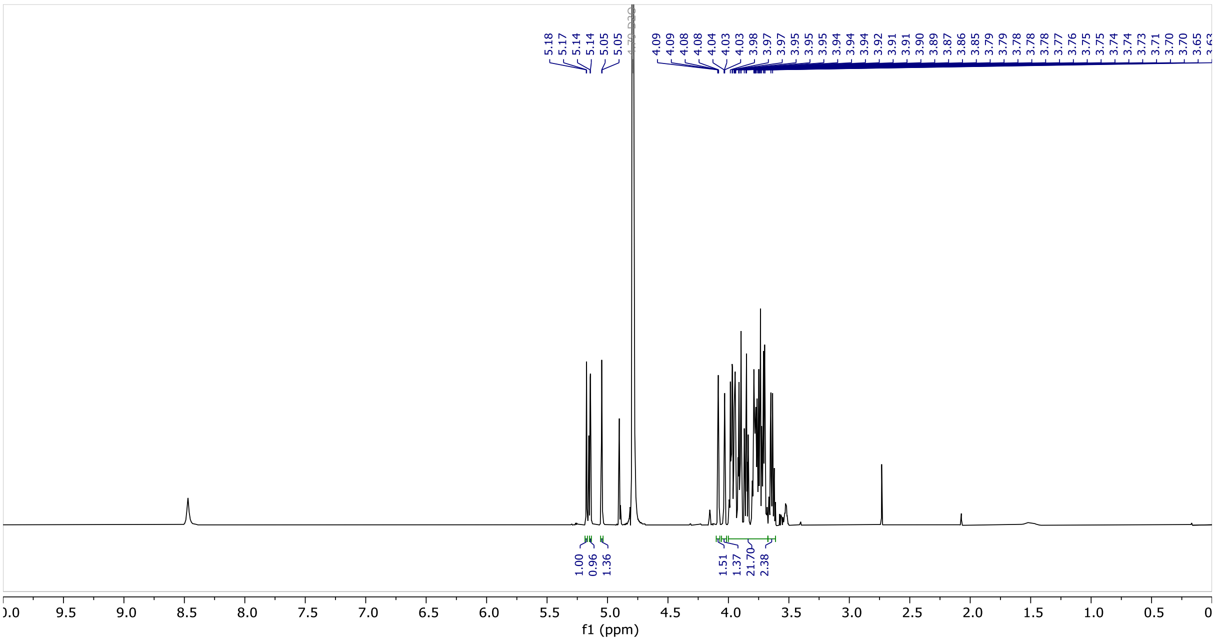

^{13}C NMR (151 MHz, D_2O) of **18**:

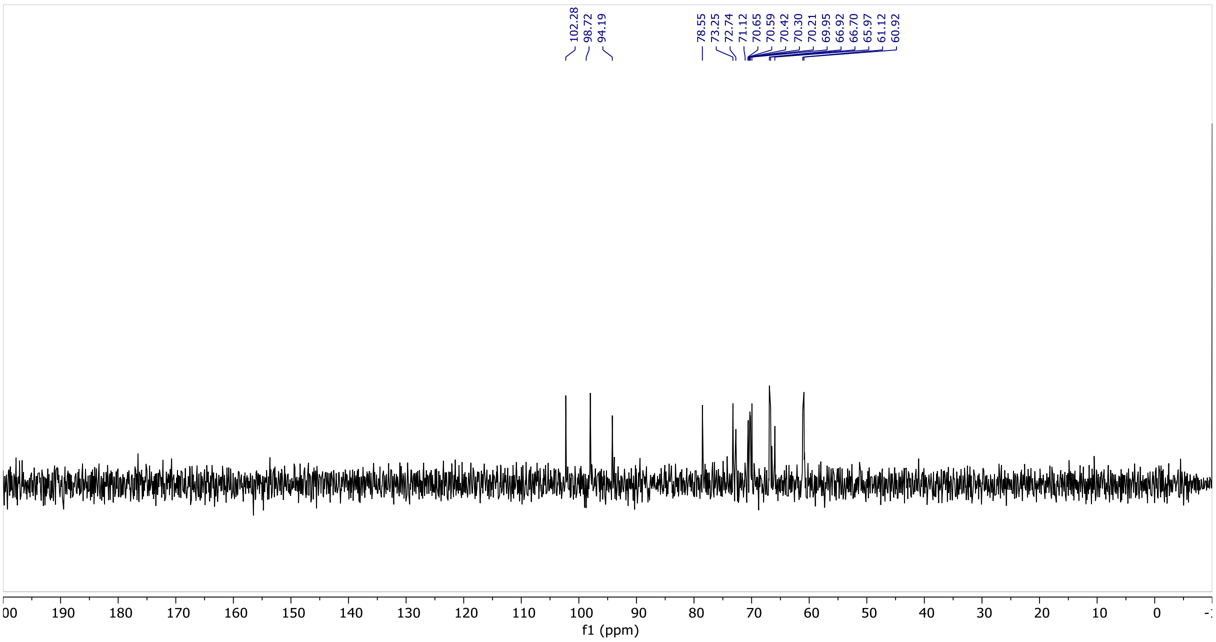

Coupled ^{13}C,^{1}H HSQC of **18**:

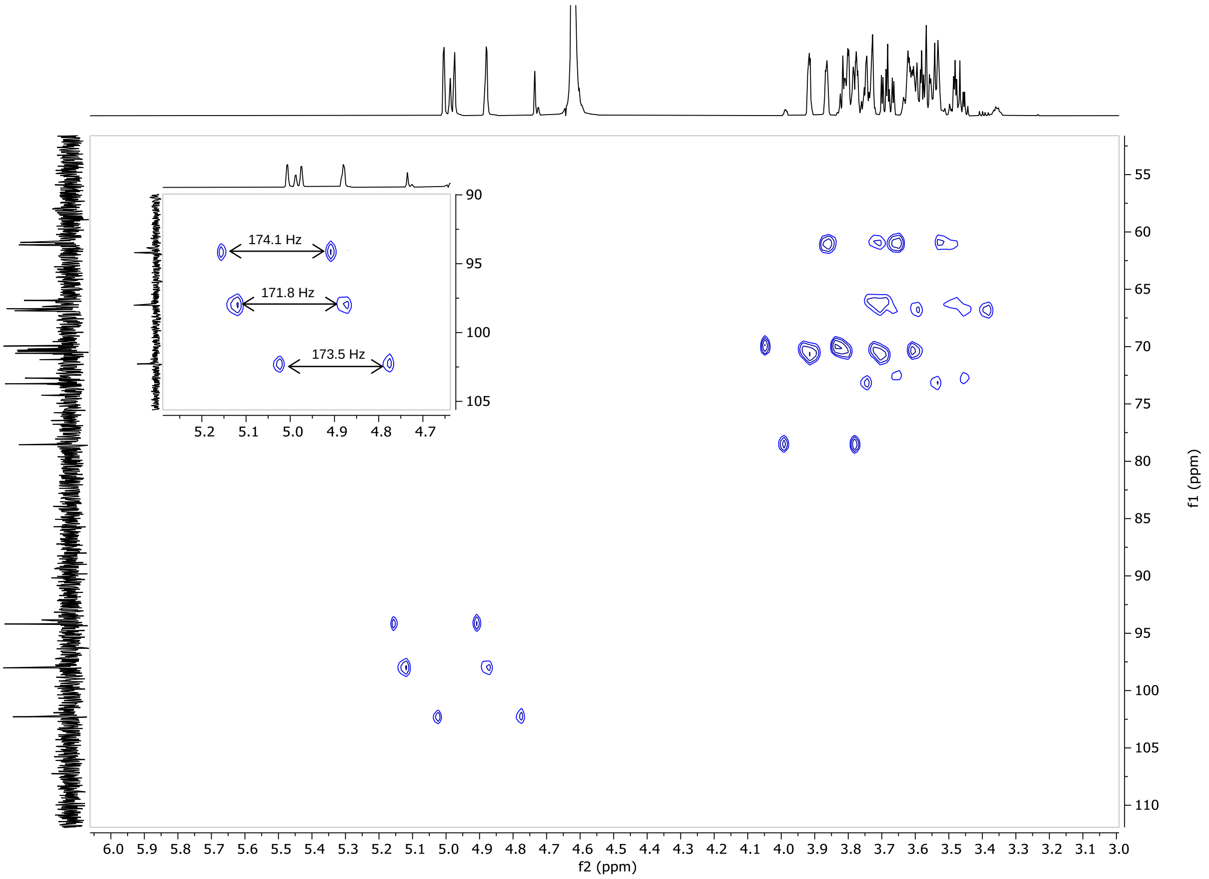

$^{13}C,^{1}H$ HSQC of **18**:

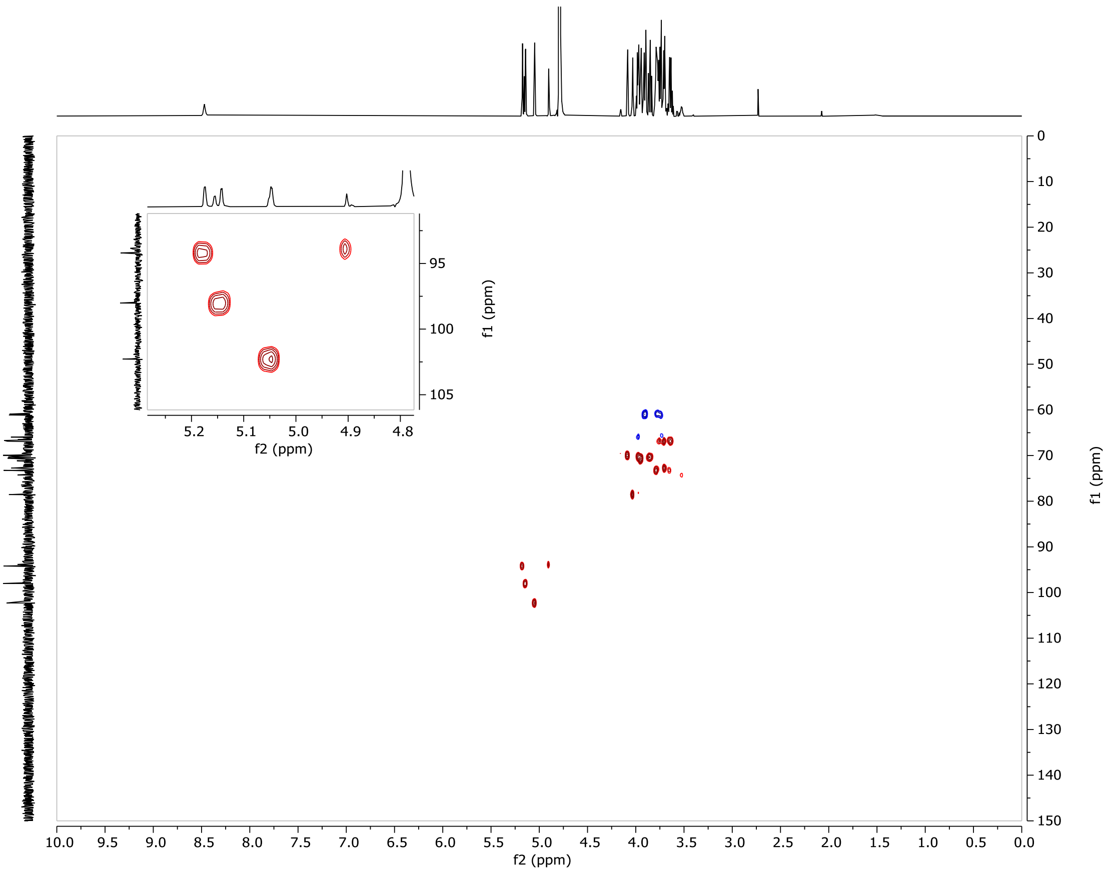

FT-IR spectrum[1] of the resin containing **18** before and after **Module VI**:

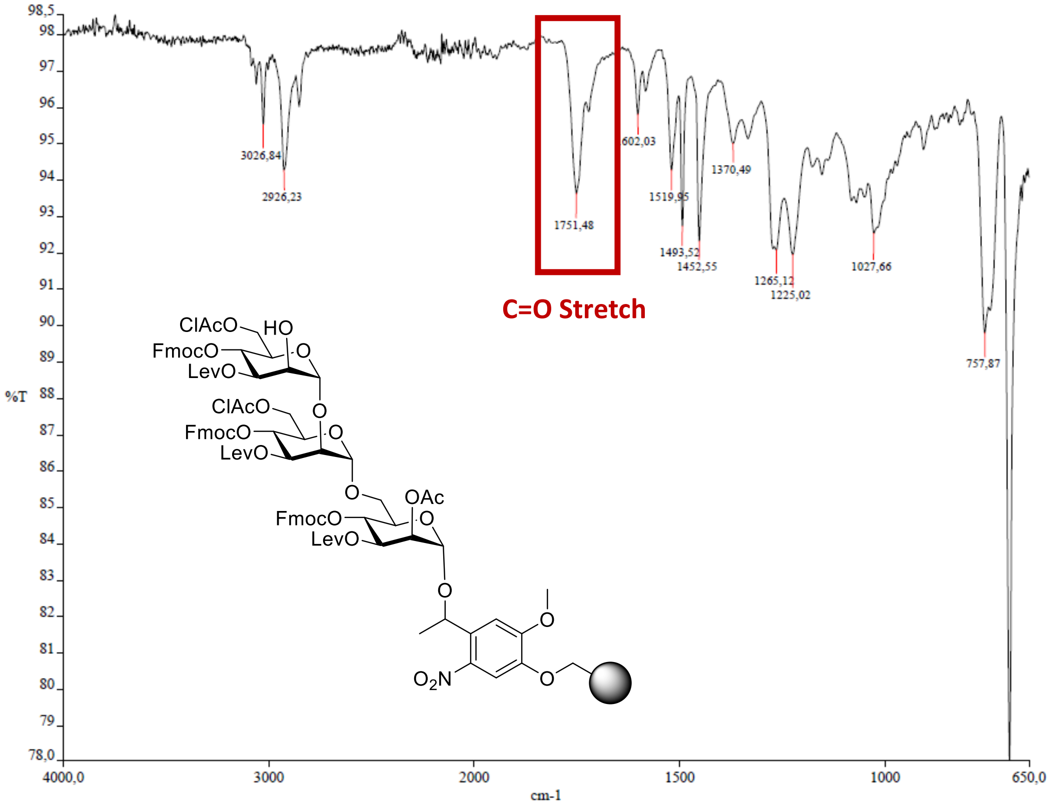

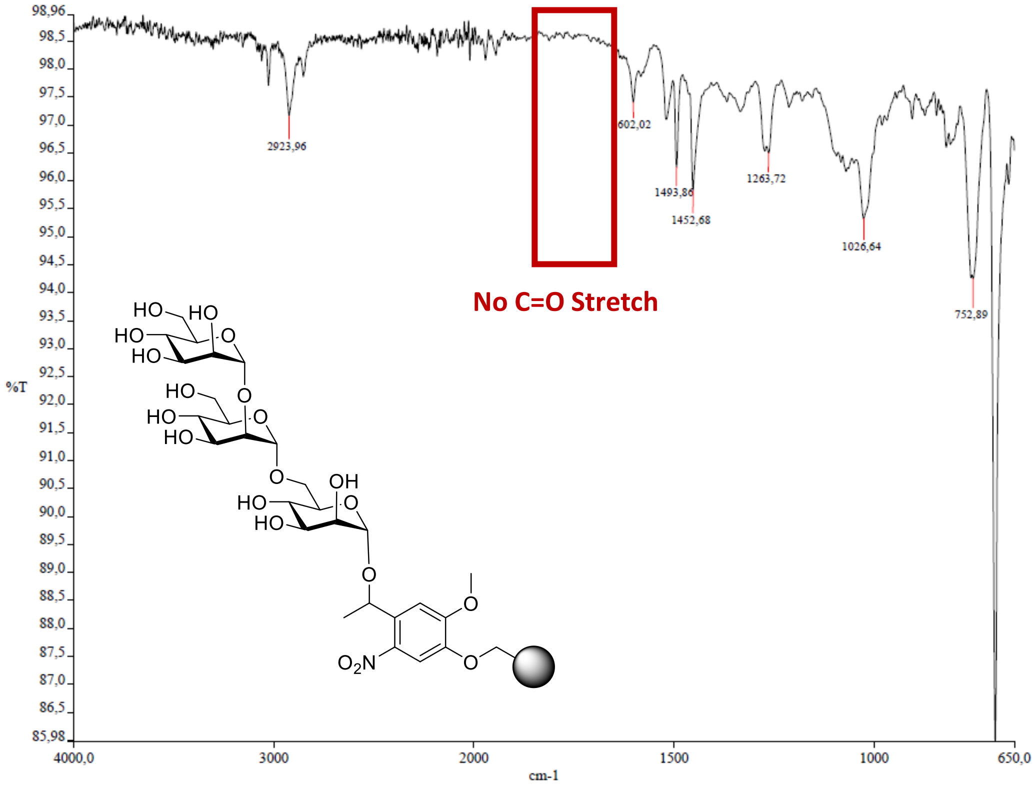

[1] 20-30 beads of resin were ground using a mortar. The ground beads were suspended in CH_2Cl_2 and applied on a Perkin-Elmer 1600 FTIR spectrometer. Spectra were taken after evaporation of the CH_2Cl_2.

5.5.3 Double Branched Trimer (S2)

6-*O*-Acetyl-2-*O*-benzoyl-3,4-di-*O*-benzyl-α-D-mannopyranosyl-(1→3)-[6-*O*-acetyl-2-*O*-benzoyl-3,4-di-*O*-benzyl-α-D-mannopyranosyl-(1→6)]-2-*O*-acetyl-2-*O*-(2-naphthalenylmethyl)-α/β-D-mannopyranoside (S2)

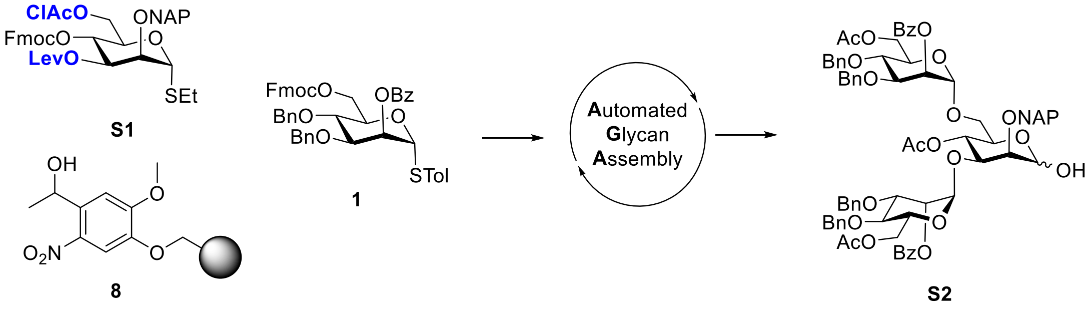

Repeat	Building Blocks	Modules	Notes
1x		**I** – Acidic Wash	
	**S1** (2 x 6.5 equiv.)	**IIa** – Glycosylation with thioglycoside – 2 cycles	-20 °C (T_1) 10 min (t_1)<br>0 °C (T_2) 35 min (t_2)
		**IVc** – Fmoc Deprotection<br>**III** – Capping<br>**IVb** – Lev Deprotection<br>**IVe** – ClAc Deprotection	
1x		**I** – Acidic Wash	
	**1** (3 x 6.5 equiv.)	**IIa** – Glycosylation with thioglycoside – 3 cycles	-20 °C (T_1) 10 min (t_1)<br>-10 °C (T_2) 25 min (t_2)
		**IVa** – Fmoc Deprotection (6 cycles)<br>**III** – Capping (4 cycles)	

Photocleavage from solid support using **Method A-1** followed by purification by normal-phase HPLC (**Method B-3**, t_R = 22.7 min) afforded compound **S2** (14 mg, 0.011 mmol, **65%**) as a colorless oil.

^{1}H NMR (400 MHz, $CDCl_3$): δ 8.09 – 8.01 (m, 5H), 7.75 – 7.56 (m, 7H), 7.52 – 7.39 (m, 6H), 7.36 – 7.16 (m, 19H), 5.60 (s, 1H), 5.48 (s, 1H), 5.39 (t, *J* = 10.0 Hz, 1H), 5.24 (s, 1H), 5.11 (s, 1H), 5.01 – 4.67 (m, 7H), 4.65 – 4.42 (m, 6H), 4.37 – 3.95 (m, 8H), 3.92 – 3.78 (m, 5H), 2.18 (s, 3H), 2.05 (s, 3H), 1.91 (s, 3H) ppm.

^{13}C NMR (101 MHz, $CDCl_3$): δ 171.2, 170.8, 170.4, 165.8, 165.6, 138.2, 138.0, 137.7, 135.2, 133.5, 133.5, 133.2, 133.1, 130.0, 130.0, 128.7, 128.6, 128.6, 128.5, 128.5, 128.5, 128.2, 128.0, 127.9, 127.8, 127.8, 127.0, 126.3, 126.1, 125.8, 99.5, 97.6, 92.7, 78.2, 77.8, 77.4, 76.0, 75.3, 75.2, 73.8, 73.7, 73.2, 71.6, 71.5, 70.5, 70.1, 70.0, 69.3, 69.0, 68.9, 68.0, 63.4, 63.3, 21.1, 21.0, 20.7 ppm.

HRMS (QToF): Calcd for $C_{77}H_{78}O_{21}Na$ [M + Na]$^+$ 1361.4928; found 1361.4954.

NP-HPLC of crude **S2** (ELSD trace, **Method B-2**, t_R = 22.7 min):

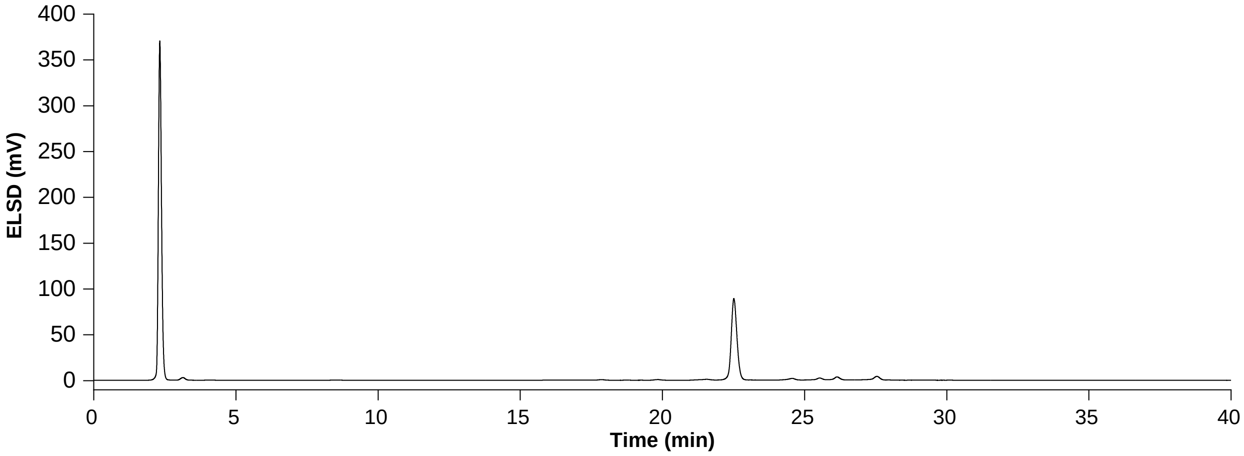

NP-HPLC of purified **S2** (ELSD trace, **Method B-2** t_R = 22.7 min):

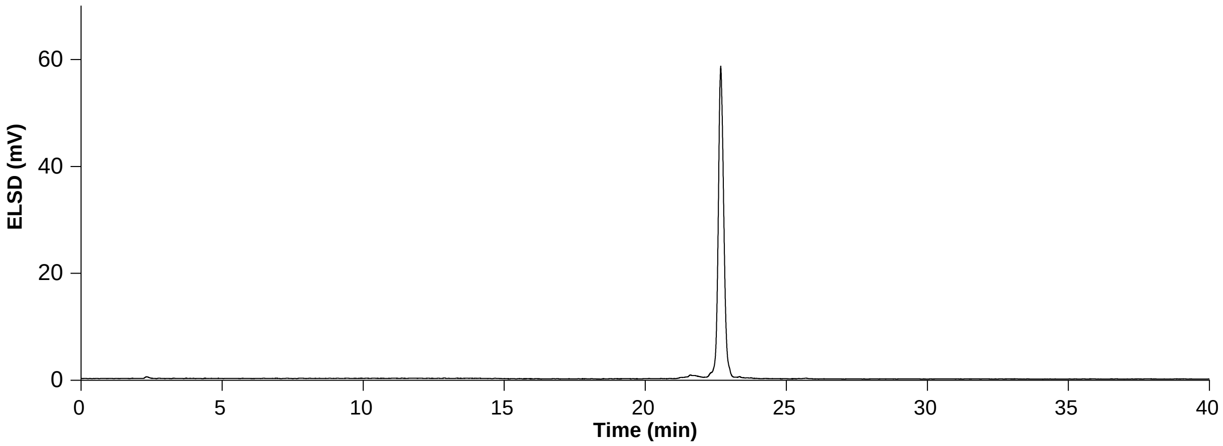

Temperature profile inside the reaction vessel during one synthesis cycle of **S2**:

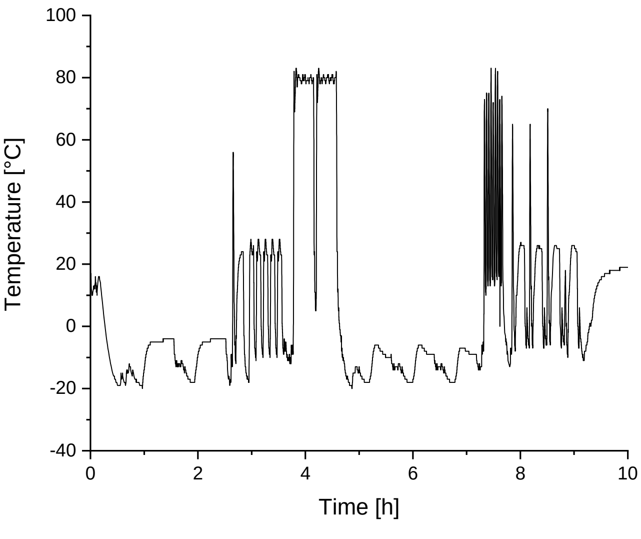

^{1}H NMR (400 MHz, $CDCl_3$) of **S2:**

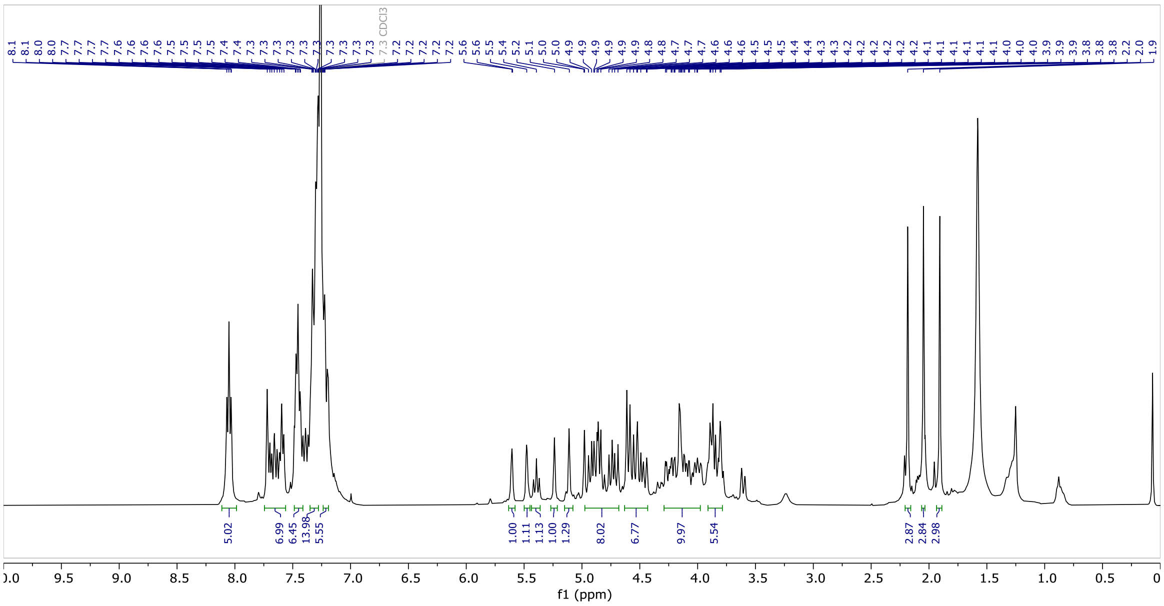

^{13}C NMR (101 MHz, $CDCl_3$) of **S2**:

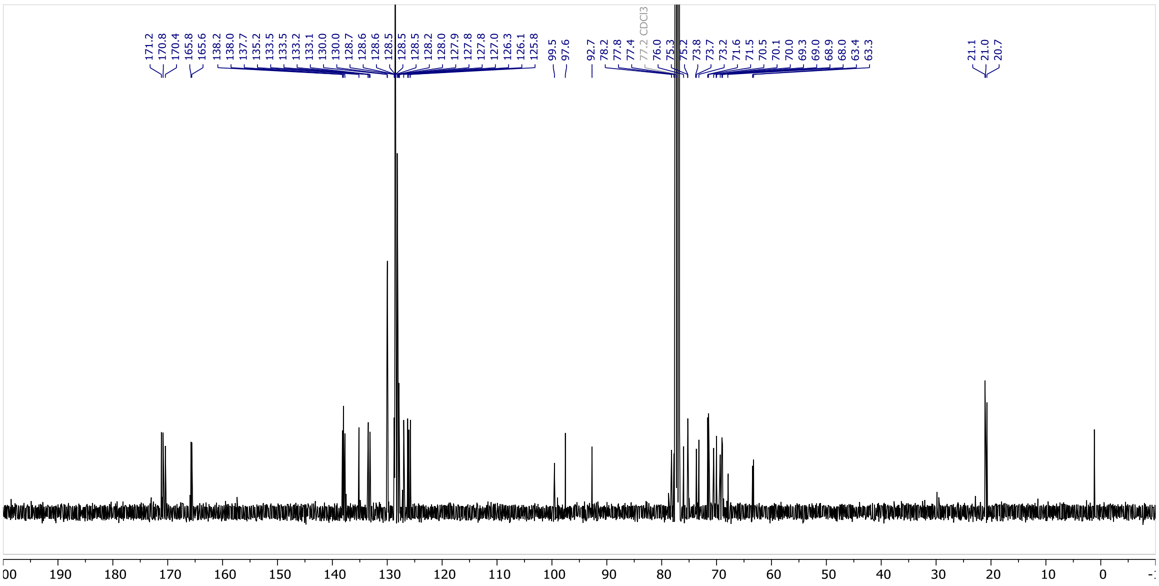

Coupled ^{13}C,^{1}H HSQC of **S2**:

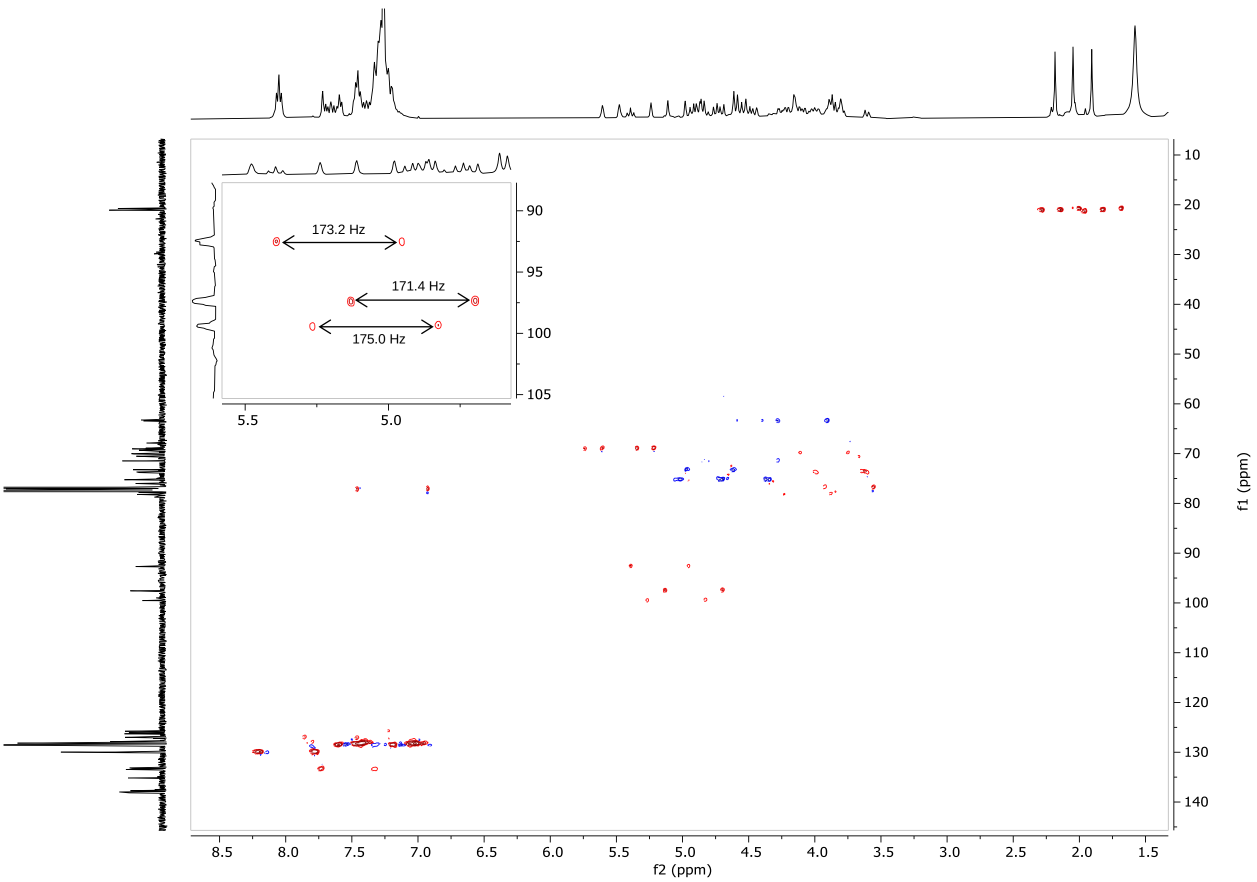
173.2 Hz
171.4 Hz
175.0 Hz
f2 (ppm)
f1 (ppm)

5.5.4 Bisecting GlcNAc Tetramer (Triple-Branched Mannose 19)

6-*O*-Acetyl-2-*O*-benzoyl-3,4-di-*O*-benzyl-α-D-mannopyranosyl-(1→3)-[4-*O*-acetyl-3,6-di-*O*-benzyl-2-deoxy-2-*N*-trichloroacetyl-β-D-glucopyranosyl-(1→4)]-[6-*O*-acetyl-2-*O*-benzoyl-3,4-di-*O*-benzyl-α-D-mannopyranosyl-(1→6)]-2-*O*-(2-naphthalenylmethyl)-α/β-D-mannopyranoside (19)

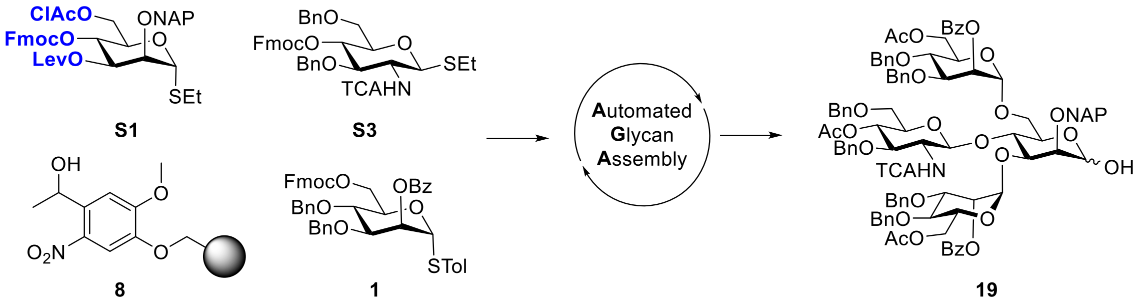

Repeat	Building Blocks	Modules	Notes
1x		**I** – Acidic Wash	
	**S1** (2 x 6.5 equiv.)	**IIa** – Glycosylation with thioglycoside – 2 cycles	-20 °C (T_1) 10 min (t_1)<br>0 °C (T_2) 35 min (t_2)
		**III** – Capping<br>**IVc** – Fmoc Deprotection	
1x		**I** – Acidic Wash	
	**S3** (6.5 equiv.)	**IIa** – Glycosylation with thioglycoside – 2 cycles	-20 °C (T_1) 10 min (t_1)<br>-10 °C (T_2) 45 min (t_2)
		**III** – Capping<br>**IVb** – Lev Deprotection<br>**IVe** – ClAc Deprotection	
1x		**I** – Acidic Wash	
	**1** (3 x 6.5 equiv.)	**IIa** – Glycosylation with thioglycoside – 3 cycles	-20 °C (T_1) 10 min (t_1)<br>-10 °C (T_2) 25 min (t_2)
		**IVa** – Fmoc Deprotection (6 cycles)<br>**III** – Capping (4 cycles)	

Photocleavage from solid support using **Method A-1** followed by purification by normal-phase HPLC (**Method B-3**, t_R = 21.6 min) afforded compound **19** (8 mg, 0.004 mmol, **28%**) as a colorless oil.

^{1}H NMR (600 MHz, $CDCl_3$, α-isomer): δ 8.11 – 8.06 (m, 4H), 7.65 – 7.56 (m, 5H), 7.47 – 7.39 (m, 7H), 7.36 – 7.11 (m, 31H), 5.86 (s, 1H), 5.74 – 5.70 (m, 1H), 5.39 (d, *J* = 2.0 Hz, 1H), 5.34 (t, *J* = 9.4 Hz, 1H), 5.16 – 5.06 (m, 3H), 4.94 (d, *J* = 10.7 Hz, 1H), 4.87 (d, *J* = 11.2 Hz, 1H), 4.81 – 4.70 (m, 5H), 4.65 – 4.58 (m, 6H), 4.55 – 4.49 (m, 4H), 4.41 (dd, *J* = 11.4, 5.0 Hz, 2H),

4.37 – 4.31 (m, 3H), 4.25 – 4.09 (m, 7H), 3.92 – 3.85 (m, 6H), 2.06 (s, 3H), 1.91 (s, 3H), 1.80 (s, 3H).

^{13}C NMR (176 MHz, $CDCl_3$, α-isomer): δ 169.9, 169.6, 169.2, 165.0, 164.5, 137.1, 136.9, 136.9, 136.6, 134.1, 132.1, 132.0, 128.9, 128.9, 128.9, 128.8, 128.8, 127.6, 127.6, 127.4, 127.4, 127.4, 127.4, 127.3, 127.3, 127.3, 127.2, 127.1, 127.0, 127.0, 126.9, 126.9, 126.9, 126.8, 126.8, 126.7, 126.7, 126.6, 98.4, 97.6, 96.5, 96.5, 91.6, 77.1, 76.7, 74.9, 74.1, 72.7, 72.6, 72.1, 70.5, 70.3, 69.4, 69.0, 68.9, 68.2, 68.0, 67.9, 66.8, 62.3, 62.2, 19.9, 19.8, 19.6 ppm.

HRMS (QToF): Calcd for $C_{99}H_{100}Cl_3NO_{26}$ $[M + Na]^+$ 1846.5491; found 1846.5565.

NP-HPLC of purified **19** (ELSD trace, **Method B-2** t_R = 25.5 min):

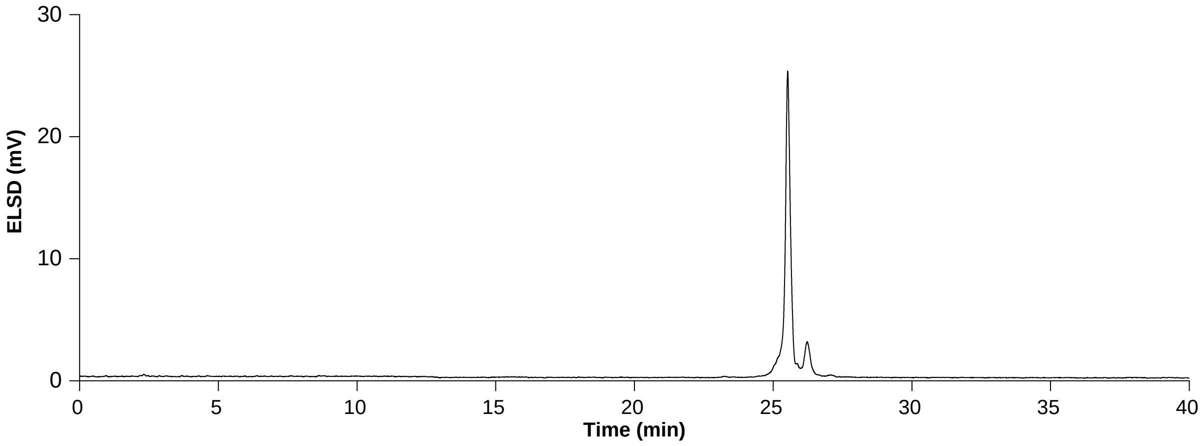

Temperature profile inside the reaction vessel during one synthesis cycle of **19**:

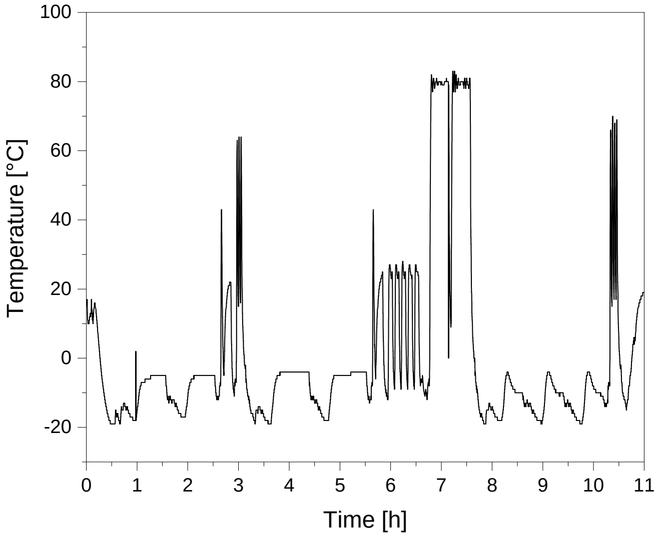

^{1}H NMR (600 MHz, $CDCl_3$) of **19**:

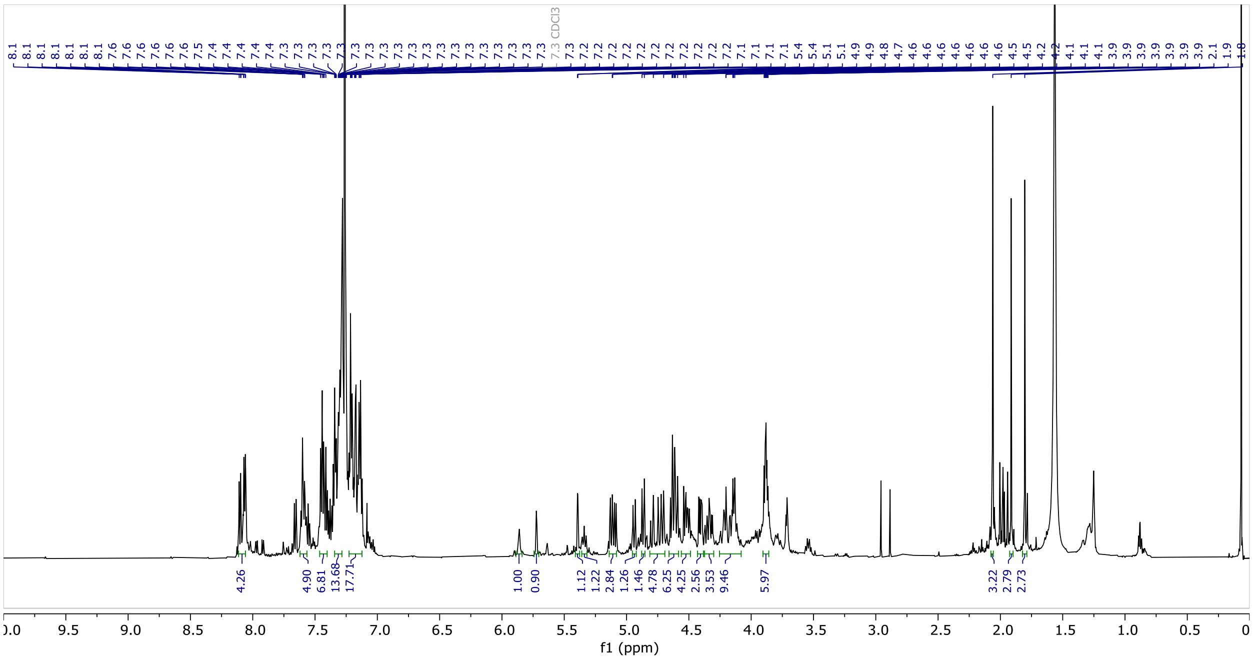

^{13}C NMR (176 MHz, $CDCl_3$) of **19**:

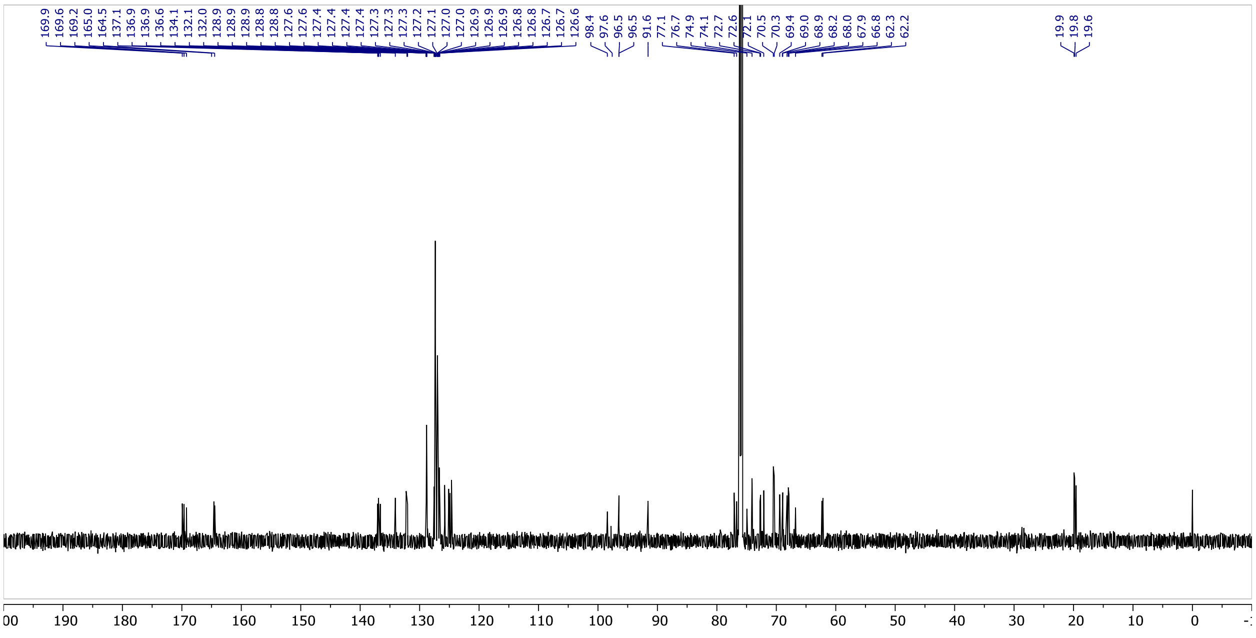

Coupled $^{13}C,^{1}H$ HSQC of **19**:

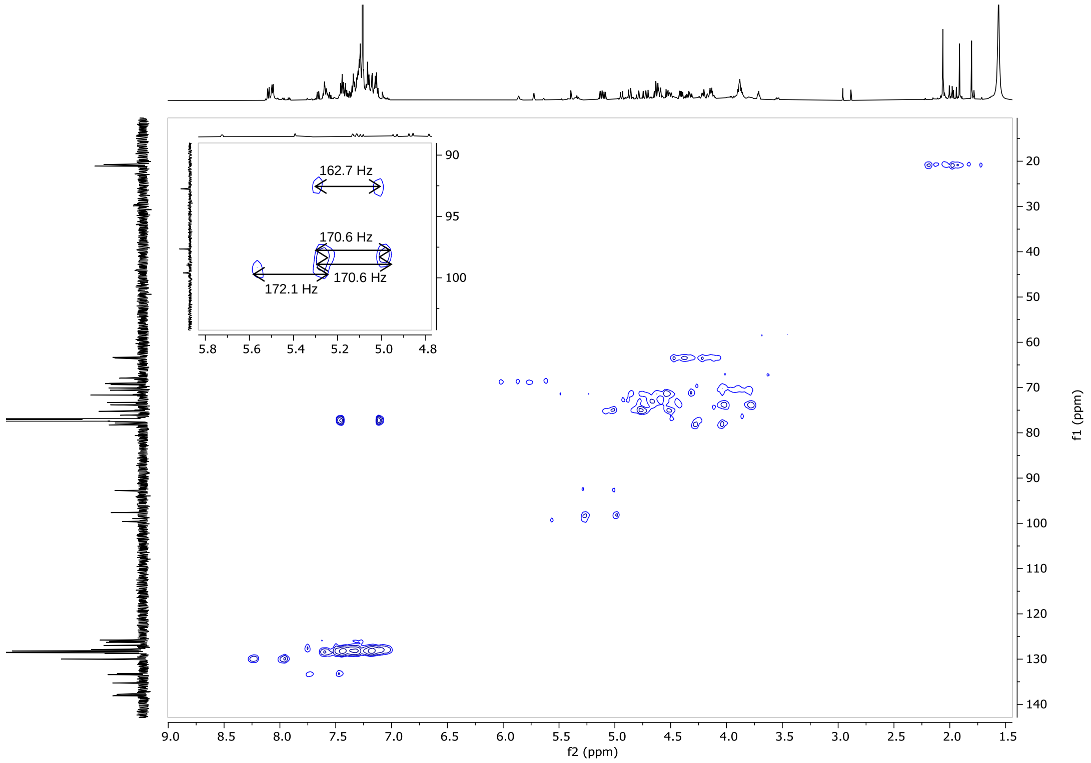

$^{13}C,^{1}H$ HSQC of **19**:

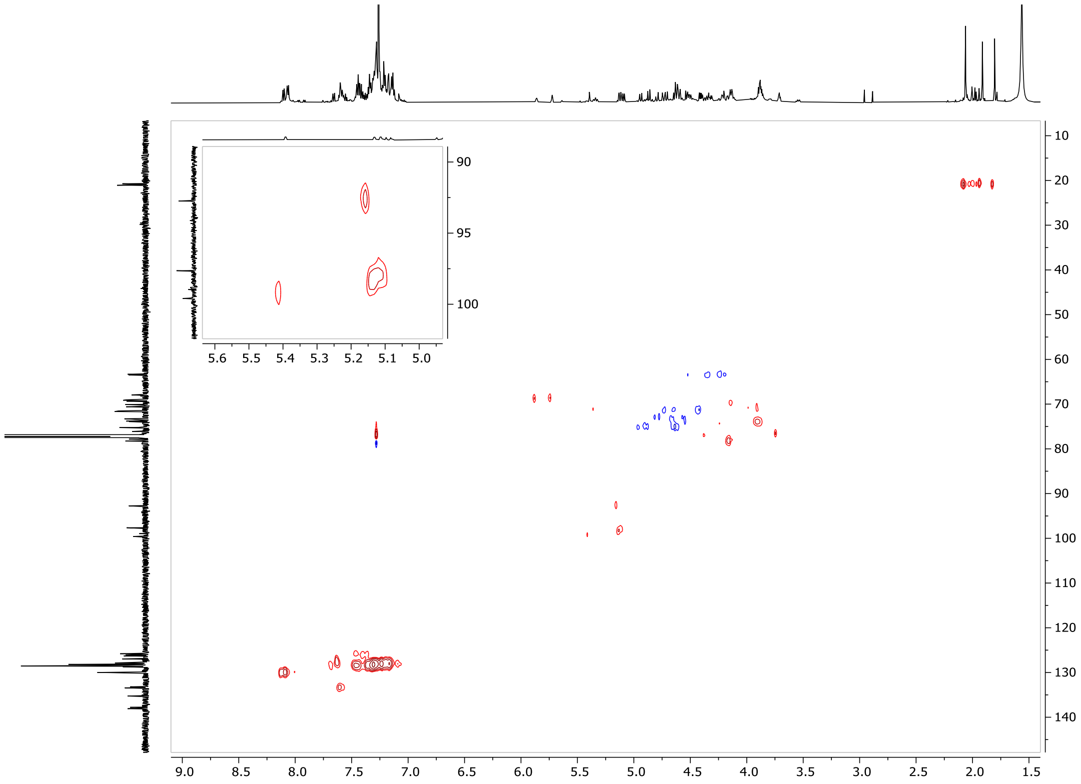

5.5.5 Hyperbranched Mannose Pentamer (20)

***N*-Benzyloxycarbonyl-5-amino-pentyl 2,3,4-tri-*O*-benzyl-α-L-fucopyranosyl-(1→2)-[6-*O*-acetyl-2-*O*-benzoyl-3,4-di-*O*-benzyl-α-D-mannopyranosyl-(1→3)]-[4-*O*-acetyl-3,6-di-*O*-benzyl-2-deoxy-2-*N*-trichloroacetyl-β-D-glucopyranosyl-(1→4)]-[6-*O*-acetyl-2-*O*-benzoyl-3,4-di-*O*-benzyl-β-D-galactopyranosyl-(1→6)]-α-D-mannopyranoside (20)**

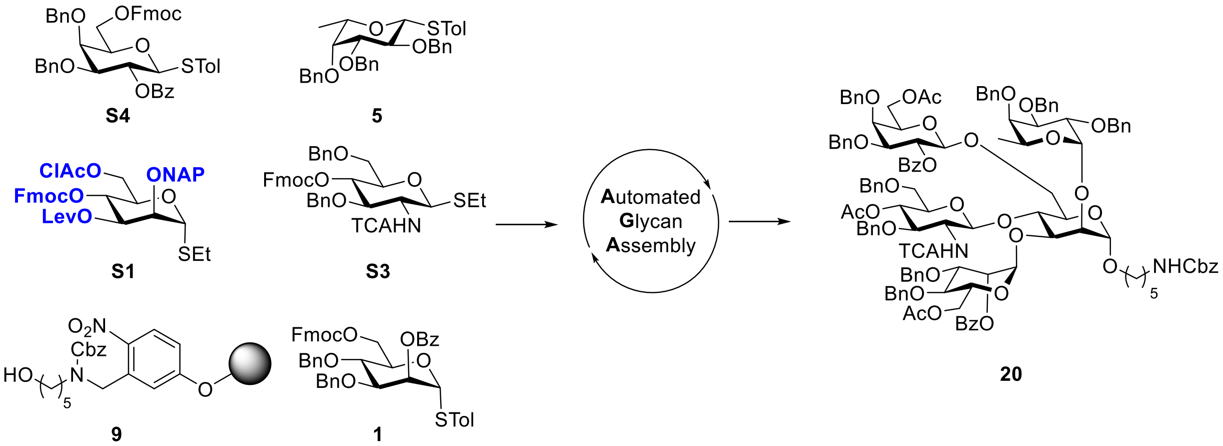

Repeat	Building Blocks	Modules	Notes
**1x**		**I** – Acidic Wash	
	**S1** (2 x 6.5 equiv.)	**IIa** – Glycosylation with thioglycoside – 2 cycles	-20 °C (T_1) 10 min (t_1)<br>0 °C (T_2) 35 min (t_2)
		**III** – Capping	
		**IVc** – Fmoc Deprotection	
**1x**		**I** – Acidic Wash	
	**S3** (6.5 equiv.)	**IIa** – Glycosylation with thioglycoside – 2 cycles	-20 °C (T_1) 10 min (t_1)<br>-10 °C (T_2) 45 min (t_2)
		**III** – Capping	
		**IVb** – Lev Deprotection	
**1x**		**I** – Acidic Wash	
	**1** (3 x 6.5 equiv.)	**IIa** – Glycosylation with thioglycoside	-20 °C (T_1) 10 min (t_1)<br>0 °C (T_2) 25 min (t_2)
		**IVe** – ClAc Deprotection	
**1x**		**I** – Acidic Wash	
	**S4** (3 x 6.5 equiv.)	**IIa** – Glycosylation with thioglycoside – 3 cycles	-30 °C (T_1) 10 min (t_1)<br>-10 °C (T_2) 25 min (t_2)
		**IVd** – NAP Deprotection	
**1x**		**I** – Acidic Wash	
	**5** (3 x 6.5 equiv.)	**IIa** – Glycosylation with thioglycoside – 3 cycles	-30 °C (T_1) 10 min (t_1)<br>-10 °C (T_2) 25 min (t_2)
		**IVa** – Fmoc Deprotection (6 cycles)	
		**III** – Capping (4 cycles)	

Photocleavage from solid support using **Method A-1** followed by purification by normal-phase HPLC (**Method B-3**, t_R = 22.8 min) afforded compound **20** (12 mg, 0.005 mmol, **32%**) as a colorless oil.

^{1}H NMR (600 MHz, [D$_6$]-DMSO): δ 8.02 (dd, *J* = 8.2, 1.6 Hz, 2H), 7.91 (dd, *J* = 8.2, 1.3 Hz, 2H), 7.66 – 7.62 (m, 1H), 7.61 – 7.55 (m, 1H), 7.48 (dt, *J* = 17.4, 7.8 Hz, 5H), 7.35 – 7.31 (m, 12H), 7.30 – 7.19 (m, 27H), 7.19 – 7.13 (m, 10H), 5.33 (dd, *J* = 10.2, 8.0 Hz, 1H), 5.22 (d, *J* = 2.2 Hz, 1H), 5.04 – 5.00 (m, 3H), 4.87 (d, *J* = 11.6 Hz, 1H), 4.80 – 4.41 (m, 22H), 4.37 – 4.19 (m, 5H), 4.17 – 4.09 (m, 3H), 4.05 – 3.81 (m, 12H), 3.78 – 3.71 (m, 2H), 3.63 – 3.54 (m, 3H), 2.94 – 2.88 (m, 2H), 1.98 (s, 3H), 1.96 (s, 3H), 1.83 (s, 3H), 1.30 – 1.24 (m, 8H), 1.03 (d, *J* = 6.2 Hz, 3H) ppm.

^{13}C NMR (151 MHz, [D$_6$]-DMSO, as per HSQC): δ 128.6, 128.5, 132.7, 132.3, 127.8, 127.1, 127.1, 126.7, 126.7, 71.2, 98.3, 95.2, 64.5, 73.3, 100.1, 73.6, 70.5, 100.2, 99.1, 69.5, 73.2, 71.6, 70.7, 70.4, 73.5, 72.0, 72.0, 62.3, 61.8, 61.7, 72.6, 69.3, 77.4, 65.9, 73.4, 79.0, 75.0, 77.9, 71.3, 55.9, 71.1, 77.2, 71.5, 39.7, 20.2, 19.8, 28.2, 27.8, 15.8 ppm.

HRMS (QToF): Calcd for $C_{128}H_{137}Cl_3N_2O_{32}Na$ [M + Na]$^+$ 2341.8112; found 2341.8408.

NP-HPLC of crude **20** (ELSD trace, **Method B-2**, t_R = 22.5 min):

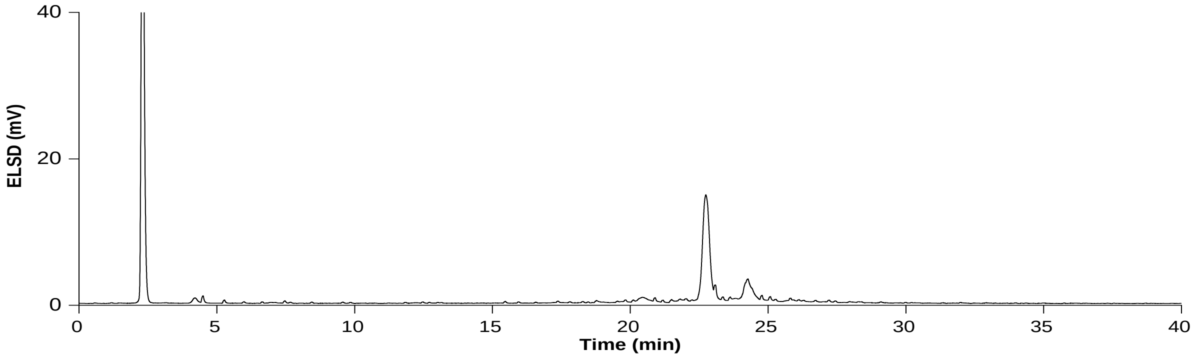

NP-HPLC of purified **20** (ELSD trace, **Method B-2**, t_R = 22.5 min):

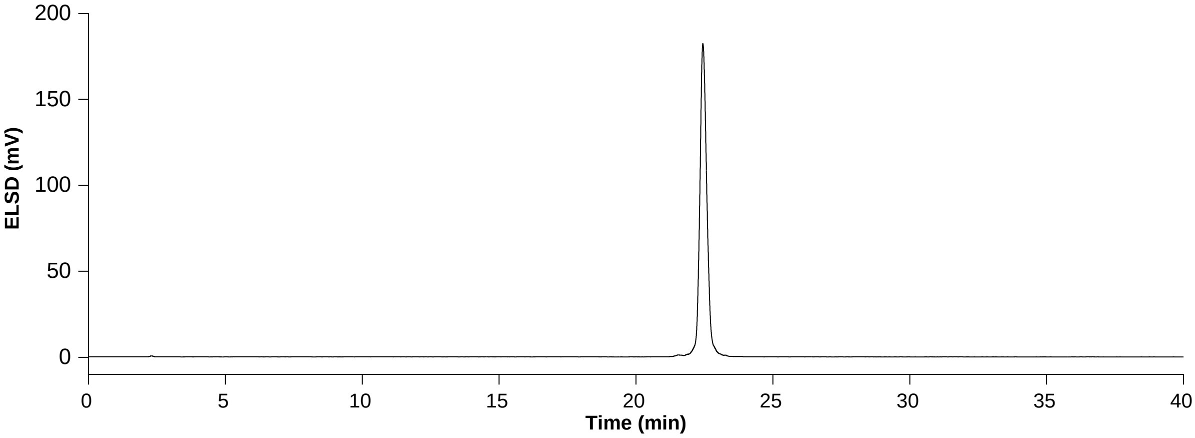

Temperature profile inside the reaction vessel during the synthesis of **20**:

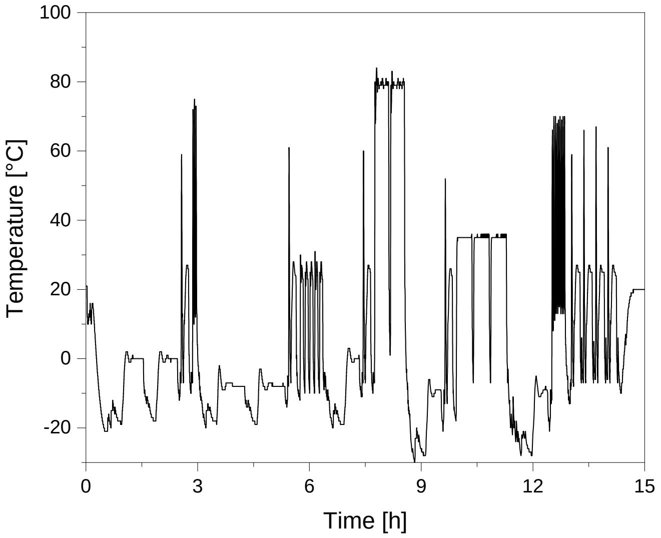

^{1}H NMR (600 MHz, [D_6]-DMSO, 80 °C) of **20**:

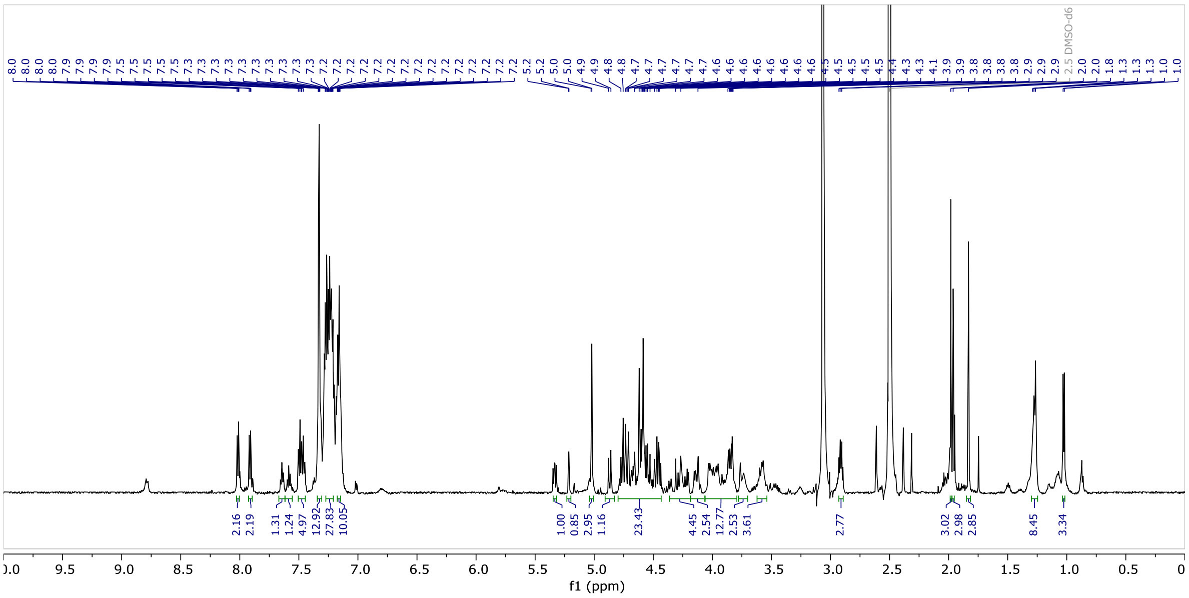

^{1}H NMR (600 MHz, [D_6]-DMSO, 23 to 80 °C) of **20**:

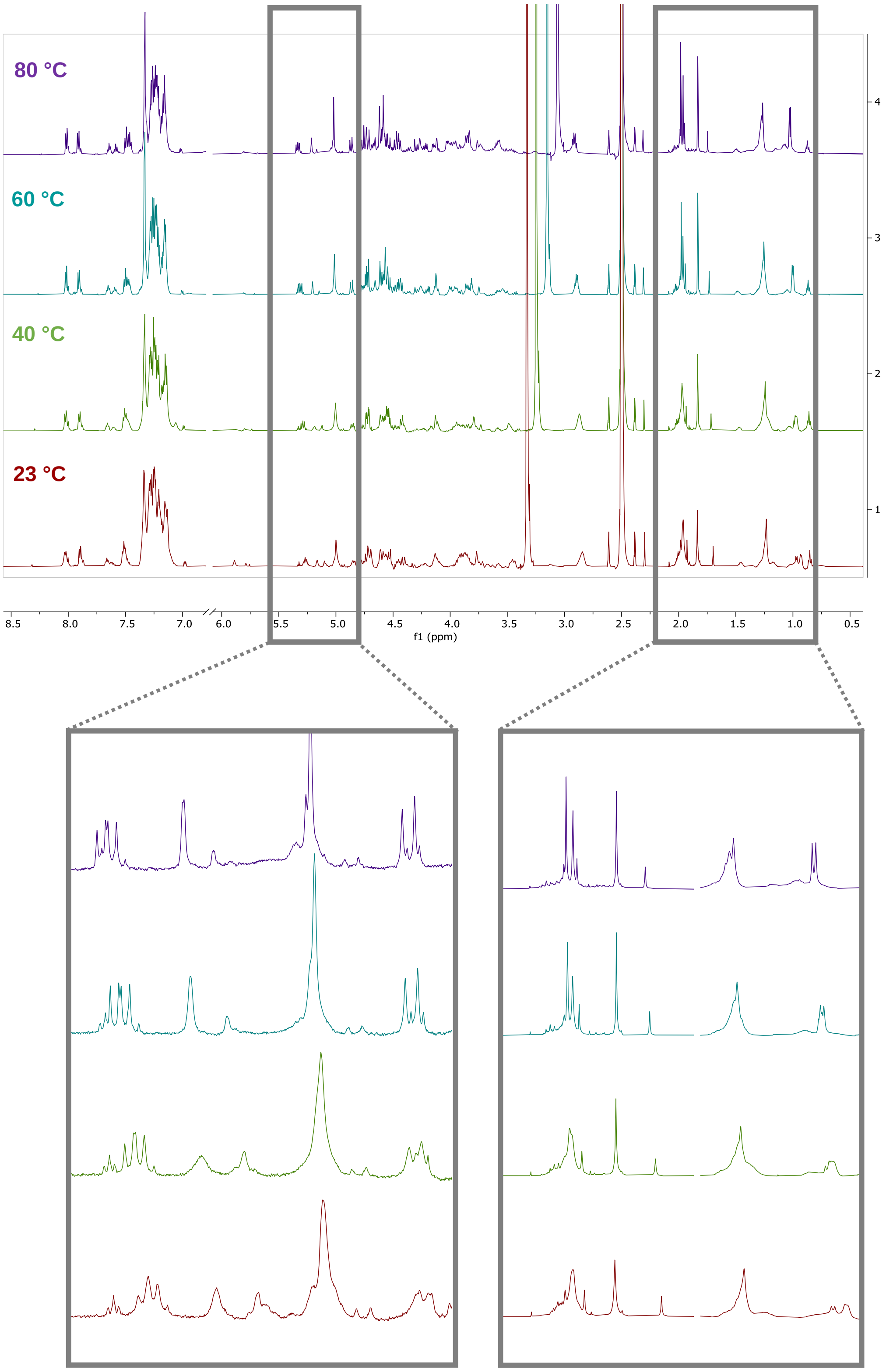

Coupled $^{13}C,^{1}H$ HSQC of **20** (80 °C):

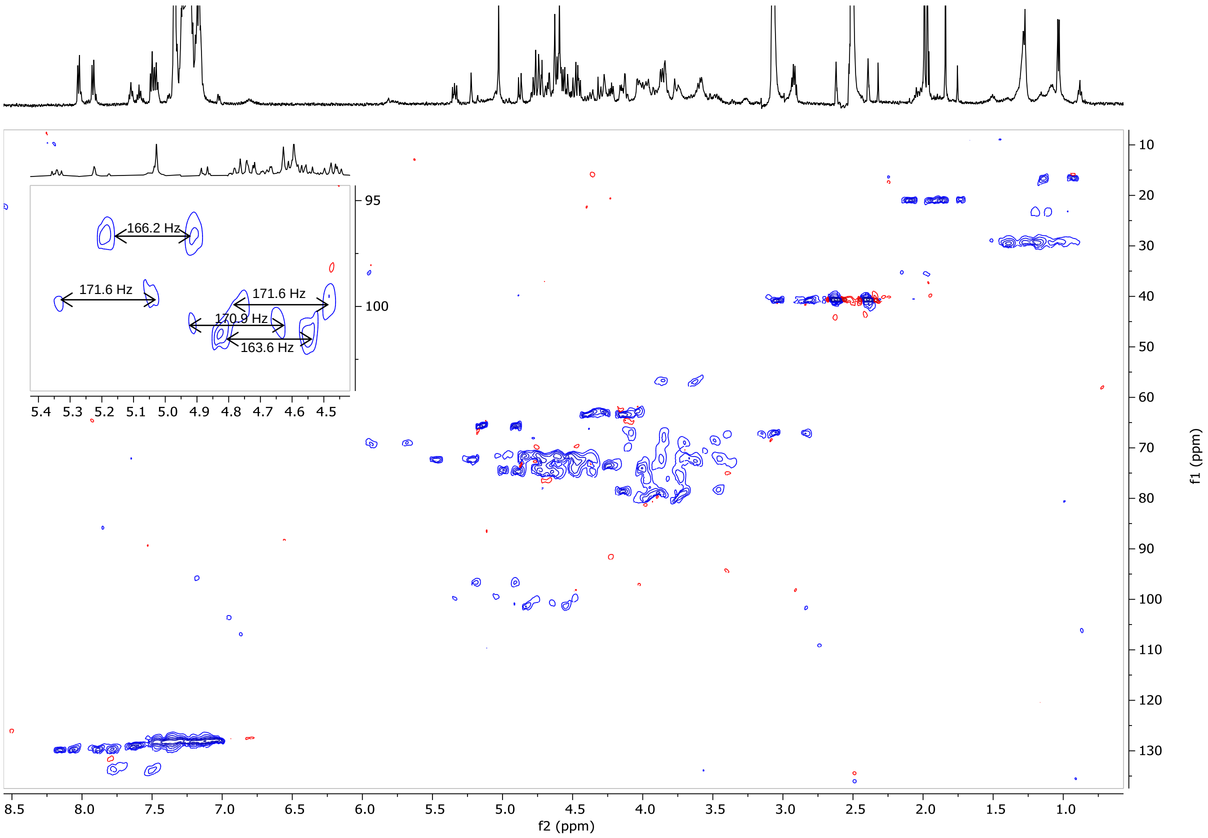

$^{13}C,^{1}H$ HSQC of **20** (80 °C):

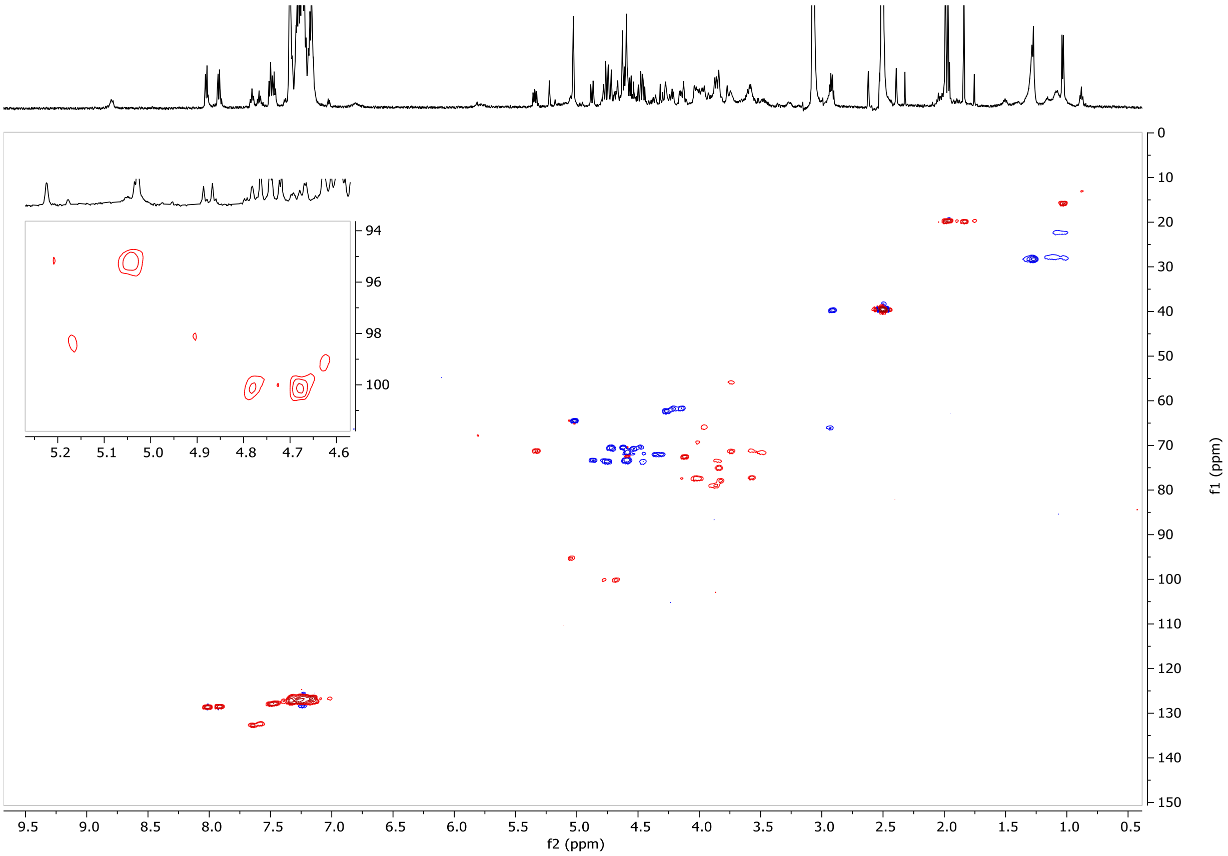

6 Preparation of Orthogonal Building Block 7

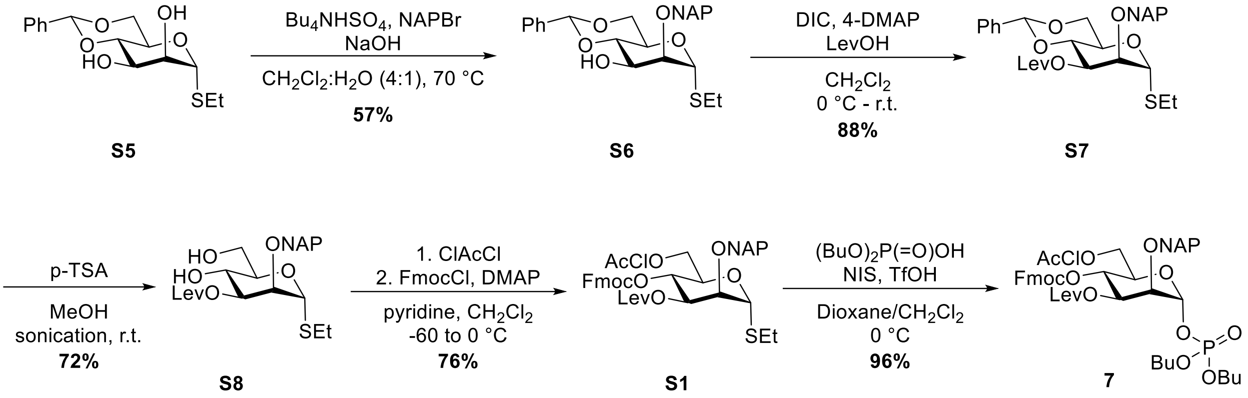

Figure S-3. Synthesis of orthogonal building block **7.**

Ethyl 4,6-*O*-benzylidene-2-*O*-(2-naphthalenylmethyl)-1-thio-α-D-mannopyranoside (S6)

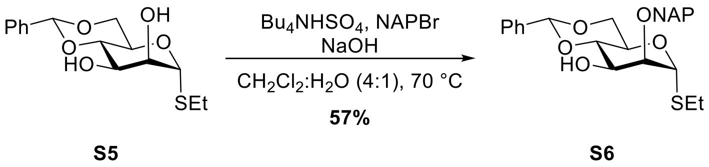

To a suspension of diol **S5** (20.0 g, 64.03 mmol, 1.0 equiv.) in CH_2Cl_2 (300 mL) was added Bu_4NHSO_4 (3.0 g, 12.17 mmol, 0.2 equiv.), followed by addition of 2-(bromomethyl)naphthalene (15.6 g, 70.43 mmol, 1.1 equiv.). An aqueous solution of NaOH (4.0 g in 100 mL) was added and the biphasic solution was refluxed at 70 °C overnight. The mixture was allowed to cool down to room temperature and the aqueous phase was extracted with CH_2Cl_2. The combined organic phase was washed with citric acid solution, dried over Na_2SO_4, filtered and concentrated. The title compound **S6** (16.8 g, 36.32 mmol, 57%) was obtained as a white solid after purification by column chromatography (SiO_2, Hex/EtOAc 3:1).

R_f = 0.44 (Hex/EtOAc 3:1).

^{1}H NMR (400 MHz, $CDCl_3$): δ 7.91 – 7.79 (m, 4H), 7.59 – 7.44 (m, 5H), 7.44 – 7.31 (m, 3H), 5.59 (s, 1H), 5.42 (d, *J* = 1.2 Hz, 1H), 4.98 – 4.78 (m, 2H), 4.31 – 4.16 (m, 2H), 4.13 – 4.05 (m, 1H), 4.04 – 3.94 (m, 2H), 3.94 – 3.82 (m, 1H), 2.70 – 2.51 (m, 2H), 2.48 (d, *J* = 7.9 Hz, 1H), 1.23 (t, *J* = 7.4 Hz, 3H) ppm.

^{13}C NMR (101 MHz, $CDCl_3$): δ 137.4, 134.9, 133.3, 133.2, 129.3, 128.7, 128.4, 128.1, 127.9, 127.1, 126.4, 126.4, 126.3, 125.9, 102.3, 82.6, 80.2, 79.9, 73.4, 69.2, 68.7, 64.0, 25.4, 15.0 ppm.

$[\alpha]_D$ 31.11 cm^{-1} (*c* 1, $CHCl_3$).

IR (film): 3470, 3385, 3318, 2931, 2906, 2876, 1702, 1600, 1510, 1455, 1422, 1383, 1271, 1244, 1209, 1168, 1088, 1057, 1048, 1035, 972, 749 cm^{-1}. HRMS (QToF): Calcd for $C_{26}H_{28}O_5SNa$ $[M + Na]^+$ 475.1550; found 475.1552.

^{1}H NMR (400 MHz, $CDCl_3$) of **S6**:

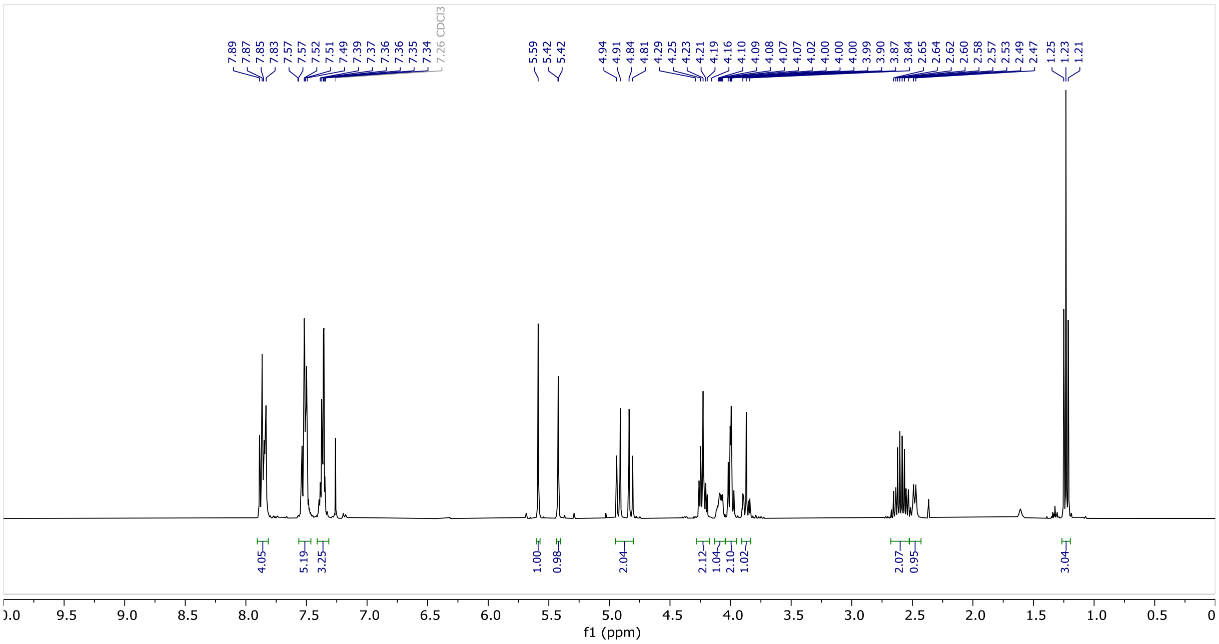

^{13}C NMR (101 MHz, $CDCl_3$) of **S6**:

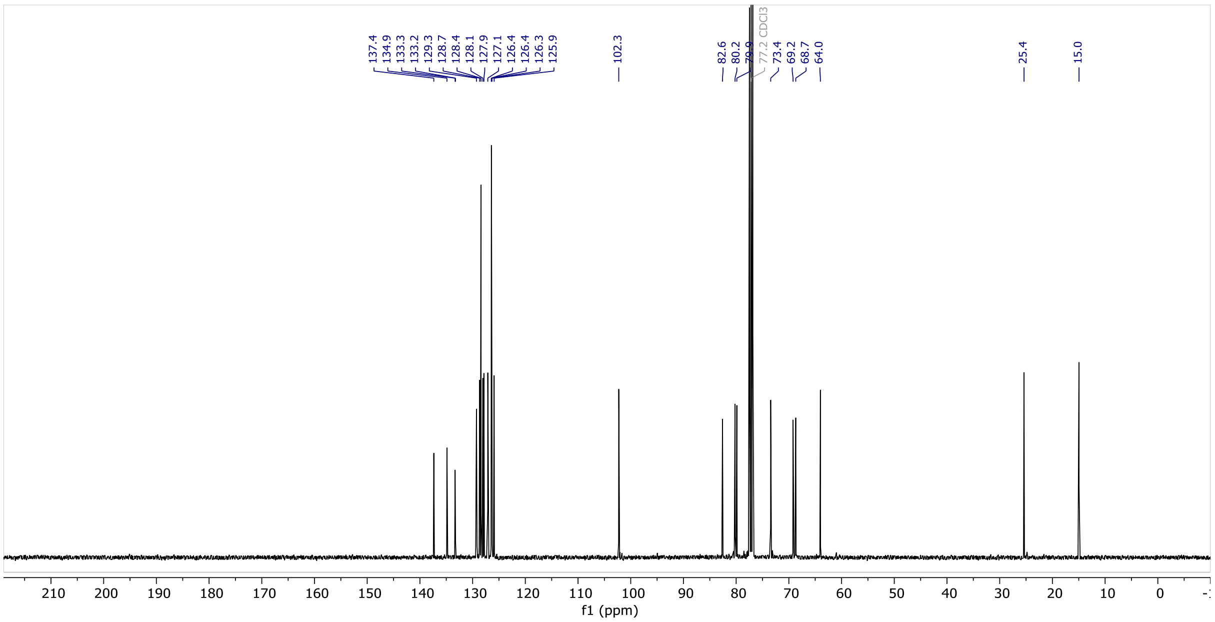

Ethyl 4,6-*O*-benzylidene-2-*O*-levulinyl-2-*O*-(2-naphthalenylmethyl)-1-thio-α-D-manno-pyranoside (S7)

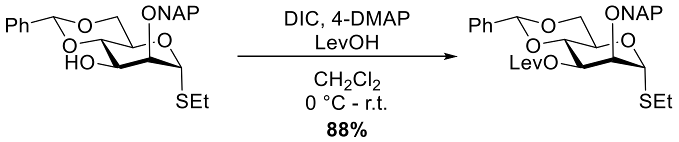

S6 S7

To a solution of **S6** (15.8 g, 34.91 mmol, 1.0 equiv.) and levulinic acid (7.1 mL, 69.82 mmol, 2.0 equiv.) in anhydrous CH_2Cl_2 (50 mL) was added dropwise a premixed solution of DIC (16.1 mL, 104.7 mmol, 3.0 equiv.) and DMAP (0.9 g, 6.98 mmol, 0.2 equiv.) in anhydrous CH_2Cl_2 (20 mL) at 0 °C. The reaction was stirred at room temperature overnight. The reaction mixture was filtered through Celite, the filtrate was washed with aqueous $NaHCO_3$ and the aqueous phase was extracted with CH_2Cl_2. The combined organic phase was dried over Na_2SO_4, filtered and concentrated. The title compound **S7** (17.0 g, 30.87 mmol, 88%) was obtained as a yellow syrup after purification by column chromatography (SiO_2, Hex/EtOAc 3:1).

R_f = 0.25 (Hex/EtOAc 3:1).

^{1}H NMR (400 MHz, $CDCl_3$): δ 7.88 – 7.81 (m, 4H), 7.55 (dd, *J* = 8.5, 1.8 Hz, 1H), 7.52 – 7.44 (m, 4H), 7.39 – 7.32 (m, 3H), 5.58 (s, 1H), 5.33 (d, *J* = 1.5 Hz, 1H), 5.23 (dd, *J* = 9.9, 3.5 Hz, 1H), 4.83 (q, *J* = 12.0 Hz, 2H), 4.34 – 4.21 (m, 3H), 4.14 (dd, *J* = 3.5, 1.4 Hz, 1H), 3.90 (t, *J* = 10.0 Hz, 1H), 2.66 – 2.48 (m, 6H), 2.08 (s, 3H), 1.21 (t, *J* = 7.5 Hz, 3H) ppm.

^{13}C NMR (101 MHz, $CDCl_3$): δ 206.5, 172.2, 137.4, 135.2, 133.3, 133.2, 129.2, 128.5, 128.4, 128.1, 127.8, 127.3, 126.4, 126.4, 126.3, 126.2, 101.3, 83.3, 79.2, 76.5, 73.7, 71.2, 68.8, 64.6, 37.8, 29.9, 28.0, 25.5, 14.9 ppm.

$[\alpha]_D$ 32.53 cm^{-1} (*c* 1, $CHCl_3$).

IR (film): 2930, 1741, 1719, 1365, 1206, 1180, 1157, 1097, 1014, 753, 700 cm^{-1}.

HRMS (QToF): Calcd for $C_{31}H_{34}O_7SNa$ $[M + Na]^+$ 573.1917; found 573.1921.

^{1}H NMR (400 MHz, $CDCl_3$) of **S7**:

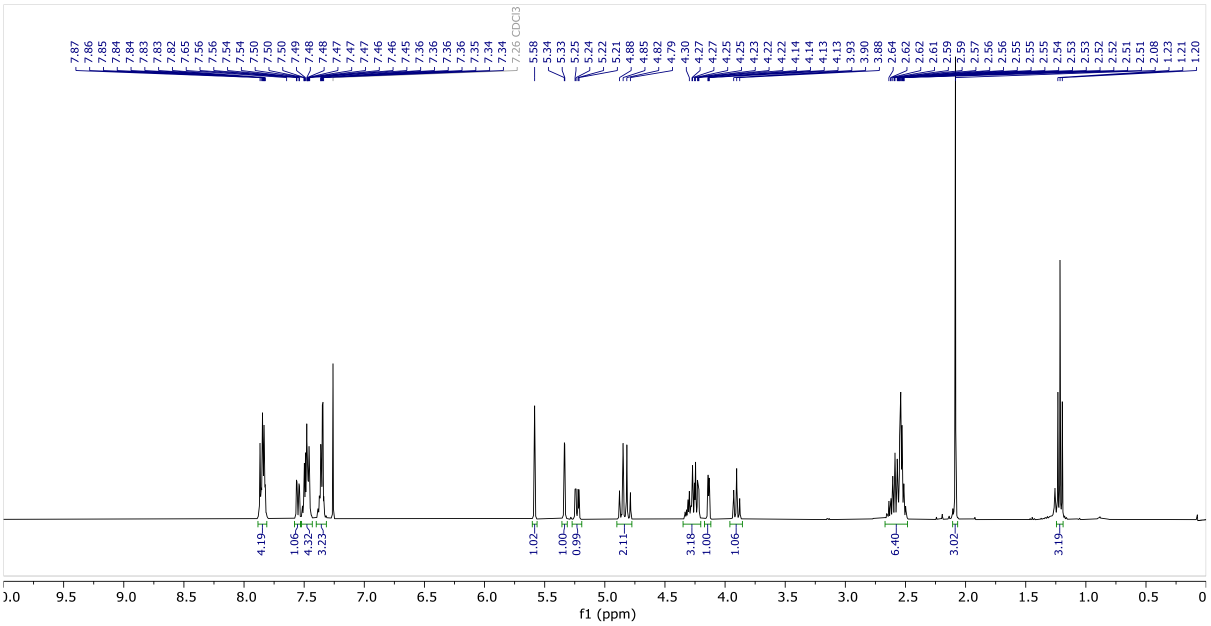

^{13}C NMR (101 MHz, $CDCl_3$) of **S7**:

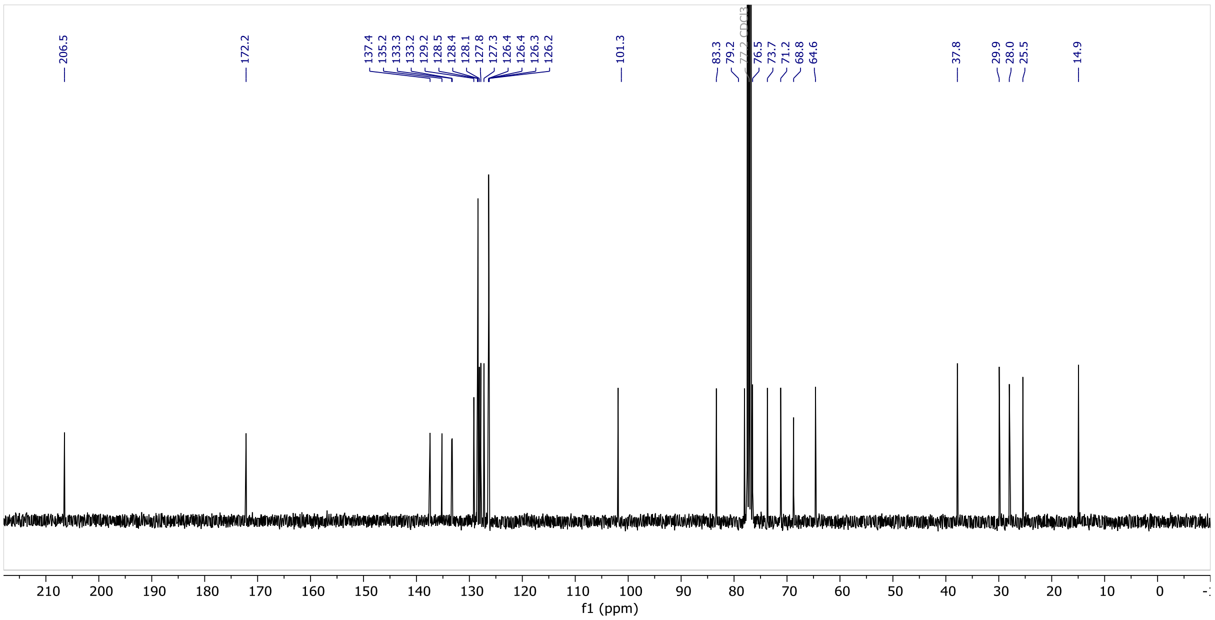

Ethyl 2-*O*-levulinyl-2-*O*-(2-naphthalenylmethyl)-1-thio-α-D-mannopyranoside (S8)

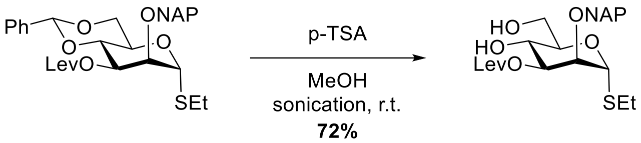

To a suspension of **S7** (16.5 g, 36.46 mmol, 1.0 equiv.) in MeOH (100 mL) was added *p*-TSA (3.1 g, 18.23 mmol, 0.5 equiv.). The reaction mixture was sonicated for one hour at room temperature before pyridine (5 mL) was added. The solvent was removed and the residue was redissolved in ethyl acetate, washed with water, dried over Na_2SO_4, filtered and concentrated. The title compound **S8** (12.2 g, 26.37 mmol, 72%) was obtained as a colorless oil after purification by column chromatography (SiO_2, Hex/EtOAc = 1:1 to 2:3).

$\mathbf{R_f}$ = 0.25 (Hex/EtOAc 2:3).

^{1}H NMR (400 MHz, $CDCl_3$): δ 7.87 – 7.77 (m, 4H), 7.54 – 7.44 (m, 3H), 5.35 (d, *J* = 1.5 Hz, 1H), 5.08 (dd, *J* = 9.8, 3.3 Hz, 1H), 4.88 – 4.70 (m, 2H), 4.17 (t, *J* = 9.7 Hz, 1H), 4.11 – 4.02 (m, 1H), 3.98 (dd, *J* = 3.4, 1.5 Hz, 1H), 3.90 (s, 2H), 2.74 – 2.37 (m, 6H), 2.12 (s, 3H), 1.23 (t, *J* = 7.4 Hz, 3H) ppm.

^{13}C NMR (101 MHz, $CDCl_3$): δ 207.4, 172.8, 135.2, 133.2, 133.1, 128.3, 127.9, 127.7, 126.8, 126.3, 126.1, 125.9, 82.1, 77.5, 74.7, 73.0, 72.4, 66.9, 62.7, 38.1, 29.8, 28.1, 25.2, 14.7 ppm.

$[\alpha]_D$ 5.33 cm^{-1} (*c* 1, $CHCl_3$).

IR (film): 3456, 2928, 1720, 1365, 1159, 1083, 774 cm^{-1}.

HRMS (QToF): Calcd for $C_{24}H_{30}O_7SNa$ $[M + Na]^+$ 485.1604; found 485.1605.

^{1}H NMR (400 MHz, $CDCl_3$) of **S8**:

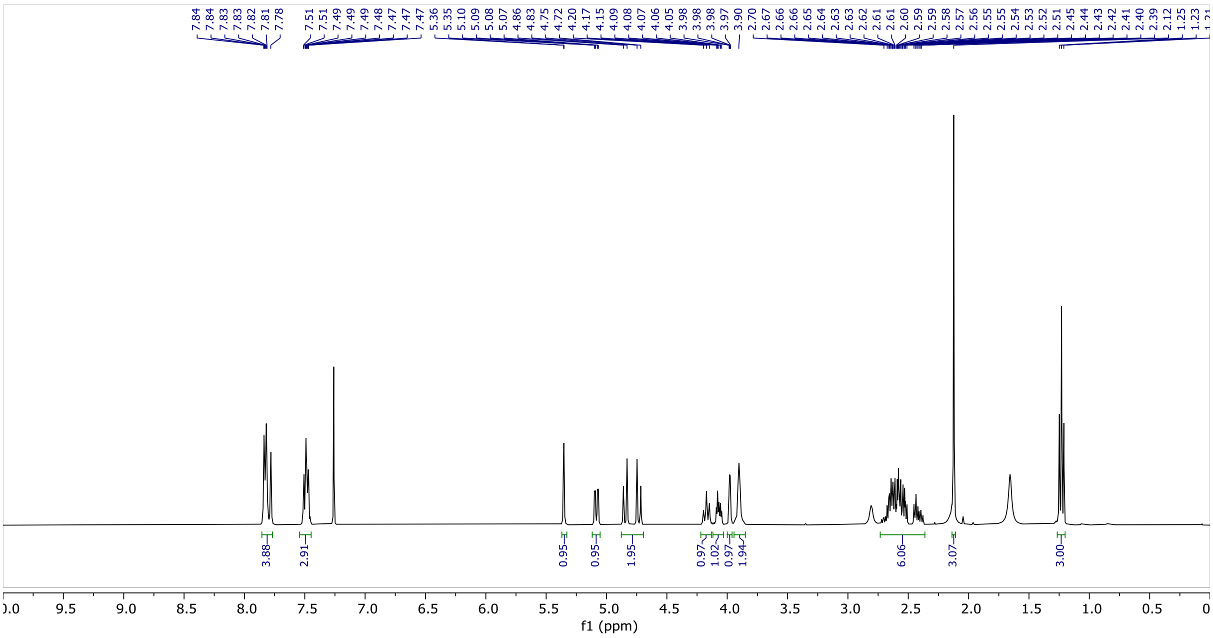

^{13}C NMR (101 MHz, $CDCl_3$) of **S8**:

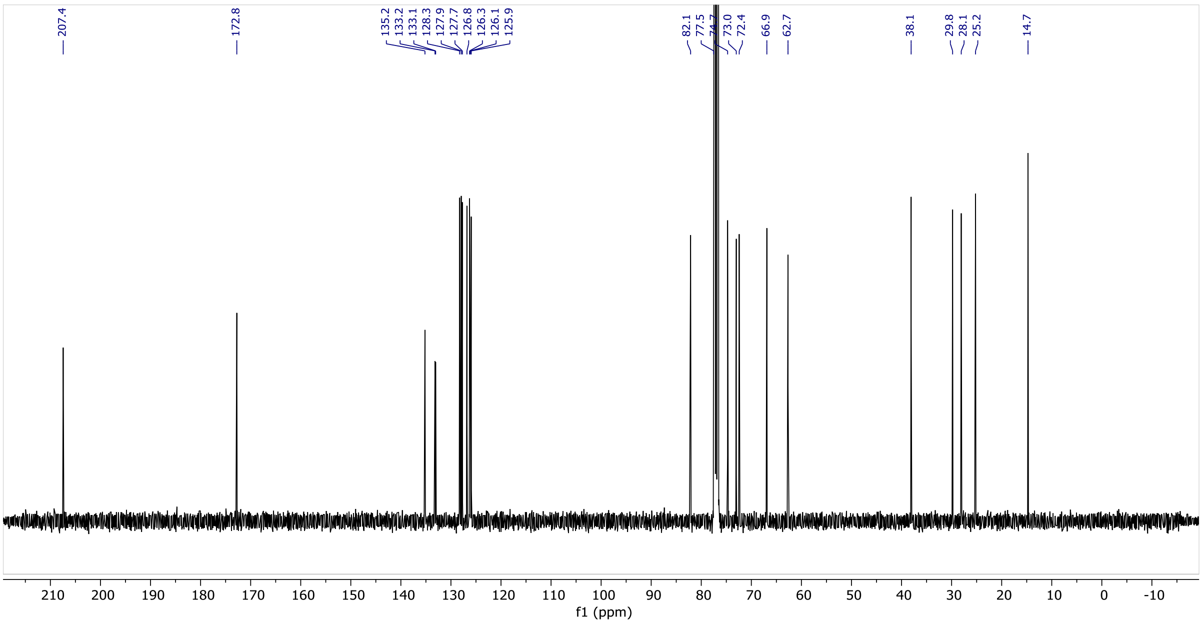

Ethyl 6-*O*-(2-chloroacetyl)-4-*O*-fluorenylmethoxycarbonyl-2-*O*-levulinyl-2-*O*-(2-naphthalenylmethyl)-1-thio-α-D-mannopyranoside (S1)

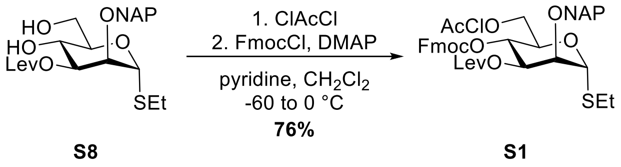

To a solution of **S8** (2.0 g, 4.32 mmol, 1.0 equiv.) and anhydrous pyridine (1.75 mL, 21.62 mmol, 5.0 equiv.) in anhydrous CH_2Cl_2 (20 mL) at -60 °C was added chloroacetyl chloride (0.38 mL, 4.76 mmol, 1.1 equiv.). The reaction was stirred for 10 min, aqueous citric acid solution (2 mL) was added and the mixture was allowed to warm up to room temperature. Water was added and the aqueous phase was extracted with CH_2Cl_2. The combined organic phase was dried over Na_2SO_4, filtered and concentrated. The residue (R_f = 0.53 (Hex/AcOEt 2:3)) was dissolved in anhydrous CH_2Cl_2 (30 mL) and anhydrous pyridine (1.75 mL, 21.62 mmol, 5.0 equiv.) was added followed by a solution of FmocCl (1.7 g, 6.47 mmol, 1.5 equiv.) in anhydrous CH_2Cl_2 (20 mL) at 0 °C. DMAP (53 mg, 0.43 mmol, 0.1 equiv.) was added and the reaction mixture was stirred for one hour at 0 °C. Aqueous citric acid solution (10 mL) was added and the mixture was allowed to warm up to room temperature. The aqueous phase was extracted with CH_2Cl_2 and the combined organic phase was dried over Na_2SO_4, filtered and concentrated. The title compound **S1** (2.5 g, 3.28 mmol, 76%) was obtained as a white solid after purification by column chromatography (SiO_2, Hex/EtOAc = 9:1 to 3:1).

R_f = 0.26 (Hex/EtOAc 3:1).

^{1}H NMR (400 MHz, $CDCl_3$): δ 7.87 – 7.72 (m, 6H), 7.64 – 7.57 (m, 2H), 7.54 – 7.45 (m, 3H), 7.44 – 7.38 (m, 2H), 7.35 – 7.28 (m, 2H), 5.38 – 5.30 (m, 2H), 5.22 (dd, *J* = 10.1, 3.2 Hz, 1H), 4.89 – 4.74 (m, 2H), 4.50 – 4.24 (m, 6H), 4.10 (d, *J* = 2.1 Hz, 2H), 4.06 (dd, *J* = 3.2, 1.6 Hz, 1H), 2.69 – 2.52 (m, 2H), 2.49 – 2.34 (m, 4H), 2.00 (s, 3H), 1.24 (t, *J* = 7.4 Hz, 3H) ppm.

^{13}C NMR (101 MHz, $CDCl_3$): δ 206.2, 171.9, 167.3, 154.5, 143.4, 143.3, 141.4, 141.4, 135.1, 133.3, 133.2, 129.2, 128.4, 128.4, 128.1, 128.0, 127.8, 127.4, 127.3, 127.0, 126.4, 126.2, 126.1, 125.4, 125.4, 125.2, 120.2, 120.2, 82.4, 73.2, 71.8, 70.8, 70.5, 68.6, 64.2, 46.7, 40.9, 37.6, 29.8, 29.8, 27.9, 25.5, 14.9 ppm.

$[\alpha]_D$ 8.93 cm^{-1} (*c* 1, $CHCl_3$).

IR (film): 2958, 1754, 1720, 1451, 1364, 1261, 1156, 1101, 989, 760, 744 cm^{-1}.

HRMS (QToF): Calcd for $C_{41}H_{41}SO_{10}ClNa$ $[M + Na]^+$ 783.2001; found 783.2043.

^1H NMR (400 MHz, $CDCl_3$) of **S1**:

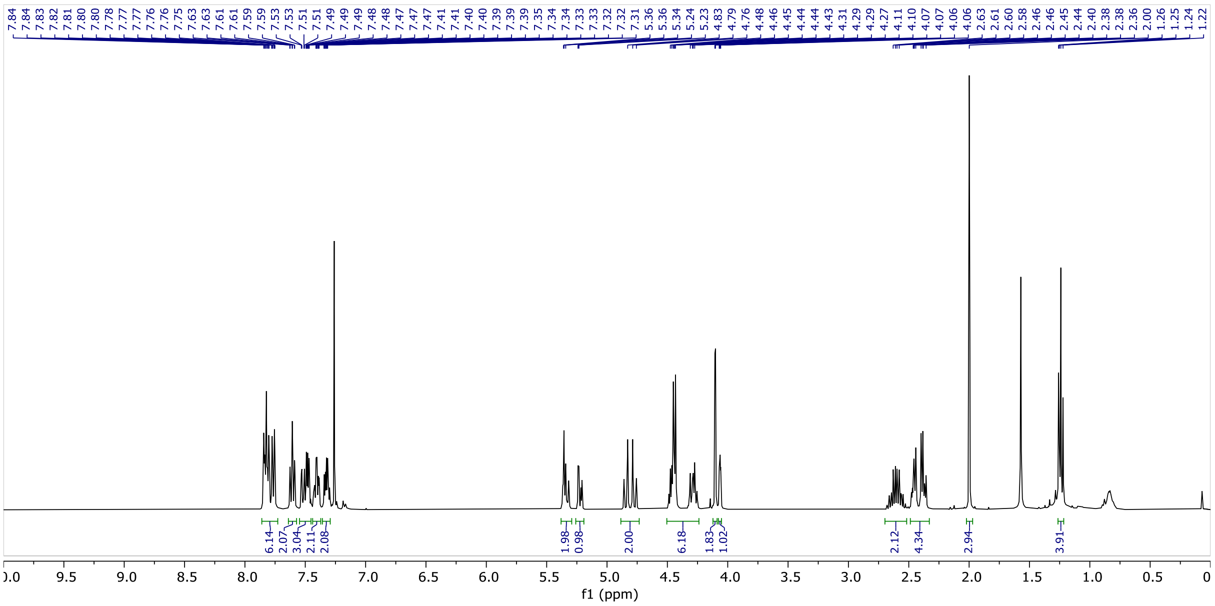

^{13}C NMR (101 MHz, $CDCl_3$) of **S1**:

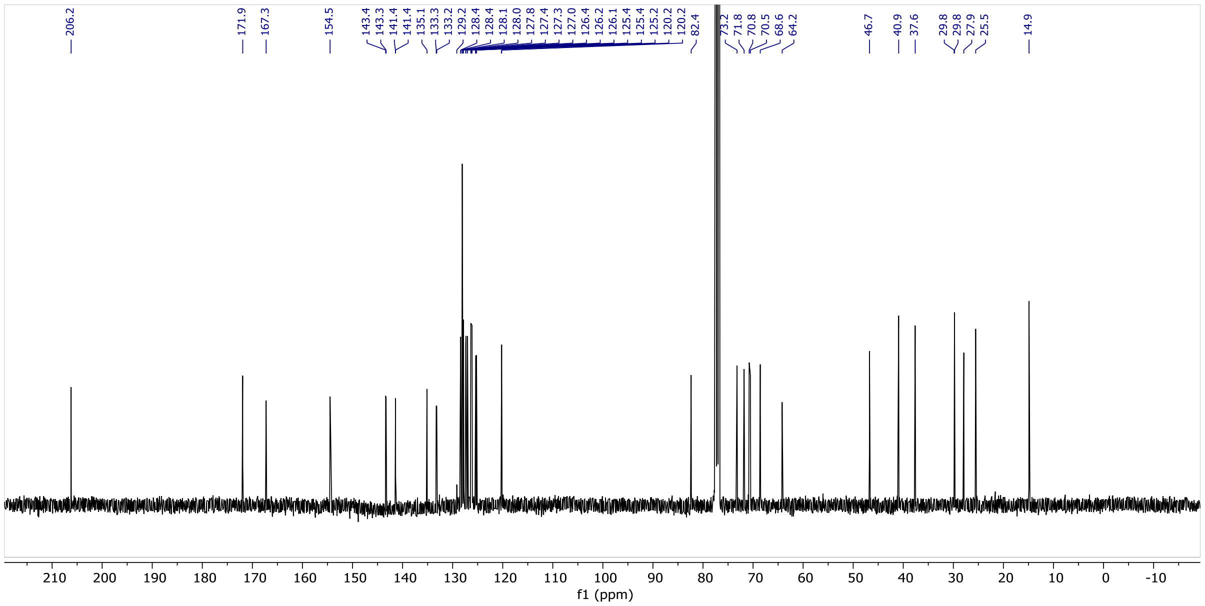

$^{1}H,^{1}H$ COSY of **S1**:

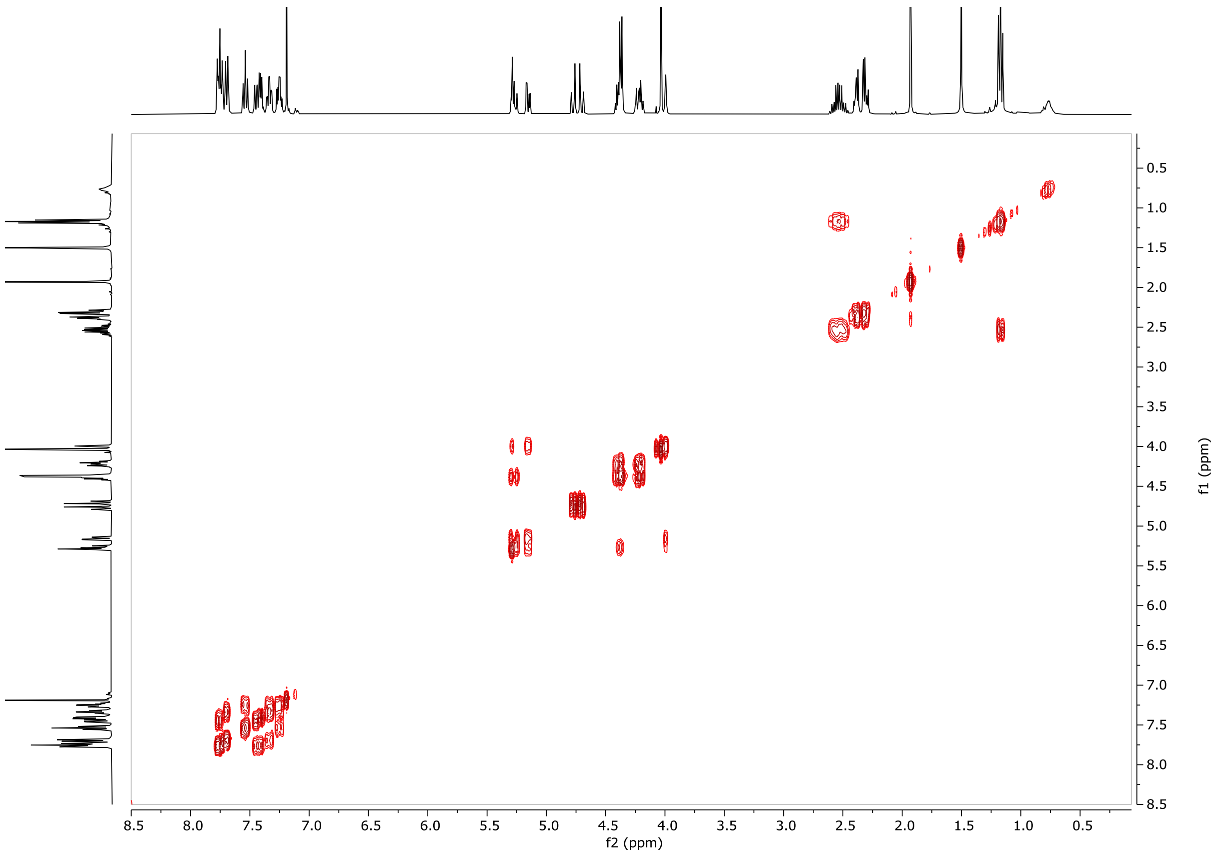

$^{13}C,^{1}H$ HSQC of **S1**:

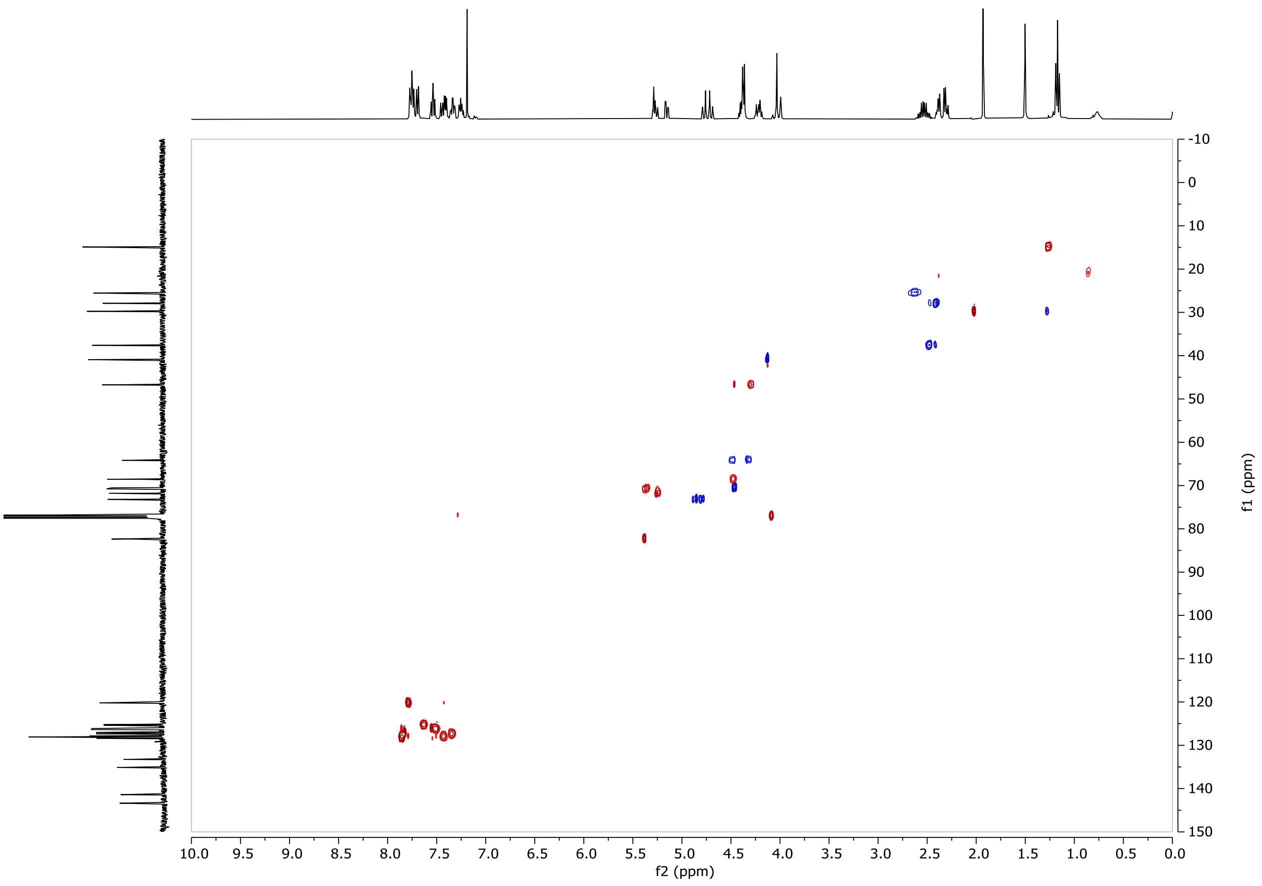

Dibutoxyphosphoryloxy 6-*O*-(2-chloroacetyl)-4-*O*-fluorenylmethoxycarbonyl-2-*O*-levulinyl-2-*O*-(2-naphthalenylmethyl)-α-D-mannopyranoside (7)

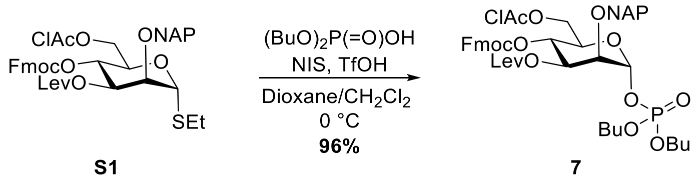

To a solution of thioglycoside **S1** (1.0 g, 1.31 mmol, 1.0 equiv.) and dibutyl hydrogen phosphate (0.52 mL, 2.63 mmol, 2.0 equiv.) in anhydrous CH_2Cl_2 (6 mL) a solution of NIS (530 mg, 2.36 mmol, 1.8 equiv.) and TfOH (35 µL, 0.39 mmol, 0.3 equiv.) in anhydrous CH_2Cl_2/dioxane (1:1, 4 mL) was added dropwise at 0 °C. The reaction was stirred at the same temperature for 2 h. The reaction mixture was diluted with CH_2Cl_2 (10 mL) and 10% sodium thiosulfate solution was added. The bilayer mixture was extracted with CH_2Cl_2, dried over Na_2SO_4, filtered and concentrated. The title compound **7** (1.2 g, 1.26 mmol, 96%) was obtained as a white solid after purification by column chromatography (SiO_2, Hex/EtOAc = 2:1).

R_f = 0.23 (Hex/EtOAc 3:1).

^{1}H NMR (400 MHz, $CDCl_3$): δ 7.86 – 7.74 (m, 6H), 7.60 (t, *J* = 7.3 Hz, 2H), 7.54 – 7.46 (m, 3H), 7.45 – 7.36 (m, 2H), 7.33 (dd, *J* = 8.7, 3.7 Hz, 2H), 5.68 (dd, *J* = 6.4, 2.0 Hz, 1H), 5.43 – 5.28 (m, 2H), 4.86 – 4.82 (m, 2H), 4.48 – 4.25 (m, 5H), 4.26 – 4.20 (m, 1H), 4.10 (d, *J* = 3.1 Hz, 2H), 4.07 – 3.95 (m, 5H), 2.50 – 2.36 (m, 4H), 2.01 (s, 3H), 1.65 – 1.54 (m, 4H), 1.42 – 1.30 (m, 4H), 0.92 (q, *J* = 7.5 Hz, 6H) ppm.

^{13}C NMR (101 MHz, $CDCl_3$): δ 205.2, 172.3, 167.3, 155.1, 143.4, 142.2, 134.8, 133.8, 128.5, 128.1, 127.8, 127.4, 127.3, 127.2, 126.4, 126.3, 126.1, 125.4, 125.2, 119.8, 95.9, 75.1, 73.8, 70.6, 70.5, 70.0, 69.9, 68.2, 63.6, 46.7, 40.9, 37.6, 32.4, 29.7, 27.9, 18.7, 13.7 ppm.

$[\alpha]_D$ -6.32 cm^{-1} (*c* 1, $CHCl_3$).

IR (film): 2964, 1754, 1720, 1451, 1365, 1261, 1153, 1029, 957, 744 cm^{-1}.

HRMS (QToF): Calcd for $C_{47}H_{54}ClPO_{14}Na$ $[M + Na]^+$ 931.2832; found 931.2884.

^{1}H NMR (400 MHz, $CDCl_3$) of **7**:

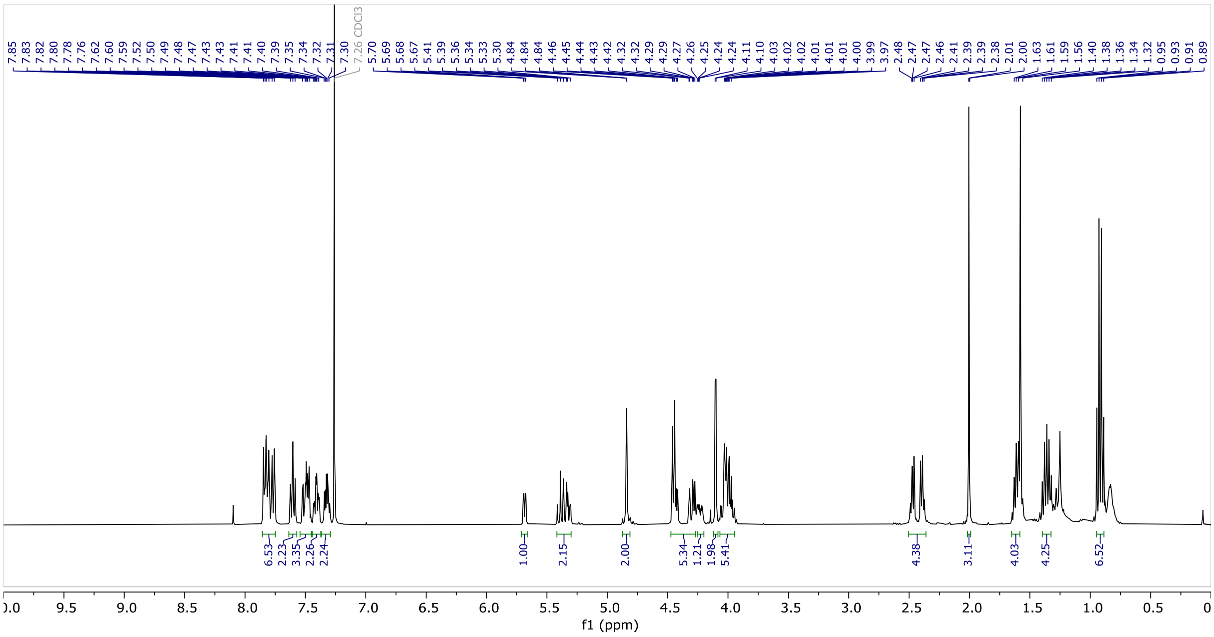

^{13}C NMR (101 MHz, $CDCl_3$) of **7**:

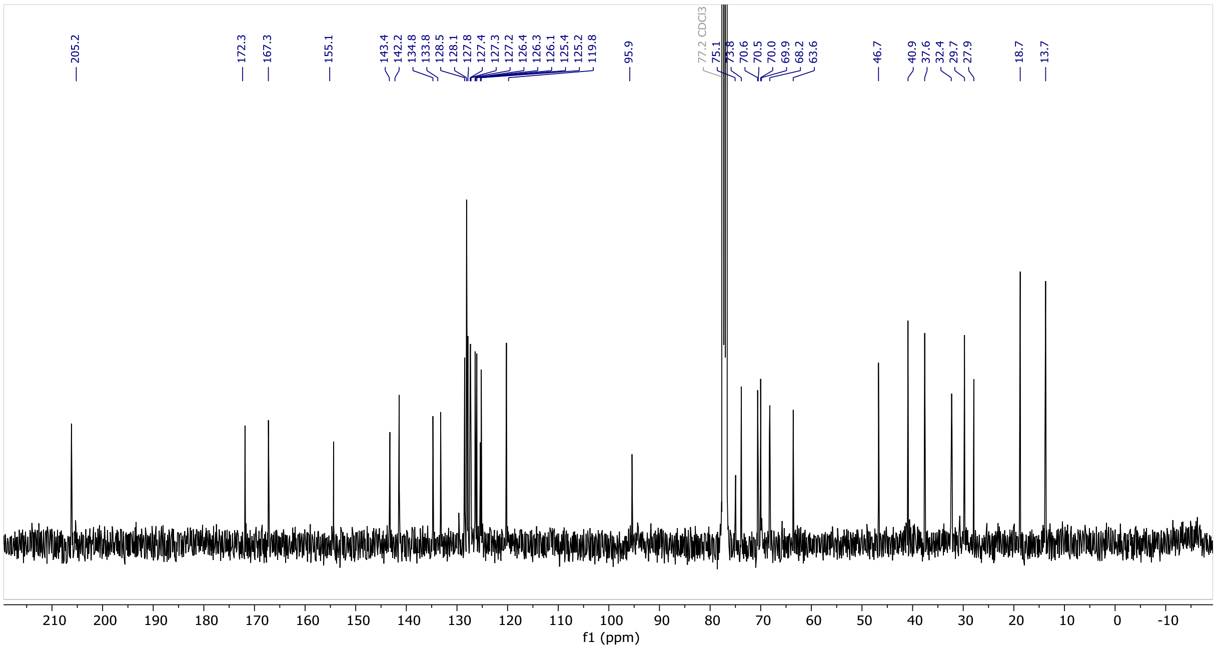

^{31}P NMR (162 MHz, $CDCl_3$) of **7**:

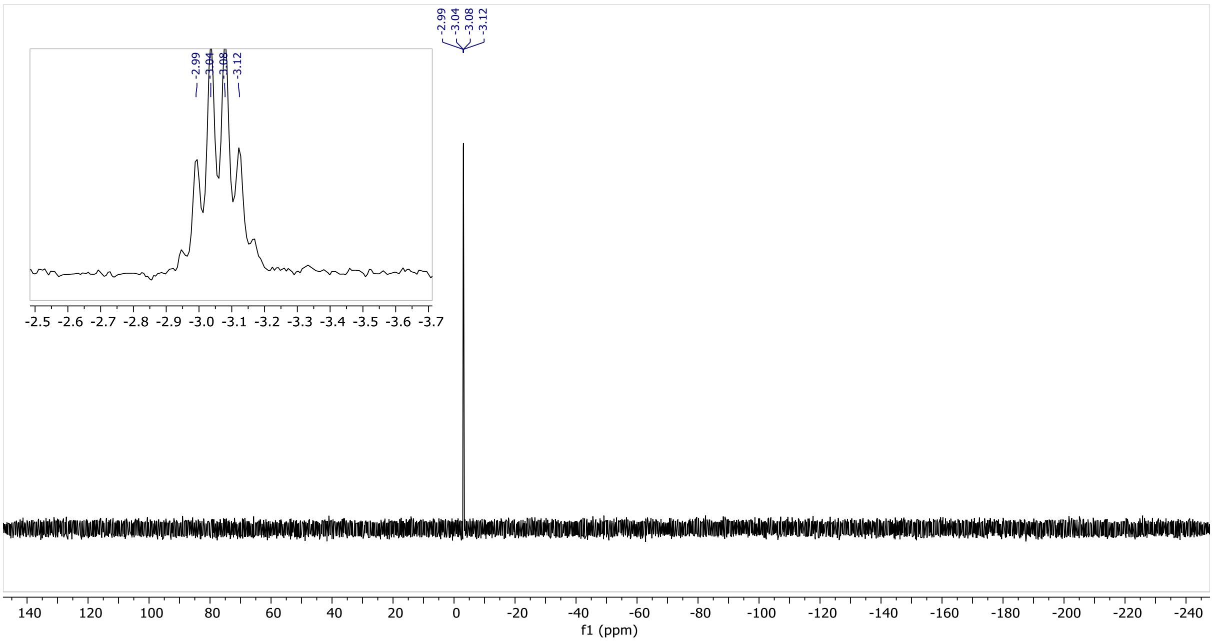

$^{13}C,^{1}H$ HSQC of **7**:

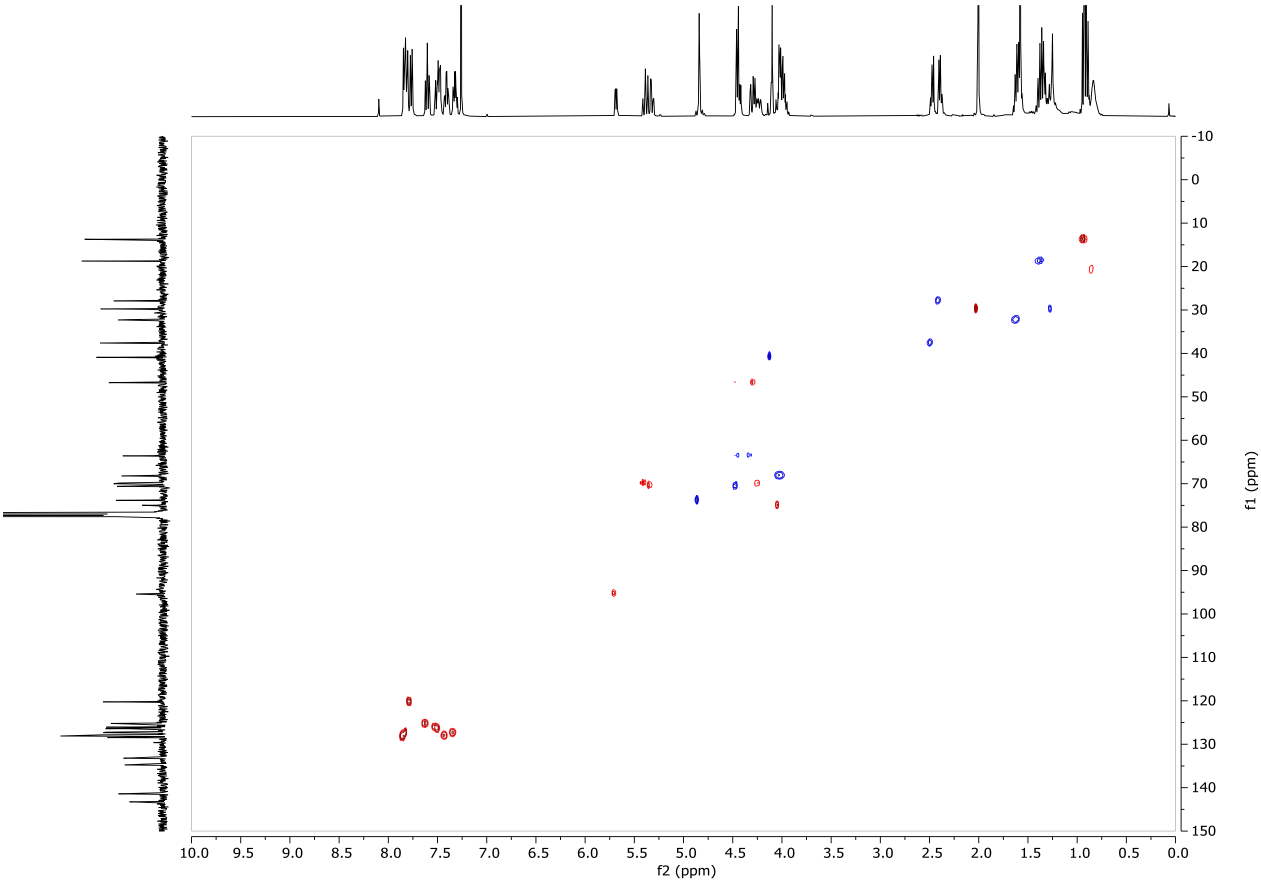

Coupled $^{13}C,^{1}H$ HSQC of **7**:

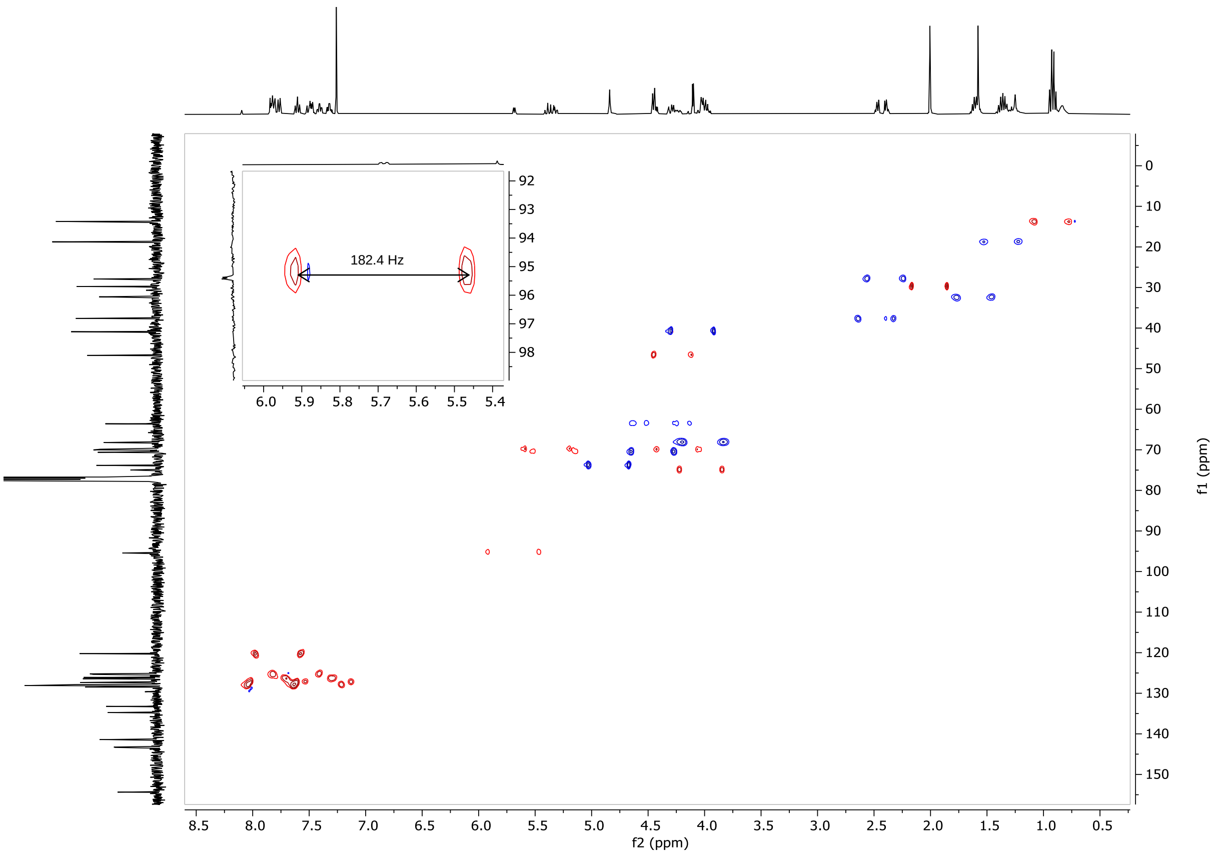

$^{1}H,^{1}H$ COSY of **7**:

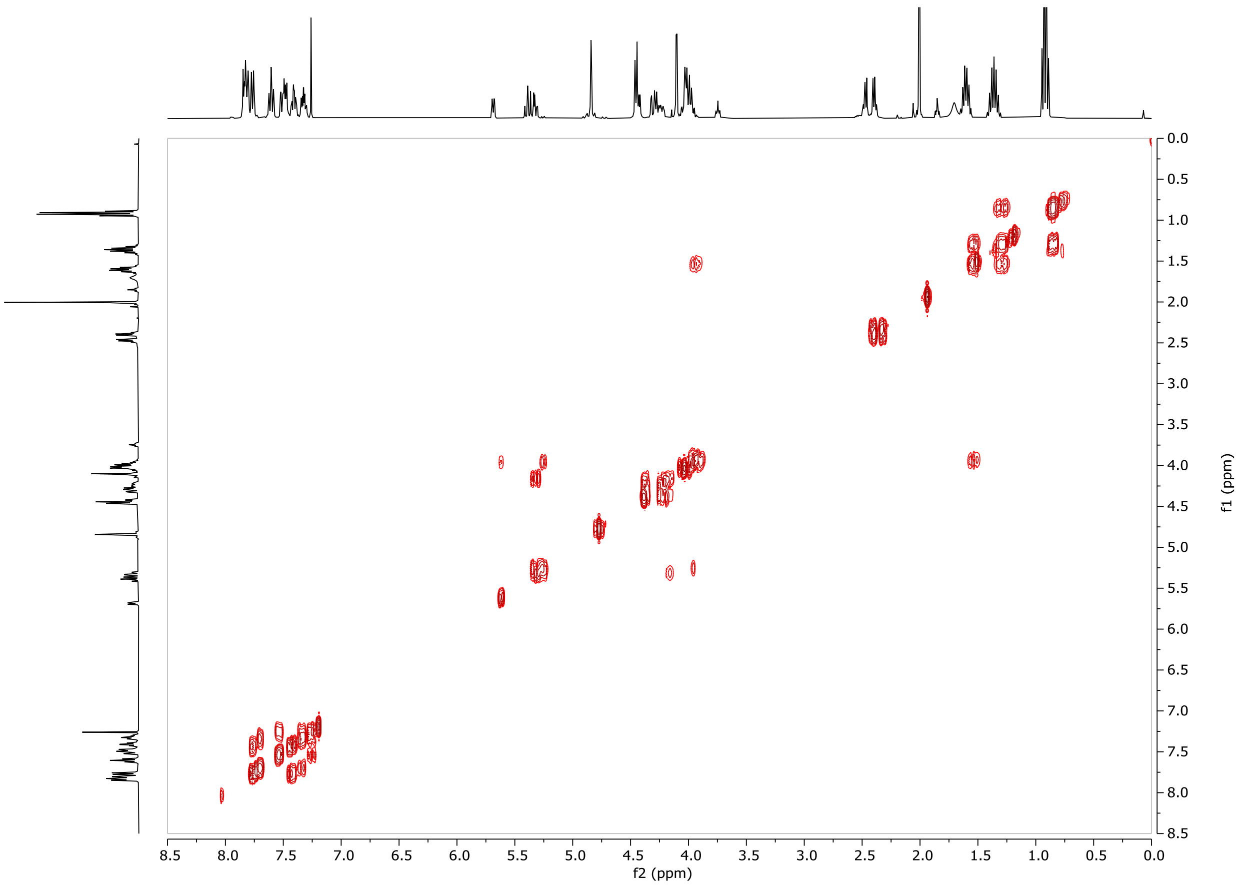

References

1. Le Mai Hoang, K. et al. Traceless Photolabile Linker Expedites the Chemical Synthesis of Complex Oligosaccharides by Automated Glycan Assembly. *Journal of the American Chemical Society* **141**, 9079-9086 (2019).
2. Gude, M., Ryf, J. & White, P.D. An accurate method for the quantitation of Fmoc-derivatized solid phase supports. *Letters in Peptide Science* **9**, 203-206 (2002).
